# Effectiveness of a smartphone-delivered Approach-Avoidance intervention in dietary behavior - a randomized controlled trial

**DOI:** 10.1186/s12966-025-01836-2

**Published:** 2025-11-28

**Authors:** Matthias Burkard Aulbach, Mareike Roettger, Hannah van Alebeek, Sercan Kahveci, Jennifer Schmidt, Jens Blechert

**Affiliations:** 1 Department of Psychology and Center for Cognitive Neuroscience, Paris-Lodron-University of Salzburg, Salzburg, Austria; 2 Muenster Department of Health, FH Münster University of Applied Sciences, Münster, Germany

**Keywords:** Cognitive bias modification, Approach bias modification, Approach-avoidance task, Dietary change, Eating behavior, MHealth, Restraint eating, Intervention, Food craving, Ecological momentary assessment, Randomized controlled trial

## Abstract

**Background:**

Given the therapeutic potential of Approach-Avoidance interventions (AAIs) in the alcohol domain, research has increasingly applied them to the food domain. In AAIs, harmful stimuli are avoided while healthy ones are approached, for example by respectively moving a phone away from or towards oneself.

**Methods:**

We administered a phone-based AAI six times over two weeks to 156 participants in a pre-registered randomized-controlled trial to reduce intake of six “decrease-foods” and increase intake of six “increase-foods”, selected according to each participant’s individual dietary goals. The control group received a placebo task in which all stimuli were equally often approached and avoided. Food craving and intake were the outcomes, measured daily during the training period, four days before and after, and once during a follow-up one month after training. Per-food approach bias was recorded before and after training, and at follow-up.

**Results:**

Compared to placebo, active training reduced the level of decrease-food craving (b = -0.19; 95% HDI [-0.31, -0.05]) without affecting how often craving occurred. Restrained eaters (b = -0.19; 95% HDI [-0.36, -0.03]) and those with low past dietary success (b = 0.13; 95% HDI [0.03, 0.24]) showed the strongest craving strength reduction. Active training also reduced approach bias for decrease-foods, albeit with weaker evidence (b = -38.10; 89% HDI [-73.82, -0.39]). We found no intervention effects on increase-foods on any outcome. There were no interpretable training effects for food intake and no changes were maintained at follow-up.

**Conclusions:**

A multisession mobile AAI reduced craving intensity for foods that participants wanted to eat less of in the 4 days after the end of the intervention period with reductions bouncing back at 4-week follow-up. It remains to future research how this can be sustained long-term and effectively translated into reduced food intake.

**Trial registration:**

This study was registered in the German Clinical Trials Register, ID DRKS00030780.

**Supplementary Information:**

The online version contains supplementary material available at 10.1186/s12966-025-01836-2.

## Background

Overweight and obesity have become major health concerns in many countries [1, 2], with the main cause for overweight being an imbalance between energy input and output [3, 4]. Calorie-dense foods are readily available in obesogenic environments, which often leads to excessive consumption and insufficient intake of healthier foods such as fruits and vegetables. While many people know that diet, overweight, and health are related, and many people intend to adhere to healthier diets [5], they often fail to actually follow through on these intentions [6].

Many psychological models struggle to explain this phenomenon, as they model behavior mainly as originating from thoroughly reflected intentions [7–9]. Dual-process models address this shortcoming by emphasizing that behavior is not only determined by such reflective processes but also by impulsive ones [10, 11]. These models have led to the development of computer-based reaction time (RT) tasks that aim to measure such impulsive determinants of behavior. One such task is the Approach-Avoidance Assessment task (AAA), in which participants respond to images of different categories (e.g., palatable vs. non-palatable foods or objects) on a screen by performing movements that represent approach or avoidance, such as pulling or pushing a joystick [12] or moving a smartphone towards or away from their body [13]. For appetitive foods and other craved or positive stimuli, RTs are often faster for approach trials than for avoidance trials, and this approach advantage for appetitive stimuli is often greater than for neutral reference stimuli; this difference between differences is termed an “approach bias”. An approach bias for food is generally found in healthy individuals, and not in those with anorexia nervosa [14, 15]. This food approach bias is responsive to homeostatic need, as it is higher before than after a meal [16]; further, it is stronger in individuals with high trait food craving [17], external eating, emotional eating [18], and higher body mass index [19]; and it is stronger towards chocolate in those with currently strong state chocolate craving [20]; more generally, an individual’s approach bias for specific foods is stronger if they desire to eat that food [21, 22]. Food approach bias also predicts increased food consumption in impulsive individuals [23] and in people prone to external or emotional eating [24].

### Past Approach-Avoidance interventions and their pitfalls

Moving from observation to intervention, researchers have devised ways to intervene on consumption behavior using an adapted task setup. In Approach-Avoidance *Interventions* (AAI), images are systematically paired with specific reactions, usually unbeknownst to the participant: participants for example always have to approach healthier and avoid less healthy foods. After successful demonstrations of effectiveness in the alcohol domain [25, 26], such interventions have been applied in the food domain and have been shown to effectively reduce food craving [27] as well as selection and consumption of trained foods [28–31], at least for some people and under certain circumstances [32–36]: For example, interventions are more effective for those who struggle with self-regulation [34, 37]. One study even found a reduction in body weight in obese individuals [35]. These findings suggest that these interventions may have therapeutic potential in reducing unhealthy food intake especially in those who struggle with their diet. Not all interventions have been effective however [32], spurring discussions about reasons for a lack of effectiveness, and three prominent aspects have arisen, which we discuss in the following.

First, the exact instructions and setup of the task differ between studies such that participants either approach or avoid based on the stimulus category (“relevant-feature” AAA/AAI) or some other aspect of the image (e.g. its spatial orientation; “irrelevant-feature” AAA/AAI). Since a relevant-feature task focuses attention on the image, it performs better as an approach bias measure than the irrelevant-feature version [38–40]. While data for relevant-feature AA interventions are rare [29, 41, 42], it seems likely that they would be superior to irrelevant-feature AAIs due to this aspect of attentional focus. Second, many studies present pre-selected images that are not personalized to the participant. This likely dilutes training effectiveness, as some participants may not like a given food, or have no intention of changing their intake of it. Therefore, allowing participants to select images of foods they would like to eat less should help in enhancing effectiveness. However, intentions for behavior change have mostly been ignored in this literature. As a third reason for mixed findings, most studies have delivered only single intervention sessions in a laboratory or online; delivering an AAI repeatedly should enhance effectiveness [43, 44]. Smartphones easily enable this and have the further advantage of bringing the intervention closer to day-to-day consumption behavior.

In addition to these three potential reasons limiting the effectiveness of AAIs, most AAI studies to date mainly focused on *reducing* the intake of unhealthy foods, somewhat neglecting the fact that attaining a healthier diet could also require *increasing* the intake of healthier foods. This is not only important for health reasons, as many people fail to consume the recommended amount of fruits and vegetables [45]; it is also important from a behavior change perspective, as it is more effective to substitute an unwanted behavior with an alternative, similar behavior, than it is to simply suppress the unwanted behavior [46–48]. AAIs are promising in this regard as they can be programmed to train both approach of healthy foods and avoidance of unhealthy foods simultaneously, and could thus facilitate such behavior substitution [49].

### The current study

In the current study we deploy a mobile AAI that addresses these shortcomings of prior studies, and we investigate whether this AAI is able to effectively support dietary changes. Specifically, we deliver a smartphone-based multisession relevant-feature AAI that allows participants to select which food images they will work with during the study based on their intention to change how often they eat them. This results in two stimulus sets: individualized foods that participants want to eat more (“increase-foods”) and those they want to eat less (“decrease-foods”). The AAI is accompanied by twice-daily ecological momentary assessment (EMA) that includes measures of intake, food craving, and other relevant day-level variables. To control for potential placebo effects that may come with repeated performance of an eating-related behavioral task, we compare this to an active control in which increase-foods and decrease-foods are approached and avoided equally often. In the assessment of approach bias, we include neutral control stimuli to ensure that approach bias for increase-foods can be separately interpreted/calculated from the approach bias of decrease-foods. As preregistered^1^, we expect that participants in the AAI will display stronger increases in consumption of, craving for, and approach bias towards increase-foods and stronger decreases in consumption of, craving for, and approach bias towards decrease-foods compared to the control condition. We also explored potential person-level moderators of the effect to determine which population benefits most from AAI.

## Methods

The full methods of this randomized controlled trial (RCT) are outlined in the corresponding protocol paper [50] and the preregistration https://osf.io/4k3q9/. Here, we only describe the aspects of the trial relevant to this study and refer the reader to those documents for more detail.

### Participants

We recruited 157 participants (132 women, 25 men, *M*_age_ [*SD*] *=* 27.6 [9.73]; see protocol paper for power analysis [50]) through university and social networks, and through word of mouth. Participants had to be between 18 and 60 years old, not pregnant, and report living without an eating disorder. Participants needed to have a goal of changing their eating behavior in the upcoming weeks. Six participants were excluded from all analyses as their AAI/AAA sessions were all either missing, or excluded (see below for exclusion criteria for AAI/AAA sessions; see consort flowchart in the supplementary materials). One participant was excluded from all analyses that had approach bias scores as the outcome of interest, as this participant seemed to have technical issues with the smartphone application.

### Questionnaires

For each scale we report both Cronbach’s alpha using the R-package psych [51], and McDonald’s omega with bootstrapped 95% confidence intervals using the R-package MBESS [52].

#### Perceived Self-Regulatory success in dieting scale (PSRS)

The Perceived Self-Regulatory Success in Dieting Scale (PSRS) [53] is used to differentiate between successful and unsuccessful dieters. It consists of three items rated on a 7-point Likert scale with higher scores indicating more successful self-regulation. Internal consistency was questionable: ω = 0.69 [0.56, 0.76], α = 0.68.

#### Dutch eating behavior questionnaire restrained eating (DEBQ-res) and external eating (DEBQ-ext) subscales

From the Dutch Eating Behavior Questionnaire (DEBQ) [54], we administered the subscales of restrained eating (DEBQ-res) and external eating (DEBQ-ext). Each subscale consists of 10 items and is rated on a 5-point Likert scale from 1 (never) to 5 (very often). Internal consistency was good for both the restrained eating subscale: ω = 0.89 [0.86, 0.91], α = 0.89; and the external eating subscale: ω = 0.85 [0.80, 0.89], α = 0.85.

#### UPPS impulsive behavior scale (UPPS)

The UPPS impulsive behavior scale (UPPS) [55] consists of four subscales that each measure a facet of impulsivity: urgency, lack of pre-meditation, lack of perseverance, and sensation seeking. Each subscale consists of five statements rated on a 4-point Likert scale from 1 (strongly disagree) to 4 (strongly agree). Higher scores on an UPPS subscale are interpreted as a higher level of impulsivity in the respective impulsivity facet. Internal consistency was questionable for the urgency subscale, ω = 0.68 [0.57, 0.77], α = 0.68; but acceptable for the other three subscales, pre-meditation: ω = 0.74 [0.65, 0.81], α = 0.73; perseverance: ω = 0.79 [0.72, 0.85], α = 0.79; sensation seeking: ω = 0.73 [0.65, 0.79], α = 0.70.

### Materials and procedure

Participants were recruited for a program to support their dietary goal pursuit. They gave informed consent online (which included an explanation that they would be randomized to one of two groups that slightly differed in the type of presented task without specifying that we expected one of them to be less effective), followed by a range of questionnaires including demographics. After the questionnaires, they rated 90 preselected images of foods and drinks from the food.pics [56] and CROCUFID [57] databases, photographs taken by the study authors, and license-free online stock photograph databases. The ratings included two questions for each image: “In the last three weeks, on how many days have you eaten/drunk this food/drink?” (recent intake) and “In the next three weeks, on how many days do you want to eat/drink this food/drink?” (intended intake). Answers to both questions were indicated on a slider ranging from 0 to 21 days. We sampled two categories of stimuli from the 90 images on the basis of these ratings: among the foods participants wanted to eat more than currently, we selected those with the biggest discrepancy between current and intended consumption as “increase-foods”; and among the foods participants wanted to eat less than currently, we selected those with the biggest discrepancy as “decrease-foods”. A random selection of four images per category was included in the AAA/AAI. A randomly selected 8 out of 12 images of office items from the food.pics [56] and FRIDa [58] databases served as control stimuli. We excluded participants that did not have six decrease-foods which they consumed at least twice weekly. At this point, an R-script randomized participants to either the intervention or control group with the condition unknown to the study team.

**Fig. 1:**
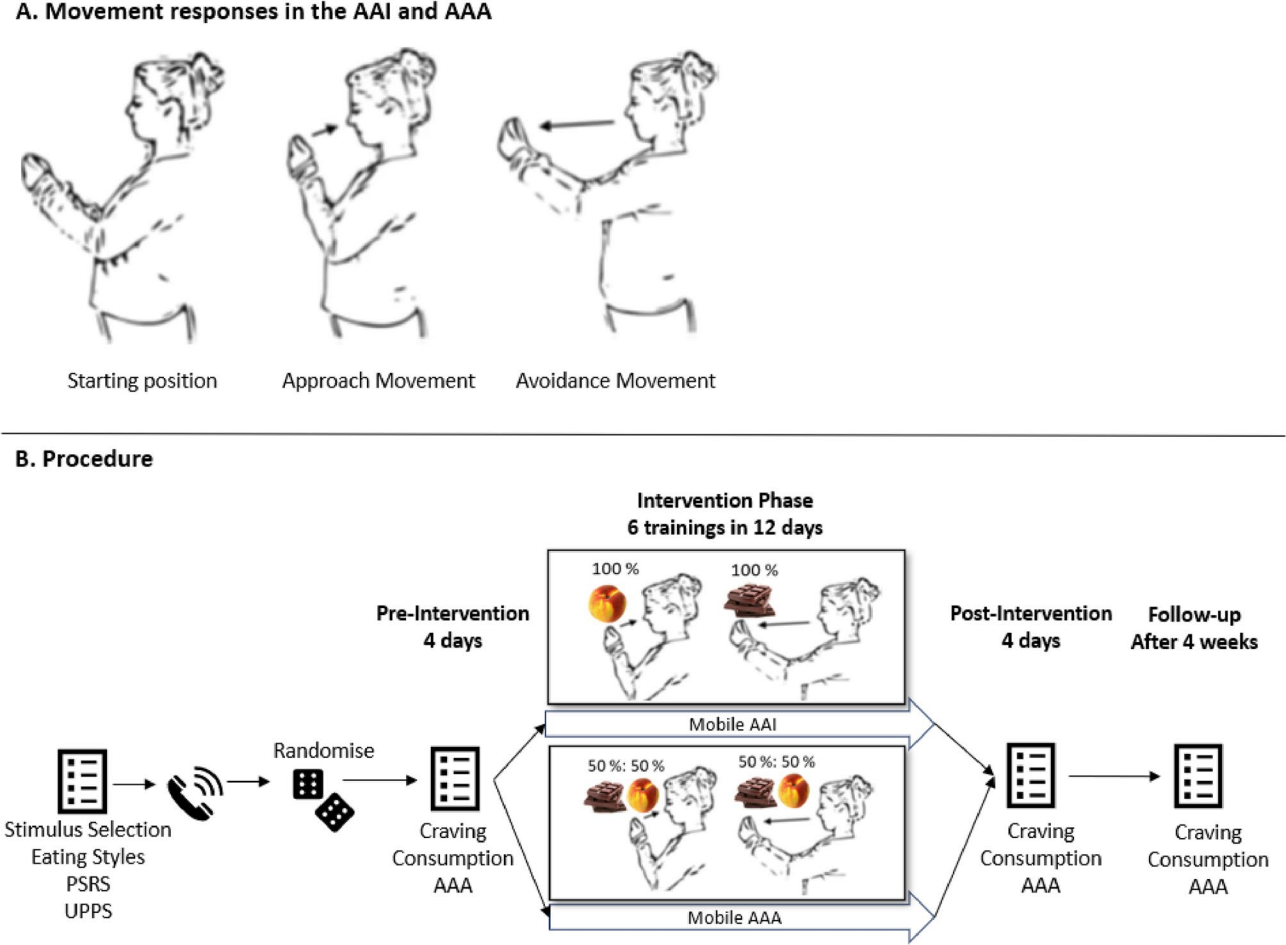
Mobile AAI/AAA and Procedure. Note. AAI = Approach-Avoidance Intervention, AAA = Approach-Avoidance Assessment Task. Panel **A** adapted from [13]

Next, a member of the research team conducted a setup call with each participant to install and explain the use of two smartphone applications: m-path [59] for EMA, and the app to conduct the AAA/AAI. The remainder of the study consisted of four phases (Fig. 1B): a baseline phase on days 1–4, an intervention phase on days 5–16, a post-intervention phase on days 17–20, and a follow-up phase 4 weeks after day 20. Participants completed a single measurement-AAA during the baseline phase (day 4) and also during the post-intervention phase (day 17), while performing an AAI or AAA every other day during the intervention phase, depending on condition (days 6, 8, 10, 12, 14, and 16). EMA was collected throughout these three phases. The follow-up consisted of a single EMA prompt and AAA measurement (day 48, four weeks after the last post-intervention day).

For the AAA/AAI, we used a modified smartphone application [13, 60]. In this task, users held the phone horizontally in front of them while responding to stimuli shown on the screen. After a fixation dot for 1500 ms at the start of the trial, the app presented a food or object stimulus. Depending on image type (food or object), participants had to move the phone towards or away from themselves, representing an approach or avoidance response, respectively.

Two types of Approach-Avoidance Tasks were administered: measurement-AAAs and training-AAIs. Each measurement-AAA block consisted of 24 trials each, while each training-AAI/AAA consisted of 16 trials per block. Each session consisted of 4 blocks, with each block preceded by 4 practice trials. Before each block, instructions were presented, with blocks 1 and 3 instructing participants to approach foods and avoid objects (“approach-food-blocks”) and blocks 2 and 4 giving the opposite instruction (“avoid-food-blocks”). In the AAI (intervention condition), the approach-food-blocks exclusively featured increase-foods and objects, and the avoid-food-blocks exclusively featured decrease-foods and objects; thus, increase-foods were always approached while decrease-foods were always avoided. In the AAA (control condition), all food images were presented in each block. During the intervention phase (days 5–17), after the mid-day prompt (content not relevant here), participants were prompted to conduct a session of AAA/AAI on every other day (days 6, 8, 10, 12, 14, 16) resulting in six AAA/AAI sessions. In addition, all participants conducted an AAA on days 4 and 17 and during the follow-up. Completing one session took about 5 min.

Throughout the study, participants followed a twice-daily EMA schedule, with the mid-day prompt just before their typical lunch time and the other at the end of the day (timing agreed upon individually) with all EMA items using a virtual 0–100 slider (where not reported differently). Relevant items measured *craving* for (“How strongly have you been craving this food today?”) and *intake* of (“How much have you eaten of this food today?”) each of the included foods.

#### Data preprocessing and analysis

##### Approach bias

To pre-process the AAA data, we removed trials with non-responses, movements in the wrong direction, or RTs above 2000 or below 200 ms. After this, exclusions continued within each session within each participant, as we removed trials that deviated more than 3 SDs from the mean. AAA sessions with more than 25% of trials missing or removed were then excluded from analysis entirely.

Separately for approach and avoidance trials as well as for sessions, RTs were averaged across AAA blocks and all object stimuli. For foods, RTs were averaged across AAA blocks only. The average approach or avoid response for objects on a session was subtracted from stimulus-specific food approach or avoidance response on that session to achieve single-difference approach and avoidance scores:

$$\begin{aligned} \mathrm{Stimulus}-\mathrm{specific}\;\mathrm{approach}=&\lbrack\mathrm{food}-\mathrm{specific}\;\mathrm{approach}\rbrack\\&-\lbrack\mathrm{average}\;\mathrm{object}\;\mathrm{approach}\rbrack \end{aligned}$$

$$\begin{aligned} \mathrm{Stimulus}-\mathrm{specific}\;\mathrm{avoidance}\;=&\;\lbrack\mathrm{food}-\mathrm{specific}\;\mathrm{avoidance}\rbrack\\&-\lbrack\mathrm{average}\;\mathrm{object}\;\mathrm{avoidance}\rbrack \end{aligned}$$

Double-difference scores were used to achieve a full bias score per food stimulus and session: [food-specific avoidance]- [food-specific approach]- [average object avoidance]- [average object approach]. Hence, positive values imply the food was approached faster than it was avoided, relative to the difference between approach and avoidance RTs for objects.

#### Analysis

As multiple datapoints are nested within participants and stimuli, we used multilevel models (MLM) to assess the hypotheses. Since our outcome measures craving and intake displayed strong skew and many zeroes, we used Bayesian multilevel two-part hurdle models [61]. In brief, these regression models consist of a hurdle part akin to logistic regression that estimates the probability of the outcome not being zero, and a continuous part following a gamma distribution that estimates the size of the non-zero values. The hurdle model therefore allows to separately determine which variables predict whether intake or craving occurred and, if so, how much was eaten/how strong the craving was. We used brms’s default flat uniform priors for all analyses. To test the robustness of our results we re-ran all models with different priors favoring the null hypothesis (i.e., no relationship between predictor and outcome). 2 Since these models flexibly deal with missing values they approximate an intention to treat analysis with multiple imputation. Note that missing data were rare and similarly spread across groups (see Table 1), making selective drop out unlikely.

**Table 1:** Descriptive statistics and EMA compliance by group

	Training group	Placebo control group
Sample size	72	79
*Demographics*		
Women (%)	60 (83.33%)	68 (86.08%)
Age in years (SD)	25.61 (8.16)	29.66 (10.85)
BMI (kg/m^2^)	23.49 (6.22)^a^	24.47 (3.95)
*Compliance*		
N completed training sessions (out of 6)	5.51	5.57
Baseline EMA compliance (mean N completed questionnaires of 4 assessment points)	3.79	3.72
Post-intervention EMA compliance (mean N completed questionnaires of 4 assessment points)	3.58	3.60
*Means (SDs) for questionnaire scores*		
PSRS	3.56 (1.11)	3.53 (1.23)
DEBQ-restrained eating	2.31 (0.73)	2.60 (0.75)
DEBQ-external eating	3.37 (0.70)	3.39 (0.61)
UPPS lack of perseverance	3.06 (0.57)	2.90 (0.64)
UPPS lack of premeditation	3.04 (0.54)	2.98 (0.56)
UPPS urgency	2.20 (0.56)	2.28 (0.53)
UPPS sensation seeking	2.53 (0.64)	2.34 (0.73)
*Means (SDs) for baseline phase (4 days)*		
Intake decrease-foods (Slider rating 0–100)	19.3 (11.3)	20.2 (11.7)
Intake increase-foods (Slider rating 0–100)	16.6 (11.4)	14.1 (10.6)
Craving decrease-foods (Slider rating 0–100)	32.7 (18.3)	35.3 (16.7)
Craving increase-foods (Slider rating 0–100)	23.8 (14.7)	25.3 (15.5)
*Means (SDs) for intervention perception*		
Expectancy	2.52 (0.96)	2.41 (1.00)
Contingency perception for trained decrease-foods	48.9 (20.9)	52.5 (14.8)
Contingency perception for trained increase-foods	31.2 (19.5)	42.0 (19.5)

Note: *PSRS* Perceived Self-Regulatory Success in Dieting Scale, *DEBQ* Dutch Eating Behavior Questionnaire, *UPPS* Urgency, (lack of) Premeditation, (lack of) Perseverance, Sensation Seeking

^a^Due to apparently interchanged data for height and weight of one participant, we excluded this participant from the BMI computation. N = 71.

For each model, we report the regression coefficient of interest as well as both the 95% and the 89% highest density interval (HDI), reflecting the most credible values of the respective model parameter. This is equivalent to a 5% or 11% alpha level, respectively. If the 89%-HDI does not include 0, we describe the model parameter as being estimated as “significantly” above or below 0.

Analyses include only data from the baseline and post-intervention phase (4 days each) and include all 12 food stimuli. In separate models, we respectively predicted the stimulus-level intake, craving, and approach bias scores for increase and decrease-foods separately using the interaction between Intervention group (0 = 50/50 AAA vs. 1 = 100/0 AAI) and time (0 = Pre vs. 1 = Post) according to this formula:

$$\begin{aligned} &Intake/Craving/Approach\;Bias(increase/decrease\\&-foods)\;\sim\;Intervention\;Group\;\ast\;time\;\\&+\;(time\;\vert\;Subject)\;+\;(time\;\vert\;Stimulus) \end{aligned}$$

To assess intervention effects to follow-up, we then ran equivalent models containing only data from post-intervention and follow-up for all outcomes as well as models containing only data from pre-intervention and follow-up to be able to calculate all contrasts. For moderation analyses we added the relevant moderator and all interaction terms to the models comparing pre- to post-intervention values. We computed post-hoc contrasts using the R-package emmeans [62] for all models where we found evidence of an effect to assess between- and within-condition differences. We report the estimate as well as the 95% HDI for the contrasts of interest.

## Results

We checked for differences in compliance between the two groups with two-sample t-tests. There was no difference in baseline EMA compliance (*t*(143.09) = −0.82, *p* =.41), training compliance (*t*(149) = 0.32, *p* =.75) or post-intervention EMA compliance (*t*(145.99) = 0.16, *p* =.87).

### Intervention effects from pre- to post-intervention and follow-up

#### Intake: no group differences

There was no significant time × condition interaction on intake for both *increase-food*s or *decrease-food*s for hurdle and continuous parts both in the pre-post and the post to follow-up-analyses. Test statistics and plots showing these results can be found in the supplementary materials.

#### Craving: reduced for decrease-foods

**Fig. 2:**
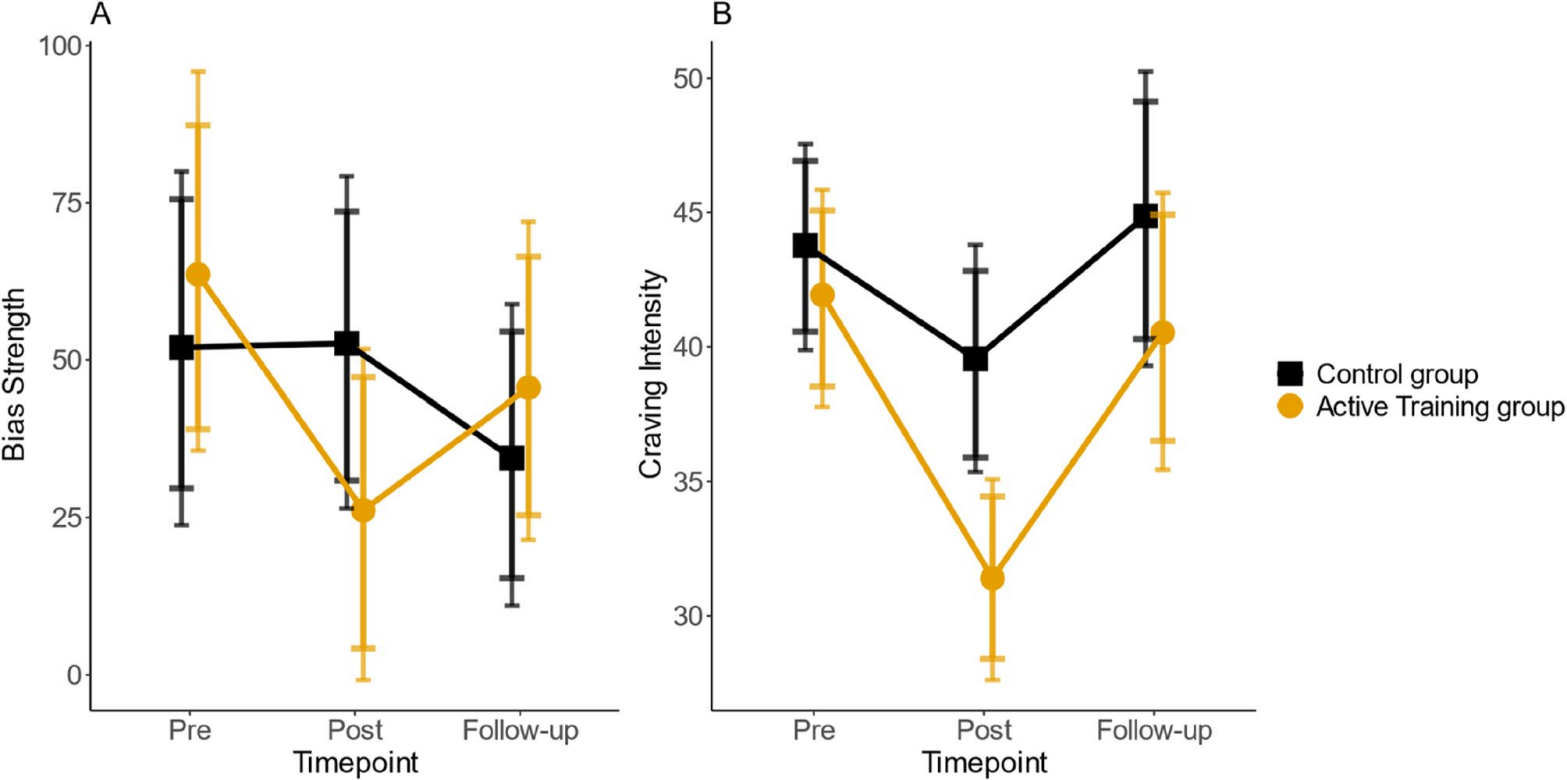
Long-term effects on bias strength as well as craving intensity for decrease-foods. Note: These plots were created based on the model results of the pre- to post-intervention models as well as the post-intervention to follow-up models. Panel A shows the model-based estimates for the means of bias strength, Panel B shows the model-based estimates for the means of craving intensity. The error bars show the 89% HDI and the 95% HDI around these means

We separately report changes in the likelihood of reporting (no) craving (hurdle part) 3, and changes in craving intensity when any craving above zero is reported (continuous part). We found no time x condition effect for the probability of experiencing craving (*n* = 151, hurdle part: b = −0.25; 89% HDI [−0.62, 0.12]; 95% HDI [−0.70, 0.20]). However, the intervention group showed stronger reductions in craving intensity for *decrease-foods* compared to the control group from pre- to post-intervention with the 95% HDI excluding zero (*n* = 151, continuous part: b = −0.19; 89% HDI [−0.29, −0.08]; 95% HDI [−0.31, −0.05], Fig. 2, panel B). This result was robust even with different priors favoring H0 (see Supplements). Regarding *increase-food*s, again, there was neither a significant time × condition interaction for the probability of experiencing craving nor for craving intensity (*n* = 151, hurdle part: b = −0.31; 89% HDI [−0.70, 0.06]; 95% HDI [−0.77, 0.16]; continuous part: b = −0.04; 89% HDI [−0.19, 0.09]; 95% HDI [−0.21, 0.12]).

When looking at the effects from post-intervention to follow-up 4 weeks later, we found a significant interaction for craving intensity (but not probability) for *decrease-foods* on a 89% HDI level (*n* = 149, hurdle part: b = 0.06; 89% HDI [−0.39, 0.51]; 95% HDI [−0.51, 0.61]; continuous part: b = −0.13; 89% HDI [−0.24, −0.003]; 95% HDI [−0.27, 0.02]), indicating that the intervention group’s craving intensity increased significantly more than the control group’s from post-intervention to follow-up. We found no effects for increase-foods (*n* = 149, hurdle part: b = −0.03; 89% HDI [−0.59, 0.27]; 95% HDI [−0.70, 0.36]; continuous part: b = −0.16; 89% HDI [−0.15, 0.10]; 95% HDI [−0.18, 0.12].

Post-hoc contrasts showed evidence for a difference in the craving intensity between the control group and the intervention group at the post-intervention measurement (b = 0.23; 89% HDI [0.11, 0.35]; 95% HDI [0.08, 0.38]), but the apparent difference in the raw values at follow-up did not prove statistically reliable (b = 0.10; 89% HDI [−0.03, 0.24]; 95% HDI [−0.07, 0.26]). The craving intensity within the intervention group decreased from baseline to post-intervention measurement (b = 0.29; 89% HDI [0.21, 0.37], 95% HDI [0.19, 0.38]) and increased again from post-intervention to follow-up measurement (b = 0.23; 89% HDI [0.14, 0.32]; 95% HDI [0.12, 0.34]). However, there was no significant change from baseline to follow-up within the intervention group (b = 0.09; 89% HDI [−0.002, 0.18]; 95% HDI [−0.002, 0.20]) or within the control group (b = −0.003; 89% HDI [−0.09, 0.09]; 95% HDI [−0.11, 0.11]).

#### Approach bias: reduced for decrease-foods

For *decrease-food*s, the group × time interaction for bias scores from baseline assessment to post-assessment was significant on an 89% HDI level (*n* = 144, b = −38.10; 89% HDI [−73.82, −0.39]; 95% HDI [−81.88, 8.29]). Bias for decrease-foods decreased more in the intervention group than in the control group (Fig. 2, panel A). This result was not robust with priors mildly favoring H0 (see Supplements). There was no significant interaction for *increase* foods (*n* = 144, b = −15.92; 89% HDI [−59.81, 28.95]; 95% HDI [−67.82, 40.36]). The models examining changes from post-intervention to follow-up also showed a significant effect for *decrease-food*s on an 89% HDI level (*n* = 141, b = 35.06; 89% HDI. [0.98, 66.89]; 95% HDI [−5.61, 75.64]), indicating a stronger increase of bias values in the intervention group than in the control group. Again, there was no effect for *increase-food*s (*n* = 142, b = 11.06; 89% HDI [−24.84, 49.27]; 95% HDI [−32.84, 57.55]).

Post-hoc contrasts did not show evidence for a group difference in bias scores for *decrease-food*s at the post-intervention measurement (b = 23.67; 89% HDI [−3.18, 51.09]; 95% HDI [−8.53, 58.0]) or at follow-up measurement (b = −11.09; 89% HDI [−39.53, 15.16]; 95% HDI [−45.37, 20.8]). Bias scores within the intervention group decreased from baseline to post-intervention measurement (b = 37.55; 89% HDI [8.09, 65.90], 95% HDI [2.16, 73.40]), while there was no significant increase from post-intervention to follow-up measurement (b = −19.43; 89% HDI [−44.35, 5.71]; 95% HDI [−51.60, 10.10]). However, there was also no significant decrease in bias from baseline to follow-up within the intervention group (b = 17.2; 89% HDI [−12.00, 48.14]; 95% HDI [−19.7, 53.3]) or withing the control group (b = 17.0; 89% HDI [−11.30, 45.38]; 95% HDI [−17.2, 52.4]).

### Moderation of craving effects by participant characteristics: restraint and perceived self-regulatory success matter

**Fig. 3:**
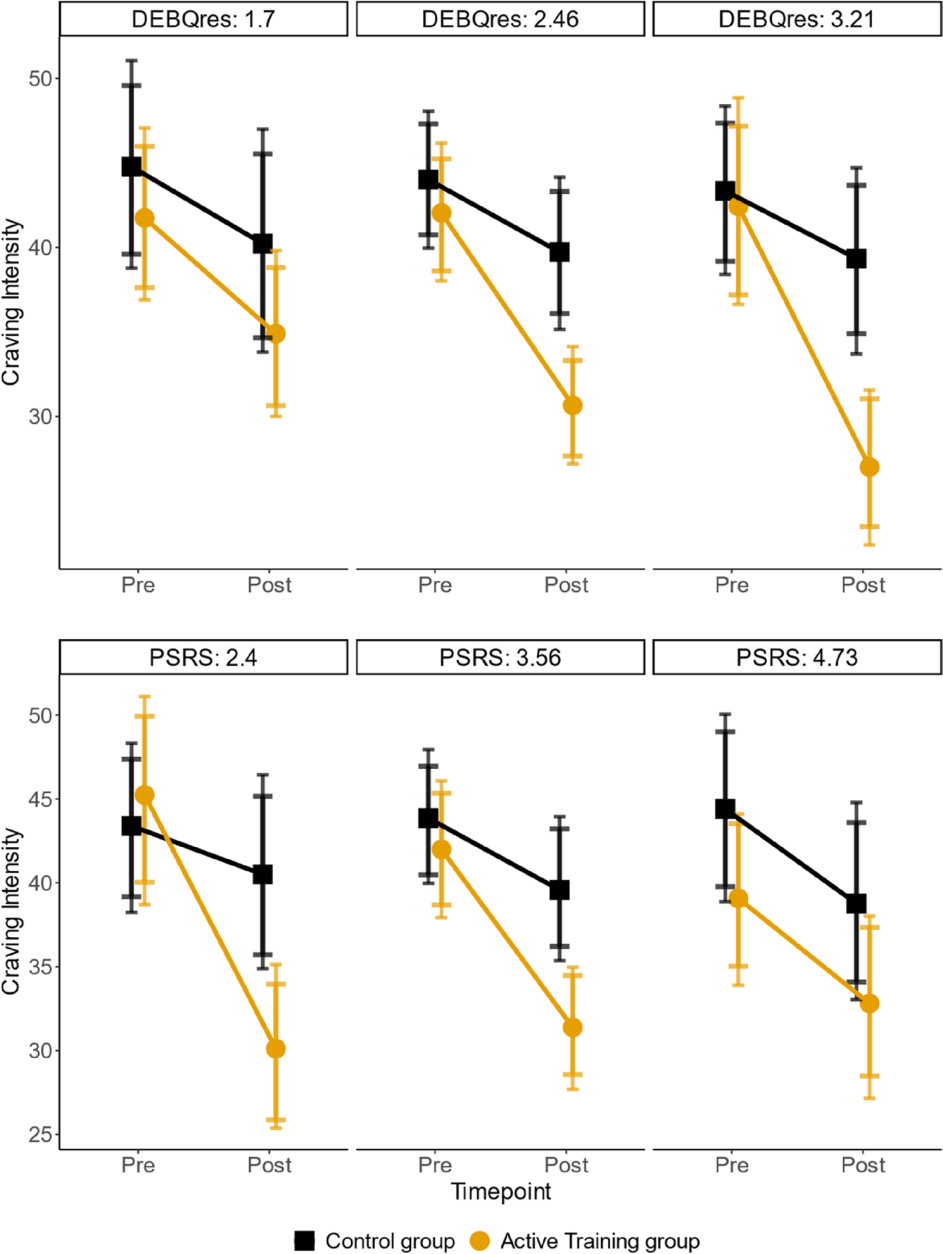
Moderation of training effectiveness by restrained eating and perceived self-regulatory success. The graphs show the model-based estimates for the means and the 89% HDI and the 95% HDI around these means

Participants’ restrained eating and perceived self-regulatory success moderated the baseline to post-training change in craving intensity for *decrease-foods* (*n* = 151, three-way interaction for restrained eating in the continuous part: b = −0.19; 89% HDI [−0.32, −0.05]; 95% HDI [−0.36, −0.03], Fig. 3 (top); and for perceived self-regulatory success: b = 0.13; 89% HDI [0.04, 0.22]; 95% HDI [0.03, 0.24], Fig. 3 (bottom)). These results were partly robust with priors favoring H0 4 (see Supplements). There was no evidence for an interaction in the hurdle part (restrained eating: b = 0.41; 89% HDI [−0.05, 0.90]; 95% HDI [−0.20, 0.98], perceived self-regulatory success: b = −0.13; 89% HDI [−0.43, 0.20]; 95% HDI [−0.53, 0.24]). The intervention was most effective in reducing craving intensity for participants with high restrained eating and low past perceived self-regulatory success. These two questionnaires were not significantly correlated; *r* (149) = − 0.17, *p* =.091.^5^ The analyses including the UPPS and external eating scales as well as those using other outcomes did not yield significant results (see supplementary materials).

## Discussion

In this RCT, we found that a multisession mobile AAI reduced craving intensity for foods that participants wanted to eat less of in the 4 days after the end of the intervention period. We further found weaker evidence that the intervention reduced approach bias for these foods as well. Both reductions bounced back at the 4-week follow-up, indicating no long-term effectiveness. The AAI did not influence intake, nor did we find effects on foods that participants wanted to eat more of. However, we found that restrained eaters and those with low perceived self-regulatory success in dieting showed the largest reductions in craving intensity for decrease-foods, indicating they might benefit most from the intervention.

The current study was designed to address several shortcomings of earlier research on AAIs. In particular, we expected that the repeated delivery of the intervention (6 sessions/384 trials) would significantly boost its effectiveness. Indeed, the achieved reductions in craving intensity seem promising, as craving is a common obstacle to successful dietary goal pursuit [63, 64] and is perceived as unpleasant, especially in dieters [65, 66]. Like craving, approach bias for decrease-foods also decreased from pre- to post-intervention. This is in line with findings that craving and approach bias are coupled [21, 67] as part of a cognitive-behavioral pattern that prepares for ingestion [65]. Similar tasks have also been shown to reduce food valuation based on neuroimaging [68, 69]. However, we need to note that effects were rather small, with the difference in pre-post changes between control and intervention group for cravings being 6.3 points (on a 100-point slider). The small achieved reductions in craving and (to a less robust extent) approach bias did not result in intake reductions, in line with earlier studies that showed AAIs and similar tasks reduce bias or food liking without changing intake [33, 70]. Several factors might have prevented the craving and bias reduction from translating into consumption reduction: recent research has shown that food cravings are associated with higher self-regulatory efforts, indicating that individuals adjust their self-regulatory efforts to experienced craving strength [71]. If the AAI reduced craving strength, participants might, in consequence, have reduced subsequent self-regulatory effort, leading to similar consumption levels as pre-intervention. In addition, eating behavior is context dependent: availability of craved foods, homeostatic meal planning, and social context are factors that influence (overt) eating [63, 72], but not (covert) craving. Studies that have successfully changed actual eating behavior using the AAI and similar tasks (such as the Go-/No-Go task) have typically been conducted in controlled laboratory settings and thus ignored such real-life circumstances imminent in our EMA design [28, 73, 74].

Regarding long-term effects, group differences disappeared at the four-week follow-up. The rebound towards baseline might indicate that the rather short intervention period was not enough to prompt lasting changes in participants’ reactivity to desirable food stimuli. To what degree continued AAI might help to sustain longer-term intervention effects remains an open question at this point.

We further found two eating behavior traits to moderate the intervention effects on decrease-food craving intensity: perceived self-regulatory success in dieting [53] and eating restraint [54, 75], with both effects being somewhat robust to priors favoring the null hypothesis. Those who reported difficulties in regulating their dieting showed the strongest effects, indicating that AAI could potentially support dietary goal pursuit of individuals who need it the most [34], and may be better suited for that purpose than for enhancing the dieting capabilities of those who are already successful. In light of dual-process models, this might be because those struggling with self-regulation often experience goal-incongruent behavioral impulses and AAIs might help to reduce such impulses. Additionally, independent from self-regulatory success, restrained eaters showed stronger effects than unrestrained eaters, which underlines the importance of having a dietary goal for AAI to have an effect [75]: Restrained eaters care strongly about what they (don’t) eat. AAI might be able to support this dietary goal through reducing craving. This seems particularly important as restrained eaters report stronger tendencies to experience craving on a trait level [76] and respond strongly to perceived breaches of their dietary rules [77, 78]. While restraint eating is clearly not equivalent to caloric dieting, the latter likely brings about similar processes as in restrained eating (dieting goal) and thus future research could apply the present intervention trial to weight reduction dieting.

We had expected to find not only goal-congruent changes for decrease-foods but also in increase-foods, increasing the appeal and consumption of foods participants wanted to eat more of (e.g. for their health benefits), based on earlier findings [49, 79, 80]. However, we found no main effects in this goal-congruent food category on any of our outcome variables. It might be that participants already chose foods they generally liked as their increase-foods and therefore, further increases in craving and approach bias might be hard to achieve.

### Future directions

In addition to the analyses reported here, we had assessed other variables that have been suggested to work as moderators or mechanisms of the effect, including the awareness of contingencies between stimuli and required reactions [49, 81], expectations regarding training effects [82–84], as well as generalization of training effects to non-trained stimuli. However, none of the analyses on these variables yielded conclusive results and we therefore refrain from commenting on these theoretical debates (see the supplementary materials for all analyses). However, we urge future researchers to design their studies explicitly to examine such potential mechanisms to improve our understanding of how AAI effects come about in applied settings.

We make a number of propositions for future research into dietary enhancement through AAI. First, research may benefit from an exclusive focus on restrained eaters with low dietary success, since our training was most effective in reducing craving in this subsample. Second, it may help to get participants to commit to their dietary intentions, as participants in the current study were free to pursue or abandon their dietary intentions. Third, future research could study changes in food intake without the influence of food availability, by ensuring participants always have their decrease and increase-foods available. Fourth, we suggest studying the combination of AAIs with other behavior change techniques that focus on different mechanisms of eating behavior [85].

### Strengths and limitations

This study used a rigorous design to test an intensive, multi-session, mobile version of a task in a field setting that has thus far mainly been tested in laboratory environments. Its strengths thus include (1) high ecological validity due to the mobile nature of the intervention and data assessment; (2) comparison with a closely matched, active control group within a double-blind design to isolate effects of stimulus-action couplings from mere stimulus exposure, task performance, and experimenter as well as tracking effects; (3) a large sample that (4) provided many data points on craving, consumption, and approach bias for (5) a wide range of personalized food stimuli.

Regarding limitations, allowing participants to freely choose food images might have resulted in fuzzy categories of decrease- and increase-foods which might have impaired effectiveness. Similarly, stimulus selections of some participants seem to indicate that the food item selection did not work fully as intended (e.g., one participant choosing ice cream as an increase-food), adding to the fuzziness of the categories. We recommend using a more restricted stimulus set in future studies, for example by limiting stimuli to snack foods and pre-classifying stimuli as possible increase or decrease-foods rather than leaving the choice entirely to participants. In addition, our sample predominantly consisted of young, healthy-weight individuals. We therefore cannot say to what extent our results generalize to other populations, such as those living with overweight or obesity or older individuals who might generally be less comfortable with using mobile applications.

## Conclusion

The mobile AAI tested in this multi-session intervention study reduced craving intensity and approach bias towards goal-incongruent foods throughout the 12-day intervention period but not at four-week follow-up. The effects on craving were most pronounced for those participants who reported to struggle with dietary self-regulation and restrained eaters. While no effects were observed for actual food intake, our study adds to previous work showing the potential of cognitive bias modification interventions in health and psychopathology and instills hope that also eating behavior - being known to be highly resistant to change and multiply determined - can be changed in structured cognitive bias modification tasks that take up nor more than a few minutes per day and that can be disseminated at low costs on a global level. Future research should explore how to improve long-term effectiveness, for example using booster sessions.

## Supplementary Information

Supplementary Material 1

Supplementary Material 2

Supplementary Material 3

Supplementary Material 4

## Data Availability

The datasets generated and/or analysed during the current study are available in the Open Science Framework repository, https://osf.io/4k3q9/files/osfstorage.

## Abbreviations

AAI: Approach-Avoidance Intervention
AAA: Approach-Avoidance Assessment
BMI: Body-Mass Index
DEBQ: Dutch Eating Behavior Questionnaire
PSRS: Perceived Self-Regulatory Success in Dieting Scale
RCT: Randomized-Controlled Trial
UPPS: (Negative) Urgency, (lack of) Premeditation, (lack of) Perseverance, Sensation Seeking

## Footnotes

1 We had preregistered further analyses and report those in the supplementary materials.

2 We first ran a model with a prior assigning a 10 times higher likelihood for the H0 than H1. When this indicated that the estimate’s 89% HDI would include zero, we then ran the same model with a prior assigning a 5 times higher likelihood for the H0 than H1, then 2 times to explore for which priors the effect “disappeared”. We briefly mention those results in the manuscript and refer the interested reader to the Supplementary Materials for more detail.

3 Note that the parameter estimates for the hurdle part relate to the probability that craving is zero, that is, the probability of craving being absent.

4 The moderation by perceived self-regulatory success was robust on a 89% HDI level even with priors that assigned a 10 times higher likelihood to H0 than H1, while the moderation by restrained eating was robust with priors that assigned a 5 times higher likelihood to H0.

5 As pre-registered, we calculated interaction models with additional participant-level, stimulus-level and training-level variables for the changes from pre- to post training. An overview of effects as well as all model results can be found in the supplements. In this paper, we focus on the variables with consistent effects (i.e., effects for which we found evidence within different outcome variables or different similar moderator variables).
