# Supplementary material for: Effectiveness of a smartphone-delivered Approach-Avoidance intervention in dietary behavior - a randomized controlled trial
Source: Int J Behav Nutr Phys Act. 2025 Nov 28;22:153. doi: 10.1186/s12966-025-01836-2 (PMC12664223; doi:10.1186/s12966-025-01836-2)
Supplement: Supplementary file 3 — Supplementary Material 3 [file 12966_2025_1836_MOESM3_ESM.doc]

Assessed for eligibility

(n = 330)

# Enrollment

Excluded (n = 171)

Not finishing the questionnaire

session

(n = 50)

Not meeting inclusion criteria

(n = 121)

Randomized (n = 159)

#

# Analysis

Analyzed (n = 72)

Excluded from bias analysis

(n = 0)

Analyzed (n = 79)

Excluded from bias analysis

(n = 1) (the bias assessment did not work as intended)

**Follow up**

Lost to follow up

(n = 1)

Lost to follow up

(n = 2)

**Post Assessment**

Lost to post assessment

(n = 1)

Lost to post assessment

(n = 0)

Allocated to control group (n = 81)

Received allocated intervention (n = 80)

Did not receive allocated intervention (n = 1)

(did not finish an AAA)

Allocation

Allocated to intervention

(n = 78)

Received allocated intervention (n = 72)

Did not receive allocated intervention (n = 6)

(did not finish an AAA or AAI)
