# Supplementary material for: Effectiveness of a smartphone-delivered Approach-Avoidance intervention in dietary behavior - a randomized controlled trial
Source: Int J Behav Nutr Phys Act. 2025 Nov 28;22:153. doi: 10.1186/s12966-025-01836-2 (PMC12664223; doi:10.1186/s12966-025-01836-2)
Supplement: Supplementary file 4 — Supplementary Material 4 [file 12966_2025_1836_MOESM4_ESM.html]

Supplements


Code 

- Show All Code
- Hide All Code

# Supplements

#### Röttger Mareike

#### 2025-09-12

# Stimuli selection

Images were selected from the food-pics\_extended database (Blechert
et al., 2019), the Crocufid database (Toet et al., 2019), images taken
by ourselves for the study and several free-to-use online databases.
Below is a list of all stimuli, first listing object stimuli and then
listing food stimuli. For each stimulus, a verbal description of the
depicted stimulus is given (the images are available on osf), the image
source and, for all stimuli taken from image repositories, the number
under which the image can be found in the respective repository. In
addition, for the food stimuli, we list the number of participants in
the control group and in the intervention group for which this specific
stimulus was selected as a goal-congruent (increase) or goal-incongruent
(decrease) food.

|  | Number | Verbal Description | source | Number in Source Project | Control group Increase Food | Control group Decrease Food | Intervention group Increase Food | Intervention group Decrease Food |
| --- | --- | --- | --- | --- | --- | --- | --- | --- |
| 1 | f1 | Peanut flips | FoodPics | 152 | 1 | 0 | 1 | 0 |
| 12 | f2 | Mixed muesli | FoodPics | 227 | 12 | 5 | 7 | 5 |
| 23 | f3 | Nut mix | Own fotograph | NA | 15 | 2 | 13 | 2 |
| 34 | f4 | Fish | FoodPics | 307 | 7 | 0 | 2 | 2 |
| 45 | f5 | Salty snacks | FoodPics | 8 | 1 | 11 | 1 | 8 |
| 56 | f6 | Pizza | FoodPics | 131 | 1 | 4 | 1 | 5 |
| 67 | f7 | Honey | FreeImages | NA | 4 | 3 | 1 | 4 |
| 78 | f8 | Chicken nuggets | Pixabay | NA | 0 | 0 | 0 | 0 |
| 89 | f9 | Tofu | Pixabay | NA | 10 | 0 | 7 | 1 |
| 2 | f10 | Paprika | Crocufid | cro-0161 | 14 | 0 | 17 | 0 |
| 3 | f11 | Tea | Pixabay | NA | 14 | 2 | 24 | 1 |
| 4 | f12 | Fish sticks | FoodPics | 302 | 0 | 0 | 0 | 0 |
| 5 | f13 | Mushrooms | Pexels | NA | 11 | 1 | 7 | 0 |
| 6 | f14 | Salty pastries | FoodPics | 800 | 0 | 13 | 0 | 13 |
| 7 | f15 | Cucumber | FoodPics | 267 | 20 | 1 | 31 | 0 |
| 8 | f16 | Quinoa | Pixabay | NA | 19 | 0 | 16 | 0 |
| 9 | f17 | Mashed potatoes | FoodPics | 327 | 5 | 2 | 1 | 0 |
| 10 | f18 | Chocolate nuts | FoodPics | 297 | 0 | 1 | 0 | 4 |
| 11 | f19 | Hummus | Own fotograph | NA | 11 | 2 | 10 | 0 |
| 13 | f20 | Kiwi | Pexels | NA | 6 | 0 | 12 | 1 |
| 14 | f21 | Lasagne | FoodPics | 145 | 1 | 0 | 0 | 0 |
| 15 | f22 | Nuts in batter | Pixabay | NA | 0 | 0 | 0 | 1 |
| 16 | f23 | Zucchini | Pixabay | NA | 13 | 0 | 10 | 2 |
| 17 | f24 | Ice pop | Freepic | NA | 1 | 0 | 1 | 1 |
| 18 | f25 | Lentils | FoodPics | 884 | 8 | 0 | 13 | 2 |
| 19 | f26 | Avocado | FoodPics | 283 | 3 | 4 | 4 | 2 |
| 20 | f27 | Carrots | Pixabay | NA | 16 | 3 | 15 | 1 |
| 21 | f28 | Cappuccino | FoodPics | 472 | 4 | 20 | 1 | 15 |
| 22 | f29 | Salty crackers | Pixabay | NA | 0 | 2 | 1 | 0 |
| 24 | f30 | Milk | FreeImages | NA | 3 | 13 | 2 | 9 |
| 25 | f31 | Pottage | FoodPics | 693 | 19 | 1 | 10 | 0 |
| 26 | f32 | Caramel bar | Pixabay | NA | 1 | 11 | 0 | 10 |
| 27 | f33 | Marmalade | Pexels | NA | 3 | 9 | 0 | 9 |
| 28 | f34 | Spring rolls | Pixabay | NA | 1 | 0 | 0 | 1 |
| 29 | f35 | Butter | FoodPics | 64 | 0 | 26 | 0 | 15 |
| 30 | f36 | Mandarin/ oranges | FoodPics | 221 | 21 | 2 | 30 | 0 |
| 31 | f37 | Cereal bar | FoodPics | 180 | 3 | 7 | 1 | 9 |
| 32 | f38 | Wine gums | FoodPics | 339 | 0 | 9 | 0 | 13 |
| 33 | f39 | schnitzel | FoodPics | 309 | 0 | 1 | 0 | 0 |
| 35 | f40 | Chips | FoodPics | 43 | 0 | 13 | 1 | 14 |
| 36 | f41 | Egg | Pixabay | NA | 2 | 16 | 7 | 2 |
| 37 | f42 | Tortelloni | Unsplash | NA | 1 | 0 | 2 | 0 |
| 38 | f43 | Sausage | Pixabay | NA | 0 | 0 | 0 | 1 |
| 39 | f44 | Cornflakes | Unsplash | NA | 0 | 2 | 2 | 6 |
| 40 | f45 | Chicken meat | FoodPics | 301 | 4 | 6 | 4 | 4 |
| 41 | f46 | Softdrinks | Freepic | NA | 1 | 28 | 0 | 23 |
| 42 | f47 | Popcorn | FoodPics | 294 | 1 | 0 | 0 | 1 |
| 43 | f48 | Pancakes | FoodPics | 16 | 2 | 0 | 0 | 1 |
| 44 | f49 | Banana | Crocufid | cro-0068 | 16 | 6 | 8 | 1 |
| 46 | f50 | Cheese | FoodPics | 82 | 0 | 21 | 1 | 17 |
| 47 | f51 | Dumplings | Pixabay | NA | 2 | 0 | 1 | 0 |
| 48 | f52 | Pralines | Pixabay | NA | 1 | 13 | 2 | 11 |
| 49 | f53 | Burger | FoodPics | 2 | 0 | 1 | 0 | 1 |
| 50 | f54 | Grapes | FoodPics | 281 | 9 | 2 | 12 | 1 |
| 51 | f55 | Gnocchi | Freepic | NA | 1 | 0 | 0 | 1 |
| 52 | f56 | French fries | FoodPics | 22 | 1 | 8 | 0 | 8 |
| 53 | f57 | Waffles | Pixabay | NA | 2 | 1 | 1 | 2 |
| 54 | f58 | Cheese noodles | Pixabay | NA | 1 | 2 | 0 | 0 |
| 55 | f59 | Ice cream | Freepic | NA | 1 | 6 | 0 | 4 |
| 57 | f60 | Sushi | FoodPics | 745 | 3 | 0 | 2 | 0 |
| 58 | f61 | Apples | Crocufid | cro-0003 | 27 | 3 | 26 | 0 |
| 59 | f62 | Salad | Pexels | NA | 34 | 0 | 34 | 2 |
| 60 | f63 | Fruit juice | Pexels | NA | 4 | 7 | 5 | 11 |
| 61 | f64 | Potatoes | FoodPics | 317 | 11 | 8 | 7 | 3 |
| 62 | f65 | Meat loaf | Own fotograph | NA | 0 | 1 | 0 | 1 |
| 63 | f66 | Nachos | FreeImages | NA | 0 | 3 | 0 | 1 |
| 64 | f67 | Milk chocolate | FoodPics | 167 | 1 | 36 | 0 | 36 |
| 65 | f68 | Noodles | Freepic | NA | 0 | 24 | 2 | 24 |
| 66 | f69 | Nougat cream | FoodPics | 189 | 0 | 7 | 0 | 13 |
| 68 | f70 | Wraps | Freepic | NA | 2 | 1 | 3 | 2 |
| 69 | f71 | Biscuits | FoodPics | 4 | 0 | 16 | 3 | 18 |
| 70 | f72 | Berries | FoodPics | 224 | 24 | 1 | 21 | 0 |
| 71 | f73 | Oven baguette | Own fotograph | NA | 1 | 0 | 0 | 0 |
| 72 | f74 | Muffin | FoodPics | 80 | 0 | 0 | 0 | 2 |
| 73 | f75 | Sweet pastries | FoodPics | 126 | 1 | 16 | 0 | 16 |
| 74 | f76 | Sausage | Pixabay | NA | 1 | 13 | 1 | 10 |
| 75 | f77 | Cake | FoodPics, Pixabay | NA | 0 | 5 | 1 | 8 |
| 76 | f78 | Chickpea | Pixabay | NA | 19 | 0 | 7 | 0 |
| 77 | f79 | Dark chocolate | FoodPics | 879 | 3 | 5 | 1 | 6 |
| 79 | f80 | Fried noodles | Pexels | NA | 2 | 1 | 0 | 0 |
| 80 | f81 | Baguette | Pixabay | NA | 0 | 9 | 1 | 2 |
| 81 | f82 | Kebap | Freepic | NA | 0 | 1 | 1 | 0 |
| 82 | f83 | Toast | FoodPics | 469 | 0 | 10 | 1 | 13 |
| 83 | f84 | Beef | FoodPics | 563 | 1 | 2 | 0 | 5 |
| 84 | f85 | Yoghurt | iStock | NA | 14 | 3 | 8 | 3 |
| 85 | f86 | Chocolate wafers | Own fotograph | NA | 1 | 0 | 0 | 0 |
| 86 | f87 | Meatballs | Pixabay | NA | 2 | 1 | 1 | 0 |
| 87 | f88 | Vegetable patties | Own fotograph | NA | 2 | 0 | 6 | 0 |
| 88 | f89 | Wholemeal bread | FoodPics | 839 | 24 | 1 | 18 | 2 |
| 90 | f90 | Chocolate bar | FoodPics | 293 | 0 | 16 | 1 | 18 |
| 91 | o1 | Writing block | FoodPics | 1017 | NA | NA | NA | NA |
| 95 | o2 | Pencil case | FoodPics | 1094 | NA | NA | NA | NA |
| 96 | o3 | Calculator | FoodPics | 1140 | NA | NA | NA | NA |
| 97 | o4 | Stapler | FoodPics | 1142 | NA | NA | NA | NA |
| 98 | o5 | Pens | FoodPics | 1143 | NA | NA | NA | NA |
| 99 | o6 | Clipboard | FoodPics | 1146 | NA | NA | NA | NA |
| 100 | o7 | Paper clips | FoodPics | 1155 | NA | NA | NA | NA |
| 101 | o8 | Envelope | FRIEDa | AO\_036 | NA | NA | NA | NA |
| 102 | o9 | Punch | FRIEDa | AO\_119 | NA | NA | NA | NA |
| 92 | o10 | Eraser | FRIEDa | AO\_120 | NA | NA | NA | NA |
| 93 | o11 | Protractor | FRIEDa | AO\_226 | NA | NA | NA | NA |
| 94 | o12 | Ruler | FRIEDa | AO\_104 | NA | NA | NA | NA |

# Additional plots

All plots based on the raw data, including plots from the
non-significant results for the change in outcome variables from
baseline assessment to the post-intervention assessment to follow-up are
included here. All plots are build based on the raw data and show means
as well as standard errors. Note that for plots showing probabilities,
the y-axis is such that higher values indicate higher probabilities for
occurrence.

## Decrease foods

## Increase foods

## Plots for the interaction with DEBQ and PSRS scores

## Model-based plots for the models concerning Decrease foods

The points represent the means as predicted by the models, while the
error bars represent standard deviations of the predicted means. While
the model-based plots for the described models (i.e., Bias for Decrease
Foods, Craving intensity for Decrease Foods, and moderation of the
latter by PSRS and DEBQres scores) are included in the paper, the other
models are included here.

```
## [1] "Craving probability"
```

```
## [1] "Intake probability"
```

```
## [1] "Amount of Intake"
```

## Model-based plots for the models concerning Increase foods

The points represent the means as predicted by the models, while the
error bars represent standard deviations of the predicted means.

```
## [1] "Bias"
```

```
## [1] "Craving probability"
```

```
## [1] "Craving intensity"
```

```
## [1] "Intake probability"
```

```
## [1] "Amount of Intake"
```

# Statement of the convergence criteria and adaptations in case of convergence issues

For all models, we assessed model convergence by checking the
following criteria for each parameter that was computed by the model:
R-hat < 1.02 and ESS > 400. We also visually checked the
caterpillar plots when model convergence was unclear. In case of
non-convergence we increased the iterations. For the bias analyses, an
adapt\_delta of .99 was used, for the other analyses, we used an
adapt\_delta of .8. In case of divergent transitions, we also increased
adapt\_delta to .95 or .99.

# Sensitivity Analysis

We ran a sensitivity analysis for all models included in the paper
with priors in favor of the H0. Specifically, we first ran the same
models with a prior assigned to the interaction of interest that assigns
a 10 times higher likelihood to H0 than H1. If the 89% HDI of the
interaction of interest included 0 in this model, we further
investigated the robustness of the effect by using a prior that assigns
a 5 times higher likelihood to H0 than H1. We repeated this procedure
once more with a prior that assigns a 2 times higher likelihood to H0
than H1.

## Craving Intensity for Decrease Foods

## Bias for Decrease Foods

## Craving Intensity for Decrease Foods moderated by restrained eating

## Craving Intensity for Decrease Foods moderated by perceived self-regulatory success

# Moderation Analyses

As pre-registered, we calculated interaction models with different
participant-level, stimulus-level and training-level variables for the
changes from pre- to post training. We added each moderator with an
interaction to the models with all three outcome variables (approach
bias, craving and intake). In all moderator multilevel models a random
slope for time as well as a random intercept per participant and
stimulus was included. For craving and intake, as we used hurdle-gamma
models for the model prediction, in the model-based plots for the hurdle
part of the model (“hu”) shown here a higher value stands for a higher
probability of no craving/intake. The model-based plots where the
outcome variable is indicated on the Y-axis are the results of the gamma
part of the model predicting the intensity of craving/amount of intake
when craving/intake occurred. An overview of effects can be found in the
table below.

Note: The results depicted in this table are the results of the
three-way interaction Group × Time × moderator of models examining the
effects from pre- to post-intervention assessments. The values are the
model estimates, the color indicates the probability of direction, i.e.,
the proportion of the posterior distribution that is either above or
below 0 (whichever is more probable). The lines around the table fields
indicate, whether the 89% or the 95% HDI (highest probability interval
of the posterior distribution) are not including 0. Our computing power
was not high enough to compute the models predicting Intake and Craving
with a moderation by Intention, the models predicting Craving by
Pre-training Craving as well as the models predicting approach bias by
some of the UPPS subscales.

## Participant-level moderators regarding traits

On a participant-level, we examined perceived self-regulatory success
(PSRS scores), restrained eating (DEBQres) and Impulsivity (UPPS) as
potential moderators. While the methods and the model results and plots
for the moderation of craving intensity for decrease foods by PSRS and
DEBQres are included in the paper, all other results are included here.
They are, for each category and moderator, presented in the order:
Approach bias, Craving, Intake. Regarding the UPPS, some of the models
with approach bias as the outcome variable could not be computed with
the given computing power.

### Perceived Self-regulatory success (PSRS scores)

**Decrease foods:**

```
## Approach Bias
```

```
## bias_stim ~ is_post * is_active_training * PSRS + (is_post | vpNumber) + (is_post | imgNumber)
```

```
##                                      Estimate Est.Error       Q2.5     Q97.5
## Intercept                           67.577452  43.15870  -19.14784 153.68530
## is_post1                           -12.112489  48.80193 -114.08429  76.72603
## is_active_training1                -10.061973  64.44527 -140.22774 113.89228
## PSRS                                -4.212876  11.60415  -26.97640  19.21182
## is_post1:is_active_training1      -128.684318  74.44801 -271.84219  20.56348
## is_post1:PSRS                        3.374326  12.94230  -21.00307  29.58061
## is_active_training1:PSRS             6.214555  17.08758  -27.14062  41.07503
## is_post1:is_active_training1:PSRS   24.987718  19.85459  -14.88751  64.06192
```

```
## Craving
```

```
## Craving ~ is_post * is_active_training * PSRS + (is_post | vpNumber) + (is_post | imgNumber) 
## hu ~ is_post * is_active_training * PSRS + (is_post | vpNumber) + (is_post | imgNumber)
```

```
##                                          Estimate  Est.Error        Q2.5
## Intercept                             3.745165825 0.13015240  3.48415071
## hu_Intercept                         -1.882745285 0.53075746 -2.89354548
## is_post1                             -0.001048752 0.13319608 -0.26728357
## is_active_training1                   0.215327706 0.20133206 -0.18580966
## PSRS                                  0.009732594 0.03515861 -0.05994687
## is_post1:is_active_training1         -0.646578812 0.20718766 -1.03941118
## is_post1:PSRS                        -0.028670707 0.03514157 -0.09608294
## is_active_training1:PSRS             -0.072670284 0.05393264 -0.18300041
## is_post1:is_active_training1:PSRS     0.128478430 0.05527167  0.02144002
## hu_is_post1                           0.597564017 0.50466152 -0.40220412
## hu_is_active_training1                1.130802720 0.78878981 -0.43646274
## hu_PSRS                               0.184133445 0.13855212 -0.08140217
## hu_is_post1:is_active_training1       0.193076579 0.74201291 -1.21554507
## hu_is_post1:PSRS                     -0.041259910 0.13268671 -0.29871476
## hu_is_active_training1:PSRS          -0.246986063 0.21396029 -0.65325758
## hu_is_post1:is_active_training1:PSRS -0.129862778 0.20037588 -0.52386916
##                                            Q97.5
## Intercept                             3.99771965
## hu_Intercept                         -0.85333902
## is_post1                              0.25113666
## is_active_training1                   0.61843577
## PSRS                                  0.08006651
## is_post1:is_active_training1         -0.24124600
## is_post1:PSRS                         0.04128068
## is_active_training1:PSRS              0.03366296
## is_post1:is_active_training1:PSRS     0.23517322
## hu_is_post1                           1.57907229
## hu_is_active_training1                2.67016287
## hu_PSRS                               0.44906255
## hu_is_post1:is_active_training1       1.67264704
## hu_is_post1:PSRS                      0.22064699
## hu_is_active_training1:PSRS           0.17865221
## hu_is_post1:is_active_training1:PSRS  0.25816251
```

```
## Intake
```

```
## Intake ~ is_post * is_active_training * PSRS + (is_post | vpNumber) + (is_post | imgNumber) 
## hu ~ is_post * is_active_training * PSRS + (is_post | vpNumber) + (is_post | imgNumber)
```

```
##                                         Estimate  Est.Error        Q2.5
## Intercept                             3.48697438 0.16048419  3.18252325
## hu_Intercept                         -0.12829190 0.35787204 -0.85138567
## is_post1                             -0.23468785 0.18399844 -0.61377176
## is_active_training1                   0.19827953 0.24580575 -0.28174646
## PSRS                                  0.03522065 0.04233787 -0.04772688
## is_post1:is_active_training1          0.02165999 0.27148985 -0.52911789
## is_post1:PSRS                         0.02711541 0.04886065 -0.06889055
## is_active_training1:PSRS             -0.06442561 0.06592477 -0.19536210
## is_post1:is_active_training1:PSRS    -0.01798522 0.07253823 -0.16047602
## hu_is_post1                           0.29298739 0.37258762 -0.43070418
## hu_is_active_training1                0.14909004 0.51346815 -0.82643764
## hu_PSRS                               0.09441007 0.09175742 -0.08838073
## hu_is_post1:is_active_training1       0.14953339 0.55782747 -0.95123024
## hu_is_post1:PSRS                     -0.04930367 0.09842028 -0.24281584
## hu_is_active_training1:PSRS          -0.02264375 0.13521830 -0.29924762
## hu_is_post1:is_active_training1:PSRS -0.06949385 0.14887319 -0.35676080
##                                          Q97.5
## Intercept                            3.7934815
## hu_Intercept                         0.5629871
## is_post1                             0.1225083
## is_active_training1                  0.6827885
## PSRS                                 0.1159690
## is_post1:is_active_training1         0.5622155
## is_post1:PSRS                        0.1258787
## is_active_training1:PSRS             0.0656157
## is_post1:is_active_training1:PSRS    0.1268445
## hu_is_post1                          1.0419335
## hu_is_active_training1               1.1774605
## hu_PSRS                              0.2769182
## hu_is_post1:is_active_training1      1.2159075
## hu_is_post1:PSRS                     0.1437155
## hu_is_active_training1:PSRS          0.2286333
## hu_is_post1:is_active_training1:PSRS 0.2249006
```

**Increase foods:**

```
## Approach Bias
```

```
## bias_stim ~ is_post * is_active_training * PSRS + (is_post | vpNumber) + (is_post | imgNumber)
```

```
##                                     Estimate Est.Error        Q2.5     Q97.5
## Intercept                         106.256031  49.97675    8.340778 207.53525
## is_post1                          -36.359130  62.56281 -162.444286  87.60971
## is_active_training1               -12.364977  70.34013 -153.309673 123.34494
## PSRS                              -13.684098  13.33077  -40.189312  13.27827
## is_post1:is_active_training1      -54.495898  89.56196 -228.973554 121.47525
## is_post1:PSRS                       8.465390  16.62236  -24.143524  42.11336
## is_active_training1:PSRS            6.853042  18.89257  -30.337929  44.84191
## is_post1:is_active_training1:PSRS  10.875488  24.03757  -37.405349  58.15775
```

```
## Craving
```

```
## Craving ~ is_post * is_active_training * PSRS + (is_post | vpNumber) + (is_post | imgNumber) 
## hu ~ is_post * is_active_training * PSRS + (is_post | vpNumber) + (is_post | imgNumber)
```

```
##                                          Estimate  Est.Error        Q2.5
## Intercept                             3.581977549 0.14917332  3.29398734
## hu_Intercept                         -1.099985436 0.54085779 -2.13607785
## is_post1                             -0.198706917 0.18233033 -0.54770025
## is_active_training1                   0.143914090 0.21999203 -0.27961751
## PSRS                                  0.012704175 0.03987824 -0.06633276
## is_post1:is_active_training1         -0.053478683 0.27973276 -0.60802721
## is_post1:PSRS                         0.016513921 0.04899318 -0.08172479
## is_active_training1:PSRS             -0.041496753 0.05899394 -0.15713440
## is_post1:is_active_training1:PSRS     0.002951515 0.07356956 -0.13833655
## hu_is_post1                          -0.101726497 0.53008805 -1.12288153
## hu_is_active_training1                2.070043428 0.82591376  0.45057842
## hu_PSRS                               0.117748672 0.14362741 -0.17141014
## hu_is_post1:is_active_training1       0.841998233 0.79725559 -0.74727222
## hu_is_post1:PSRS                      0.072025956 0.13694827 -0.19677546
## hu_is_active_training1:PSRS          -0.501968809 0.22066741 -0.93221260
## hu_is_post1:is_active_training1:PSRS -0.329876168 0.21239092 -0.74776620
##                                            Q97.5
## Intercept                             3.87395899
## hu_Intercept                         -0.05632289
## is_post1                              0.16547246
## is_active_training1                   0.57977058
## PSRS                                  0.08968547
## is_post1:is_active_training1          0.48872922
## is_post1:PSRS                         0.11003975
## is_active_training1:PSRS              0.07357399
## is_post1:is_active_training1:PSRS     0.15076590
## hu_is_post1                           0.93863479
## hu_is_active_training1                3.68144627
## hu_PSRS                               0.38697883
## hu_is_post1:is_active_training1       2.40490272
## hu_is_post1:PSRS                      0.34434204
## hu_is_active_training1:PSRS          -0.06846942
## hu_is_post1:is_active_training1:PSRS  0.08994989
```

```
## Intake
```

```
## Intake ~ is_post * is_active_training * PSRS + (is_post | vpNumber) + (is_post | imgNumber) 
## hu ~ is_post * is_active_training * PSRS + (is_post | vpNumber) + (is_post | imgNumber)
```

```
##                                          Estimate  Est.Error        Q2.5
## Intercept                             3.549927290 0.15976995  3.23514412
## hu_Intercept                          0.288721884 0.45591623 -0.59542842
## is_post1                             -0.355180210 0.22679486 -0.80507625
## is_active_training1                   0.026111480 0.23330580 -0.43090334
## PSRS                                 -0.008208638 0.04203355 -0.09134892
## is_post1:is_active_training1         -0.143374886 0.34374275 -0.81669751
## is_post1:PSRS                         0.054190557 0.06049564 -0.06398499
## is_active_training1:PSRS              0.025323188 0.06252002 -0.09571240
## is_post1:is_active_training1:PSRS     0.006553240 0.09152990 -0.17422526
## hu_is_post1                          -0.201879925 0.39781291 -0.99061152
## hu_is_active_training1                0.982978575 0.68031187 -0.37668291
## hu_PSRS                               0.098911976 0.12124016 -0.14013172
## hu_is_post1:is_active_training1       0.331852440 0.59769779 -0.84423510
## hu_is_post1:PSRS                      0.049741445 0.10434493 -0.15518250
## hu_is_active_training1:PSRS          -0.261055221 0.18399175 -0.61651255
## hu_is_post1:is_active_training1:PSRS -0.153908394 0.15907708 -0.46211816
##                                           Q97.5
## Intercept                            3.86702058
## hu_Intercept                         1.18705866
## is_post1                             0.08530767
## is_active_training1                  0.47820636
## PSRS                                 0.07433870
## is_post1:is_active_training1         0.52472392
## is_post1:PSRS                        0.17335435
## is_active_training1:PSRS             0.14910515
## is_post1:is_active_training1:PSRS    0.18504398
## hu_is_post1                          0.57932242
## hu_is_active_training1               2.31777629
## hu_PSRS                              0.33237774
## hu_is_post1:is_active_training1      1.49755103
## hu_is_post1:PSRS                     0.25376920
## hu_is_active_training1:PSRS          0.10610021
## hu_is_post1:is_active_training1:PSRS 0.15945993
```

### Restrained Eating (DEBQres scores)

**Decrease foods:**

```
## Approach Bias
```

```
## bias_stim ~ is_post * is_active_training * DEBQres + (is_post | vpNumber) + (is_post | imgNumber)
```

```
##                                        Estimate Est.Error        Q2.5
## Intercept                             -6.692706  47.28352  -98.797369
## is_post1                              48.558100  57.98084  -64.036553
## is_active_training1                  116.180107  65.44620  -12.797567
## DEBQres                               21.840057  16.96246   -9.771016
## is_post1:is_active_training1         -69.160960  79.27356 -229.481197
## is_post1:DEBQres                     -18.014628  20.88401  -59.304880
## is_active_training1:DEBQres          -42.419396  25.94468  -93.423159
## is_post1:is_active_training1:DEBQres  10.574903  31.12022  -51.787069
##                                           Q97.5
## Intercept                             84.169983
## is_post1                             168.666500
## is_active_training1                  242.421210
## DEBQres                               55.078512
## is_post1:is_active_training1          90.560765
## is_post1:DEBQres                      23.025617
## is_active_training1:DEBQres            6.429159
## is_post1:is_active_training1:DEBQres  71.513450
```

```
## Craving
```

```
## Craving ~ is_post * is_active_training * DEBQres + (is_post | vpNumber) + (is_post | imgNumber) 
## hu ~ is_post * is_active_training * DEBQres + (is_post | vpNumber) + (is_post | imgNumber)
```

```
##                                             Estimate  Est.Error       Q2.5
## Intercept                                3.835032208 0.15457234  3.5220132
## hu_Intercept                            -1.584934109 0.60577975 -2.7835413
## is_post1                                -0.120456702 0.16004491 -0.4471759
## is_active_training1                     -0.123217258 0.21156038 -0.5314096
## DEBQres                                 -0.021088577 0.05695253 -0.1351117
## is_post1:is_active_training1             0.246871937 0.21815887 -0.1724779
## is_post1:DEBQres                         0.007132344 0.05895310 -0.1071171
## is_active_training1:DEBQres              0.031470126 0.08263639 -0.1341501
## is_post1:is_active_training1:DEBQres    -0.187408920 0.08502387 -0.3572269
## hu_is_post1                              0.952734250 0.56305921 -0.1184213
## hu_is_active_training1                   1.222734278 0.84169579 -0.3910146
## hu_DEBQres                               0.137978527 0.22138708 -0.2875550
## hu_is_post1:is_active_training1         -1.248720891 0.78940399 -2.7839317
## hu_is_post1:DEBQres                     -0.194323370 0.20546987 -0.5993574
## hu_is_active_training1:DEBQres          -0.400581125 0.32964053 -1.0760005
## hu_is_post1:is_active_training1:DEBQres  0.405839281 0.30749672 -0.1933268
##                                               Q97.5
## Intercept                                4.15332228
## hu_Intercept                            -0.42979609
## is_post1                                 0.19096725
## is_active_training1                      0.30377783
## DEBQres                                  0.09239358
## is_post1:is_active_training1             0.68464814
## is_post1:DEBQres                         0.12903485
## is_active_training1:DEBQres              0.19191197
## is_post1:is_active_training1:DEBQres    -0.02431416
## hu_is_post1                              2.05000407
## hu_is_active_training1                   2.95983270
## hu_DEBQres                               0.57497183
## hu_is_post1:is_active_training1          0.26726855
## hu_is_post1:DEBQres                      0.19524357
## hu_is_active_training1:DEBQres           0.23417824
## hu_is_post1:is_active_training1:DEBQres  0.99090923
```

```
## Intake
```

```
## Intake ~ is_post * is_active_training * DEBQres + (is_post | vpNumber) + (is_post | imgNumber) 
## hu ~ is_post * is_active_training * DEBQres + (is_post | vpNumber) + (is_post | imgNumber)
```

```
##                                            Estimate  Est.Error       Q2.5
## Intercept                                3.88398584 0.18582526  3.5284598
## hu_Intercept                             0.38384828 0.42013060 -0.4222761
## is_post1                                -0.06092203 0.21477408 -0.4768242
## is_active_training1                     -0.26917299 0.26172414 -0.7792087
## DEBQres                                 -0.10368683 0.06769155 -0.2376227
## is_post1:is_active_training1             0.18325934 0.28789142 -0.3884062
## is_post1:DEBQres                        -0.02940075 0.07865918 -0.1838934
## is_active_training1:DEBQres              0.08958890 0.10197715 -0.1130745
## is_post1:is_active_training1:DEBQres    -0.10300887 0.11180556 -0.3210707
## hu_is_post1                              0.56426125 0.44199930 -0.3190117
## hu_is_active_training1                  -0.08499117 0.57413069 -1.1982418
## hu_DEBQres                              -0.06866885 0.14914200 -0.3655582
## hu_is_post1:is_active_training1         -0.72379141 0.62077905 -1.9136898
## hu_is_post1:DEBQres                     -0.17338039 0.16183948 -0.4879681
## hu_is_active_training1:DEBQres           0.05774570 0.22403930 -0.3957300
## hu_is_post1:is_active_training1:DEBQres  0.25239295 0.24288281 -0.2280275
##                                              Q97.5
## Intercept                               4.25201358
## hu_Intercept                            1.22794635
## is_post1                                0.36021967
## is_active_training1                     0.25061747
## DEBQres                                 0.02883574
## is_post1:is_active_training1            0.75994255
## is_post1:DEBQres                        0.12321993
## is_active_training1:DEBQres             0.29222504
## is_post1:is_active_training1:DEBQres    0.11926376
## hu_is_post1                             1.40479216
## hu_is_active_training1                  1.06512389
## hu_DEBQres                              0.21988203
## hu_is_post1:is_active_training1         0.48884672
## hu_is_post1:DEBQres                     0.15092693
## hu_is_active_training1:DEBQres          0.49787215
## hu_is_post1:is_active_training1:DEBQres 0.72550824
```

**Increase foods:**

```
## Approach Bias
```

```
## bias_stim ~ is_post * is_active_training * DEBQres + (is_post | vpNumber) + (is_post | imgNumber)
```

```
##                                         Estimate Est.Error       Q2.5     Q97.5
## Intercept                              -7.418515  53.45364 -110.96464  99.63431
## is_post1                               87.157123  66.91924  -44.15357 220.66724
## is_active_training1                    73.732635  77.43124  -75.86265 230.68438
## DEBQres                                24.856432  19.40547  -12.89819  62.23495
## is_post1:is_active_training1         -115.465352  96.69252 -300.06399  75.07673
## is_post1:DEBQres                      -35.458030  24.34280  -82.31235  11.95098
## is_active_training1:DEBQres           -23.966080  30.78115  -85.71190  34.76274
## is_post1:is_active_training1:DEBQres   38.959565  38.93559  -35.28284 113.05988
```

```
## Craving
```

```
## Craving ~ is_post * is_active_training * DEBQres + (is_post | vpNumber) + (is_post | imgNumber) 
## hu ~ is_post * is_active_training * DEBQres + (is_post | vpNumber) + (is_post | imgNumber)
```

```
##                                             Estimate  Est.Error       Q2.5
## Intercept                                3.582911307 0.17627244  3.2240124
## hu_Intercept                            -0.959331928 0.66508212 -2.2824411
## is_post1                                 0.008512985 0.21495062 -0.4087665
## is_active_training1                      0.268510086 0.24548805 -0.2072768
## DEBQres                                  0.017642934 0.06583242 -0.1094707
## is_post1:is_active_training1            -0.046067960 0.30171228 -0.6445383
## is_post1:DEBQres                        -0.057522748 0.08019526 -0.2122381
## is_active_training1:DEBQres             -0.115463171 0.09636290 -0.3010001
## is_post1:is_active_training1:DEBQres    -0.007057853 0.11910783 -0.2442840
## hu_is_post1                              0.105617090 0.56670923 -1.0105566
## hu_is_active_training1                   0.743754134 0.90499934 -1.0850091
## hu_DEBQres                               0.108366016 0.24276222 -0.3603919
## hu_is_post1:is_active_training1         -1.522656137 0.80846835 -3.1366755
## hu_is_post1:DEBQres                      0.019811921 0.20530726 -0.3911368
## hu_is_active_training1:DEBQres          -0.190762633 0.35522635 -0.8652763
## hu_is_post1:is_active_training1:DEBQres  0.524756233 0.31916647 -0.0754338
##                                              Q97.5
## Intercept                               3.93804647
## hu_Intercept                            0.30876161
## is_post1                                0.42854658
## is_active_training1                     0.75120993
## DEBQres                                 0.14783371
## is_post1:is_active_training1            0.54930584
## is_post1:DEBQres                        0.10255161
## is_active_training1:DEBQres             0.07262939
## is_post1:is_active_training1:DEBQres    0.23010437
## hu_is_post1                             1.22207840
## hu_is_active_training1                  2.54143057
## hu_DEBQres                              0.58282022
## hu_is_post1:is_active_training1         0.04798746
## hu_is_post1:DEBQres                     0.42060761
## hu_is_active_training1:DEBQres          0.52354782
## hu_is_post1:is_active_training1:DEBQres 1.16046753
```

```
## Intake
```

```
## Intake ~ is_post * is_active_training * DEBQres + (is_post | vpNumber) + (is_post | imgNumber) 
## hu ~ is_post * is_active_training * DEBQres + (is_post | vpNumber) + (is_post | imgNumber)
```

```
##                                            Estimate  Est.Error       Q2.5
## Intercept                                3.50154903 0.18773704  3.1200290
## hu_Intercept                             1.38137502 0.55022536  0.2885560
## is_post1                                 0.14053819 0.28047560 -0.4021849
## is_active_training1                      0.24233635 0.24620586 -0.2418848
## DEBQres                                  0.00854816 0.06818398 -0.1236523
## is_post1:is_active_training1            -0.23226725 0.35943891 -0.9365271
## is_post1:DEBQres                        -0.11626867 0.10206737 -0.3108539
## is_active_training1:DEBQres             -0.05434262 0.09573616 -0.2405657
## is_post1:is_active_training1:DEBQres     0.03225262 0.14083384 -0.2332611
## hu_is_post1                             -0.06729846 0.47217112 -0.9921229
## hu_is_active_training1                  -0.96122454 0.75910140 -2.4007366
## hu_DEBQres                              -0.28034281 0.20081056 -0.6708611
## hu_is_post1:is_active_training1         -0.87626759 0.62963674 -2.0938465
## hu_is_post1:DEBQres                      0.01762452 0.17115143 -0.3179721
## hu_is_active_training1:DEBQres           0.40400736 0.29388113 -0.1578195
## hu_is_post1:is_active_training1:DEBQres  0.29084678 0.24710846 -0.1997384
##                                              Q97.5
## Intercept                               3.86566253
## hu_Intercept                            2.45723025
## is_post1                                0.68168100
## is_active_training1                     0.71965667
## DEBQres                                 0.14676758
## is_post1:is_active_training1            0.46048817
## is_post1:DEBQres                        0.08998172
## is_active_training1:DEBQres             0.13267792
## is_post1:is_active_training1:DEBQres    0.30696481
## hu_is_post1                             0.86159708
## hu_is_active_training1                  0.56649504
## hu_DEBQres                              0.12717060
## hu_is_post1:is_active_training1         0.36353154
## hu_is_post1:DEBQres                     0.34822034
## hu_is_active_training1:DEBQres          0.96505491
## hu_is_post1:is_active_training1:DEBQres 0.77629405
```

### Impulsivity (UPPS scores)

**Decrease foods:**

```
## Approach Bias - Subscale: UPPS Perseverance
```

```
## bias_stim ~ is_post * is_active_training * UPPSpers + (is_post | vpNumber) + (is_post | imgNumber)
```

```
##                                         Estimate Est.Error       Q2.5     Q97.5
## Intercept                             102.716860  66.63152  -29.12199 231.99271
## is_post1                              -86.477495  76.15026 -236.66743  62.05601
## is_active_training1                    86.994144 105.11267 -119.51340 293.62604
## UPPSpers                              -17.577112  22.32069  -60.87773  26.05963
## is_post1:is_active_training1           -9.728344 116.65378 -234.84010 223.48000
## is_post1:UPPSpers                      29.867300  25.41786  -20.39426  79.86345
## is_active_training1:UPPSpers          -24.201968  34.67435  -93.65219  44.20490
## is_post1:is_active_training1:UPPSpers -10.578940  38.28931  -86.10947  64.13056
```

```
## Approach Bias - Subscale: UPPS Premeditation
```

```
## bias_stim ~ is_post * is_active_training * UPPSprem + (is_post | vpNumber) + (is_post | imgNumber)
```

```
##                                        Estimate Est.Error       Q2.5      Q97.5
## Intercept                              19.54686  80.61389 -134.06275 187.253545
## is_post1                              -66.35195  90.92301 -244.89553 109.309193
## is_active_training1                   218.36699 117.98370  -14.89919 451.430823
## UPPSprem                               10.70901  26.41647  -43.16220  61.253511
## is_post1:is_active_training1          -84.32448 132.55677 -340.38876 179.744851
## is_post1:UPPSprem                      22.50167  30.10733  -36.52645  80.470564
## is_active_training1:UPPSprem          -68.52304  38.32593 -143.85461   7.908344
## is_post1:is_active_training1:UPPSprem  15.20035  43.36324  -71.00754  99.333051
```

```
## Craving - Subscale: UPPS Perseverance
```

```
## Craving ~ is_post * is_active_training * UPPSpers + (is_post | vpNumber) + (is_post | imgNumber) 
## hu ~ is_post * is_active_training * UPPSpers + (is_post | vpNumber) + (is_post | imgNumber)
```

```
##                                              Estimate  Est.Error        Q2.5
## Intercept                                 4.063066540 0.19531802  3.67323320
## hu_Intercept                             -2.990118815 0.78899933 -4.54765110
## is_post1                                 -0.259525831 0.21695625 -0.68563661
## is_active_training1                      -0.354234003 0.31776119 -0.97788900
## UPPSpers                                 -0.098270714 0.06600833 -0.22806558
## is_post1:is_active_training1             -0.186946072 0.34617945 -0.85335169
## is_post1:UPPSpers                         0.055106541 0.07307557 -0.08818380
## is_active_training1:UPPSpers              0.106942172 0.10457127 -0.09598554
## is_post1:is_active_training1:UPPSpers    -0.003941258 0.11339184 -0.22432309
## hu_is_post1                               1.572528652 0.78055601  0.04711448
## hu_is_active_training1                    0.591430744 1.21075100 -1.76939471
## hu_UPPSpers                               0.607374043 0.26326415  0.08205192
## hu_is_post1:is_active_training1          -0.584408464 1.20587946 -3.01245930
## hu_is_post1:UPPSpers                     -0.382945109 0.25701568 -0.88935193
## hu_is_active_training1:UPPSpers          -0.136265489 0.39321802 -0.90996724
## hu_is_post1:is_active_training1:UPPSpers  0.120642929 0.39110757 -0.62904414
##                                                Q97.5
## Intercept                                 4.44627533
## hu_Intercept                             -1.41667976
## is_post1                                  0.16569662
## is_active_training1                       0.25594393
## UPPSpers                                  0.03023562
## is_post1:is_active_training1              0.48020214
## is_post1:UPPSpers                         0.19874959
## is_active_training1:UPPSpers              0.30515072
## is_post1:is_active_training1:UPPSpers     0.21518448
## hu_is_post1                               3.09769101
## hu_is_active_training1                    2.99478193
## hu_UPPSpers                               1.12278551
## hu_is_post1:is_active_training1           1.77794033
## hu_is_post1:UPPSpers                      0.12565140
## hu_is_active_training1:UPPSpers           0.62067795
## hu_is_post1:is_active_training1:UPPSpers  0.91099901
```

```
## Craving - Subscale: UPPS Premeditation
```

```
## Craving ~ is_post * is_active_training * UPPSprem + (is_post | vpNumber) + (is_post | imgNumber) 
## hu ~ is_post * is_active_training * UPPSprem + (is_post | vpNumber) + (is_post | imgNumber)
```

```
##                                              Estimate  Est.Error        Q2.5
## Intercept                                 4.203885743 0.23499875  3.73292357
## hu_Intercept                             -1.537942948 0.98273978 -3.42733762
## is_post1                                 -0.382178845 0.27189244 -0.91459572
## is_active_training1                      -0.794112502 0.35621132 -1.48796924
## UPPSprem                                 -0.140784499 0.07649142 -0.28501473
## is_post1:is_active_training1             -0.002853898 0.39969015 -0.77676153
## is_post1:UPPSprem                         0.093223263 0.08855689 -0.07761989
## is_active_training1:UPPSprem              0.248709850 0.11503392  0.01745203
## is_post1:is_active_training1:UPPSprem    -0.062709358 0.12969798 -0.31745516
## hu_is_post1                               1.774461346 0.88239882  0.06982622
## hu_is_active_training1                    1.354274415 1.43104840 -1.55228975
## hu_UPPSprem                               0.099530956 0.32202876 -0.54528580
## hu_is_post1:is_active_training1          -1.566783973 1.29848080 -4.10183325
## hu_is_post1:UPPSprem                     -0.436118277 0.28628667 -0.98499476
## hu_is_active_training1:UPPSprem          -0.360281601 0.46851459 -1.27387654
## hu_is_post1:is_active_training1:UPPSprem  0.430644355 0.41884239 -0.35680571
##                                                Q97.5
## Intercept                                 4.64213554
## hu_Intercept                              0.44676370
## is_post1                                  0.14164989
## is_active_training1                      -0.08289567
## UPPSprem                                  0.01085771
## is_post1:is_active_training1              0.76707875
## is_post1:UPPSprem                         0.26363065
## is_active_training1:UPPSprem              0.47376240
## is_post1:is_active_training1:UPPSprem     0.19073162
## hu_is_post1                               3.46922840
## hu_is_active_training1                    4.14509762
## hu_UPPSprem                               0.71385216
## hu_is_post1:is_active_training1           0.87948449
## hu_is_post1:UPPSprem                      0.12243264
## hu_is_active_training1:UPPSprem           0.58662888
## hu_is_post1:is_active_training1:UPPSprem  1.23650376
```

```
## Craving - Subscale: UPPS Sensation Seeking
```

```
## Craving ~ is_post * is_active_training * UPPSsen + (is_post | vpNumber) + (is_post | imgNumber) 
## hu ~ is_post * is_active_training * UPPSsen + (is_post | vpNumber) + (is_post | imgNumber)
```

```
##                                            Estimate  Est.Error        Q2.5
## Intercept                                3.64284901 0.14311859  3.35848019
## hu_Intercept                            -1.15649074 0.58997036 -2.32089468
## is_post1                                -0.12834642 0.15062421 -0.42387088
## is_active_training1                      0.09047050 0.23101778 -0.34628601
## UPPSsen                                  0.05851834 0.05830204 -0.05878349
## is_post1:is_active_training1            -0.24820167 0.24786707 -0.73172492
## is_post1:UPPSsen                         0.01084834 0.06109015 -0.10874696
## is_active_training1:UPPSsen             -0.05678961 0.09046195 -0.23715241
## is_post1:is_active_training1:UPPSsen     0.02306896 0.09725791 -0.16645675
## hu_is_post1                              0.52079733 0.56012306 -0.56221543
## hu_is_active_training1                   1.30079009 0.95064026 -0.54399775
## hu_UPPSsen                              -0.02649196 0.23957083 -0.49330911
## hu_is_post1:is_active_training1         -1.20155933 0.89877749 -2.97606178
## hu_is_post1:UPPSsen                     -0.03083392 0.22756350 -0.47932340
## hu_is_active_training1:UPPSsen          -0.41301945 0.37353277 -1.14367011
## hu_is_post1:is_active_training1:UPPSsen  0.37646889 0.35575813 -0.33639899
##                                               Q97.5
## Intercept                                3.92813716
## hu_Intercept                            -0.01118785
## is_post1                                 0.17479085
## is_active_training1                      0.55062856
## UPPSsen                                  0.17324168
## is_post1:is_active_training1             0.22884078
## is_post1:UPPSsen                         0.12932185
## is_active_training1:UPPSsen              0.11376642
## is_post1:is_active_training1:UPPSsen     0.21678457
## hu_is_post1                              1.61224858
## hu_is_active_training1                   3.14322154
## hu_UPPSsen                               0.44576255
## hu_is_post1:is_active_training1          0.58124944
## hu_is_post1:UPPSsen                      0.41297299
## hu_is_active_training1:UPPSsen           0.31427762
## hu_is_post1:is_active_training1:UPPSsen  1.07804445
```

```
## Craving - Subscale: UPPS Urgency
```

```
## Craving ~ is_post * is_active_training * UPPSurg + (is_post | vpNumber) + (is_post | imgNumber) 
## hu ~ is_post * is_active_training * UPPSurg + (is_post | vpNumber) + (is_post | imgNumber)
```

```
##                                             Estimate  Est.Error       Q2.5
## Intercept                                3.675315921 0.18665288  3.3010750
## hu_Intercept                            -0.128896001 0.75299314 -1.5680826
## is_post1                                -0.103158445 0.20466378 -0.5203822
## is_active_training1                      0.153235674 0.26441141 -0.3748523
## UPPSurg                                  0.046926316 0.07947785 -0.1062938
## is_post1:is_active_training1            -0.172923953 0.28057472 -0.7150684
## is_post1:UPPSurg                        -0.000020869 0.08720258 -0.1683477
## is_active_training1:UPPSurg             -0.089064177 0.11448978 -0.3229002
## is_post1:is_active_training1:UPPSurg    -0.006488265 0.12101913 -0.2413747
## hu_is_post1                              0.417216712 0.72260649 -0.9683696
## hu_is_active_training1                  -0.028255480 1.01815671 -2.0419009
## hu_UPPSurg                              -0.481435721 0.32296135 -1.1249325
## hu_is_post1:is_active_training1         -1.042183708 1.01519520 -3.0164563
## hu_is_post1:UPPSurg                      0.013323877 0.31341085 -0.5942275
## hu_is_active_training1:UPPSurg           0.114671560 0.44744529 -0.7563299
## hu_is_post1:is_active_training1:UPPSurg  0.358200330 0.44461505 -0.5041616
##                                             Q97.5
## Intercept                               4.0469977
## hu_Intercept                            1.3374621
## is_post1                                0.2885088
## is_active_training1                     0.6870966
## UPPSurg                                 0.2070812
## is_post1:is_active_training1            0.3828808
## is_post1:UPPSurg                        0.1741246
## is_active_training1:UPPSurg             0.1416298
## is_post1:is_active_training1:UPPSurg    0.2244809
## hu_is_post1                             1.8284260
## hu_is_active_training1                  1.9777931
## hu_UPPSurg                              0.1473159
## hu_is_post1:is_active_training1         0.8896659
## hu_is_post1:UPPSurg                     0.6233080
## hu_is_active_training1:UPPSurg          1.0002251
## hu_is_post1:is_active_training1:UPPSurg 1.2430550
```

```
## Intake - Subscale: UPPS Perseverance
```

```
## Intake ~ is_post * is_active_training * UPPSpers + (is_post | vpNumber) + (is_post | imgNumber) 
## hu ~ is_post * is_active_training * UPPSpers + (is_post | vpNumber) + (is_post | imgNumber)
```

```
##                                              Estimate  Est.Error       Q2.5
## Intercept                                 4.146829981 0.23286376  3.6914935
## hu_Intercept                             -0.462483814 0.54534816 -1.5509049
## is_post1                                 -0.266518463 0.27379631 -0.8134131
## is_active_training1                       0.032804713 0.36220324 -0.6821820
## UPPSpers                                 -0.186231499 0.07780706 -0.3381770
## is_post1:is_active_training1             -0.452646889 0.43882906 -1.3132751
## is_post1:UPPSpers                         0.045634946 0.09194733 -0.1318518
## is_active_training1:UPPSpers             -0.009326027 0.11809609 -0.2344121
## is_post1:is_active_training1:UPPSpers     0.130317546 0.14303116 -0.1597655
## hu_is_post1                               0.976200439 0.60590328 -0.2148468
## hu_is_active_training1                   -0.023472143 0.85109265 -1.7206697
## hu_UPPSpers                               0.227553419 0.17990882 -0.1347344
## hu_is_post1:is_active_training1          -0.335173909 0.93263086 -2.1564323
## hu_is_post1:UPPSpers                     -0.294776288 0.20264918 -0.6802585
## hu_is_active_training1:UPPSpers           0.016553405 0.27892785 -0.5234055
## hu_is_post1:is_active_training1:UPPSpers  0.094071488 0.30499362 -0.5062061
##                                                Q97.5
## Intercept                                 4.60190771
## hu_Intercept                              0.63722409
## is_post1                                  0.26118750
## is_active_training1                       0.72359195
## UPPSpers                                 -0.03382661
## is_post1:is_active_training1              0.44655560
## is_post1:UPPSpers                         0.23130623
## is_active_training1:UPPSpers              0.22408968
## is_post1:is_active_training1:UPPSpers     0.41219075
## hu_is_post1                               2.13422641
## hu_is_active_training1                    1.61637106
## hu_UPPSpers                               0.58013034
## hu_is_post1:is_active_training1           1.50242636
## hu_is_post1:UPPSpers                      0.10506673
## hu_is_active_training1:UPPSpers           0.57294049
## hu_is_post1:is_active_training1:UPPSpers  0.68507387
```

```
## Intake - Subscale: UPPS Premeditation
```

```
## Intake ~ is_post * is_active_training * UPPSprem + (is_post | vpNumber) + (is_post | imgNumber) 
## hu ~ is_post * is_active_training * UPPSprem + (is_post | vpNumber) + (is_post | imgNumber)
```

```
##                                             Estimate  Est.Error        Q2.5
## Intercept                                 4.17852353 0.28280544  3.62321863
## hu_Intercept                             -0.16064141 0.63719104 -1.39180273
## is_post1                                  0.01032602 0.34841628 -0.69183642
## is_active_training1                      -0.79596316 0.42182759 -1.61798824
## UPPSprem                                 -0.19015479 0.09311912 -0.37452019
## is_post1:is_active_training1              0.11274285 0.50450379 -0.87070046
## is_post1:UPPSprem                        -0.04804981 0.11314092 -0.26892550
## is_active_training1:UPPSprem              0.25573142 0.13764084 -0.01193618
## is_post1:is_active_training1:UPPSprem    -0.05115553 0.16421439 -0.37616122
## hu_is_post1                               1.28860290 0.72686562 -0.15450094
## hu_is_active_training1                    0.48828609 0.92494677 -1.29469319
## hu_UPPSprem                               0.12040844 0.20918798 -0.29703008
## hu_is_post1:is_active_training1          -2.25934962 1.06000959 -4.36669398
## hu_is_post1:UPPSprem                     -0.38906157 0.23813808 -0.86243763
## hu_is_active_training1:UPPSprem          -0.14004810 0.30208372 -0.72552215
## hu_is_post1:is_active_training1:UPPSprem  0.71600977 0.34498934  0.04533067
##                                                 Q97.5
## Intercept                                 4.734812279
## hu_Intercept                              1.097726225
## is_post1                                  0.682848055
## is_active_training1                       0.016607802
## UPPSprem                                 -0.009351314
## is_post1:is_active_training1              1.082896950
## is_post1:UPPSprem                         0.179205690
## is_active_training1:UPPSprem              0.525647656
## is_post1:is_active_training1:UPPSprem     0.267392255
## hu_is_post1                               2.713660464
## hu_is_active_training1                    2.277962901
## hu_UPPSprem                               0.531247292
## hu_is_post1:is_active_training1          -0.181574534
## hu_is_post1:UPPSprem                      0.081699604
## hu_is_active_training1:UPPSprem           0.447285413
## hu_is_post1:is_active_training1:UPPSprem  1.404088909
```

```
## Intake - Subscale: UPPS Sensation Seeking
```

```
## Intake ~ is_post * is_active_training * UPPSsen + (is_post | vpNumber) + (is_post | imgNumber) 
## hu ~ is_post * is_active_training * UPPSsen + (is_post | vpNumber) + (is_post | imgNumber)
```

```
##                                            Estimate  Est.Error       Q2.5
## Intercept                                3.54713540 0.18068268  3.1788798
## hu_Intercept                             0.43851145 0.38652004 -0.3281913
## is_post1                                -0.23885916 0.19879319 -0.6324802
## is_active_training1                      0.07975848 0.27113267 -0.4386116
## UPPSsen                                  0.02625426 0.07265893 -0.1133424
## is_post1:is_active_training1             0.20481888 0.30881380 -0.4100112
## is_post1:UPPSsen                         0.04359680 0.08025311 -0.1131703
## is_active_training1:UPPSsen             -0.04442426 0.10729730 -0.2567172
## is_post1:is_active_training1:UPPSsen    -0.10245145 0.12186268 -0.3444637
## hu_is_post1                             -0.16851557 0.41247599 -0.9888239
## hu_is_active_training1                  -0.63820766 0.59694774 -1.8090373
## hu_UPPSsen                              -0.09754914 0.15361115 -0.3931981
## hu_is_post1:is_active_training1          0.72641045 0.66473696 -0.5691787
## hu_is_post1:UPPSsen                      0.12055547 0.16692265 -0.2103056
## hu_is_active_training1:UPPSsen           0.28544230 0.23269373 -0.1710336
## hu_is_post1:is_active_training1:UPPSsen -0.33186042 0.26215799 -0.8720565
##                                             Q97.5
## Intercept                               3.8931283
## hu_Intercept                            1.1909235
## is_post1                                0.1453111
## is_active_training1                     0.6136340
## UPPSsen                                 0.1704941
## is_post1:is_active_training1            0.8108310
## is_post1:UPPSsen                        0.2032846
## is_active_training1:UPPSsen             0.1629853
## is_post1:is_active_training1:UPPSsen    0.1369550
## hu_is_post1                             0.6479201
## hu_is_active_training1                  0.5086953
## hu_UPPSsen                              0.2057367
## hu_is_post1:is_active_training1         2.0487115
## hu_is_post1:UPPSsen                     0.4510862
## hu_is_active_training1:UPPSsen          0.7324700
## hu_is_post1:is_active_training1:UPPSsen 0.1716864
```

```
## Intake - Subscale: UPPS Urgency
```

```
## Intake ~ is_post * is_active_training * UPPSurg + (is_post | vpNumber) + (is_post | imgNumber) 
## hu ~ is_post * is_active_training * UPPSurg + (is_post | vpNumber) + (is_post | imgNumber)
```

```
##                                             Estimate  Est.Error       Q2.5
## Intercept                                3.492093869 0.23194655  3.0461362
## hu_Intercept                             0.897062996 0.50718229 -0.0805198
## is_post1                                -0.102839967 0.24596197 -0.5934314
## is_active_training1                     -0.200147753 0.32492146 -0.8284768
## UPPSurg                                  0.051964026 0.09868846 -0.1352624
## is_post1:is_active_training1             0.245611534 0.34827606 -0.4524576
## is_post1:UPPSurg                        -0.015066459 0.10473352 -0.2212140
## is_active_training1:UPPSurg              0.080871437 0.14105169 -0.2010466
## is_post1:is_active_training1:UPPSurg    -0.133229088 0.15125308 -0.4369910
## hu_is_post1                             -0.007340164 0.55862879 -1.0734362
## hu_is_active_training1                  -0.792121746 0.68084456 -2.1185712
## hu_UPPSurg                              -0.305744014 0.21222208 -0.7271013
## hu_is_post1:is_active_training1          0.856910251 0.76643706 -0.7109932
## hu_is_post1:UPPSurg                      0.056524534 0.24019579 -0.4217585
## hu_is_active_training1:UPPSurg           0.383831792 0.29481502 -0.1964068
## hu_is_post1:is_active_training1:UPPSurg -0.431986777 0.33742691 -1.0751691
##                                              Q97.5
## Intercept                               3.93646430
## hu_Intercept                            1.89042782
## is_post1                                0.37679813
## is_active_training1                     0.44235522
## UPPSurg                                 0.24099606
## is_post1:is_active_training1            0.93615153
## is_post1:UPPSurg                        0.19331707
## is_active_training1:UPPSurg             0.35017625
## is_post1:is_active_training1:UPPSurg    0.17441780
## hu_is_post1                             1.11553087
## hu_is_active_training1                  0.54492783
## hu_UPPSurg                              0.09735915
## hu_is_post1:is_active_training1         2.31342444
## hu_is_post1:UPPSurg                     0.50936526
## hu_is_active_training1:UPPSurg          0.96837643
## hu_is_post1:is_active_training1:UPPSurg 0.25504176
```

**Increase foods:**

```
## Approach Bias - Subscale: UPPS Perseverance
```

```
## bias_stim ~ is_post * is_active_training * UPPSpers + (is_post | vpNumber) + (is_post | imgNumber)
```

```
##                                         Estimate Est.Error       Q2.5     Q97.5
## Intercept                              145.11069  69.82006   12.09362 283.47750
## is_post1                              -129.02601  87.71893 -305.33893  35.91573
## is_active_training1                     79.62260 112.13981 -140.78663 296.84884
## UPPSpers                               -29.92869  23.23702  -76.54863  14.29151
## is_post1:is_active_training1          -109.65569 141.22211 -392.67486 168.10044
## is_post1:UPPSpers                       42.29363  29.04228  -12.88292 100.21185
## is_active_training1:UPPSpers           -21.76410  37.00678  -93.33792  49.94228
## is_post1:is_active_training1:UPPSpers   29.54171  46.14263  -60.25066 120.84546
```

```
## Craving - Subscale: UPPS Perseverance
```

```
## Craving ~ is_post * is_active_training * UPPSpers + (is_post | vpNumber) + (is_post | imgNumber) 
## hu ~ is_post * is_active_training * UPPSpers + (is_post | vpNumber) + (is_post | imgNumber)
```

```
##                                              Estimate  Est.Error        Q2.5
## Intercept                                 3.361588319 0.22880137  2.91004990
## hu_Intercept                             -0.670573510 0.83411256 -2.33715729
## is_post1                                  0.223877127 0.28222830 -0.34187877
## is_active_training1                       0.059909855 0.36012805 -0.62331382
## UPPSpers                                  0.091395811 0.07704718 -0.05732476
## is_post1:is_active_training1             -0.212067250 0.45049332 -1.09656574
## is_post1:UPPSpers                        -0.124496294 0.09479244 -0.31047312
## is_active_training1:UPPSpers             -0.025531913 0.11831426 -0.25437884
## is_post1:is_active_training1:UPPSpers     0.061539183 0.14701984 -0.22192549
## hu_is_post1                               0.099202199 0.78476382 -1.47846040
## hu_is_active_training1                   -0.421070659 1.36348377 -3.12564304
## hu_UPPSpers                              -0.003949239 0.27694054 -0.54928180
## hu_is_post1:is_active_training1           0.966029029 1.22236076 -1.44295033
## hu_is_post1:UPPSpers                      0.020192446 0.26208879 -0.47673665
## hu_is_active_training1:UPPSpers           0.227725158 0.44450418 -0.66325090
## hu_is_post1:is_active_training1:UPPSpers -0.424246333 0.39959738 -1.20235782
##                                               Q97.5
## Intercept                                3.81200816
## hu_Intercept                             0.94497367
## is_post1                                 0.77296116
## is_active_training1                      0.77829870
## UPPSpers                                 0.24109814
## is_post1:is_active_training1             0.67750598
## is_post1:UPPSpers                        0.06316536
## is_active_training1:UPPSpers             0.20049074
## is_post1:is_active_training1:UPPSpers    0.35400766
## hu_is_post1                              1.58245127
## hu_is_active_training1                   2.27692515
## hu_UPPSpers                              0.55219225
## hu_is_post1:is_active_training1          3.32860885
## hu_is_post1:UPPSpers                     0.52898148
## hu_is_active_training1:UPPSpers          1.12876979
## hu_is_post1:is_active_training1:UPPSpers 0.35509029
```

```
## Craving - Subscale: UPPS Premeditation
```

```
## Craving ~ is_post * is_active_training * UPPSprem + (is_post | vpNumber) + (is_post | imgNumber) 
## hu ~ is_post * is_active_training * UPPSprem + (is_post | vpNumber) + (is_post | imgNumber)
```

```
##                                             Estimate  Est.Error       Q2.5
## Intercept                                 3.57798457 0.27361424  3.0443443
## hu_Intercept                              1.01241760 0.96536664 -0.8925634
## is_post1                                  0.19958505 0.35865642 -0.4875445
## is_active_training1                      -0.34860791 0.39592878 -1.1316567
## UPPSprem                                  0.01665856 0.08891001 -0.1575759
## is_post1:is_active_training1             -0.16191939 0.52022356 -1.1769804
## is_post1:UPPSprem                        -0.11130172 0.11550526 -0.3497601
## is_active_training1:UPPSprem              0.11283210 0.12873466 -0.1362034
## is_post1:is_active_training1:UPPSprem     0.03970742 0.16817739 -0.2854468
## hu_is_post1                               1.15312522 0.95332467 -0.7339232
## hu_is_active_training1                   -1.78697045 1.39502488 -4.5642624
## hu_UPPSprem                              -0.56684542 0.31733001 -1.2003177
## hu_is_post1:is_active_training1          -0.55201503 1.35174472 -3.1347295
## hu_is_post1:UPPSprem                     -0.32899176 0.31330136 -0.9518139
## hu_is_active_training1:UPPSprem           0.68687049 0.45422106 -0.2010777
## hu_is_post1:is_active_training1:UPPSprem  0.07692681 0.44115667 -0.8172183
##                                               Q97.5
## Intercept                                4.11126950
## hu_Intercept                             2.96395537
## is_post1                                 0.95577134
## is_active_training1                      0.42445674
## UPPSprem                                 0.19105727
## is_post1:is_active_training1             0.88082986
## is_post1:UPPSprem                        0.10371547
## is_active_training1:UPPSprem             0.36263412
## is_post1:is_active_training1:UPPSprem    0.37026977
## hu_is_post1                              3.07742553
## hu_is_active_training1                   0.93380194
## hu_UPPSprem                              0.06372106
## hu_is_post1:is_active_training1          2.10891811
## hu_is_post1:UPPSprem                     0.28067077
## hu_is_active_training1:UPPSprem          1.59099525
## hu_is_post1:is_active_training1:UPPSprem 0.94192827
```

```
## Craving - Subscale: UPPS Sensation Seeking
```

```
## Craving ~ is_post * is_active_training * UPPSsen + (is_post | vpNumber) + (is_post | imgNumber) 
## hu ~ is_post * is_active_training * UPPSsen + (is_post | vpNumber) + (is_post | imgNumber)
```

```
##                                            Estimate  Est.Error       Q2.5
## Intercept                                3.58517861 0.16605853  3.2591244
## hu_Intercept                            -1.04646717 0.62915504 -2.2916147
## is_post1                                -0.23979602 0.19501242 -0.6235040
## is_active_training1                      0.02038964 0.27384980 -0.5256564
## UPPSsen                                  0.01864092 0.06685458 -0.1128587
## is_post1:is_active_training1             0.02218845 0.33129972 -0.6212247
## is_post1:UPPSsen                         0.04279767 0.07991756 -0.1130750
## is_active_training1:UPPSsen             -0.01059268 0.10624224 -0.2252971
## is_post1:is_active_training1:UPPSsen    -0.02851973 0.12979984 -0.2821117
## hu_is_post1                              0.41049792 0.57639421 -0.7204228
## hu_is_active_training1                   2.24300550 0.95927757  0.3657005
## hu_UPPSsen                               0.15717344 0.25539316 -0.3333853
## hu_is_post1:is_active_training1         -1.64000446 0.89246919 -3.4285217
## hu_is_post1:UPPSsen                     -0.11345302 0.23295688 -0.5799236
## hu_is_active_training1:UPPSsen          -0.78476273 0.38421892 -1.5571359
## hu_is_post1:is_active_training1:UPPSsen  0.53891175 0.35360113 -0.1452775
##                                               Q97.5
## Intercept                                3.90476397
## hu_Intercept                             0.16780586
## is_post1                                 0.14199525
## is_active_training1                      0.56512484
## UPPSsen                                  0.15096848
## is_post1:is_active_training1             0.67067424
## is_post1:UPPSsen                         0.19787517
## is_active_training1:UPPSsen              0.20535041
## is_post1:is_active_training1:UPPSsen     0.22093591
## hu_is_post1                              1.55362536
## hu_is_active_training1                   4.17337100
## hu_UPPSsen                               0.66439268
## hu_is_post1:is_active_training1          0.04880164
## hu_is_post1:UPPSsen                      0.34296541
## hu_is_active_training1:UPPSsen          -0.02473180
## hu_is_post1:is_active_training1:UPPSsen  1.24637842
```

```
## Craving - Subscale: UPPS Urgency
```

```
## Craving ~ is_post * is_active_training * UPPSurg + (is_post | vpNumber) + (is_post | imgNumber) 
## hu ~ is_post * is_active_training * UPPSurg + (is_post | vpNumber) + (is_post | imgNumber)
```

```
##                                             Estimate Est.Error        Q2.5
## Intercept                                3.607960677 0.2221658  3.17818223
## hu_Intercept                            -0.713822601 0.8220411 -2.32258340
## is_post1                                -0.289302645 0.2634156 -0.80622404
## is_active_training1                      0.218463010 0.2993957 -0.36810139
## UPPSurg                                  0.009195378 0.0951042 -0.17589732
## is_post1:is_active_training1            -0.035156081 0.3564698 -0.72909784
## is_post1:UPPSurg                         0.065631763 0.1138491 -0.16442101
## is_active_training1:UPPSurg             -0.100621421 0.1300736 -0.35372124
## is_post1:is_active_training1:UPPSurg    -0.001549163 0.1557203 -0.32017937
## hu_is_post1                              0.917175473 0.7000021 -0.45129789
## hu_is_active_training1                   1.413000722 1.1458409 -0.83199385
## hu_UPPSurg                               0.015436832 0.3488325 -0.67450380
## hu_is_post1:is_active_training1         -2.395365068 1.0024359 -4.44508768
## hu_is_post1:UPPSurg                     -0.339168871 0.3030699 -0.92985009
## hu_is_active_training1:UPPSurg          -0.519572759 0.5000392 -1.52884351
## hu_is_post1:is_active_training1:UPPSurg  0.936398961 0.4404452  0.04695637
##                                              Q97.5
## Intercept                                4.0433112
## hu_Intercept                             0.9054467
## is_post1                                 0.2355201
## is_active_training1                      0.8018335
## UPPSurg                                  0.1915723
## is_post1:is_active_training1             0.6585386
## is_post1:UPPSurg                         0.2889855
## is_active_training1:UPPSurg              0.1545527
## is_post1:is_active_training1:UPPSurg     0.2986886
## hu_is_post1                              2.3121352
## hu_is_active_training1                   3.7377696
## hu_UPPSurg                               0.7072783
## hu_is_post1:is_active_training1         -0.4039238
## hu_is_post1:UPPSurg                      0.2523385
## hu_is_active_training1:UPPSurg           0.4599903
## hu_is_post1:is_active_training1:UPPSurg  1.8179619
```

```
## Intake - Subscale: UPPS Perseverance
```

```
## Intake ~ is_post * is_active_training * UPPSpers + (is_post | vpNumber) + (is_post | imgNumber) 
## hu ~ is_post * is_active_training * UPPSpers + (is_post | vpNumber) + (is_post | imgNumber)
```

```
##                                              Estimate  Est.Error       Q2.5
## Intercept                                 3.547016479 0.25771834  3.0441787
## hu_Intercept                              1.782397229 0.70715658  0.3852479
## is_post1                                 -0.438091374 0.39369370 -1.2318994
## is_active_training1                       0.187389196 0.37140591 -0.5471990
## UPPSpers                                 -0.007898638 0.08482436 -0.1750972
## is_post1:is_active_training1              0.064287644 0.58672059 -1.0977726
## is_post1:UPPSpers                         0.092229837 0.12960933 -0.1609767
## is_active_training1:UPPSpers             -0.023013592 0.12096746 -0.2611492
## is_post1:is_active_training1:UPPSpers    -0.061671098 0.19054357 -0.4431894
## hu_is_post1                              -0.326788654 0.62526159 -1.5659166
## hu_is_active_training1                   -1.731334978 1.08897708 -3.8279651
## hu_UPPSpers                              -0.390497366 0.23551716 -0.8376624
## hu_is_post1:is_active_training1           0.439561740 0.95709989 -1.4800333
## hu_is_post1:UPPSpers                      0.103640650 0.20855043 -0.3048622
## hu_is_active_training1:UPPSpers           0.601758054 0.35828090 -0.1251498
## hu_is_post1:is_active_training1:UPPSpers -0.218182060 0.31093075 -0.8123753
##                                               Q97.5
## Intercept                                4.03627033
## hu_Intercept                             3.13147176
## is_post1                                 0.33487787
## is_active_training1                      0.91276401
## UPPSpers                                 0.16061969
## is_post1:is_active_training1             1.24259104
## is_post1:UPPSpers                        0.35043679
## is_active_training1:UPPSpers             0.21411194
## is_post1:is_active_training1:UPPSpers    0.30951664
## hu_is_post1                              0.89247265
## hu_is_active_training1                   0.45350350
## hu_UPPSpers                              0.07914994
## hu_is_post1:is_active_training1          2.31171104
## hu_is_post1:UPPSpers                     0.51939456
## hu_is_active_training1:UPPSpers          1.29651081
## hu_is_post1:is_active_training1:UPPSpers 0.40173679
```

```
## Intake - Subscale: UPPS Premeditation
```

```
## Intake ~ is_post * is_active_training * UPPSprem + (is_post | vpNumber) + (is_post | imgNumber) 
## hu ~ is_post * is_active_training * UPPSprem + (is_post | vpNumber) + (is_post | imgNumber)
```

```
##                                             Estimate  Est.Error       Q2.5
## Intercept                                 3.40256883 0.30538312  2.8065578
## hu_Intercept                              1.90301021 0.81419080  0.3347950
## is_post1                                  0.33425310 0.44646193 -0.5428666
## is_active_training1                      -0.10089772 0.42354042 -0.9416091
## UPPSprem                                  0.03935039 0.09856009 -0.1524706
## is_post1:is_active_training1             -0.01679745 0.61351011 -1.2016872
## is_post1:UPPSprem                        -0.16376301 0.14389367 -0.4468081
## is_active_training1:UPPSprem              0.07155154 0.13700283 -0.1966234
## is_post1:is_active_training1:UPPSprem    -0.03239237 0.19785276 -0.4198697
## hu_is_post1                              -0.09453563 0.74051566 -1.5430431
## hu_is_active_training1                   -1.64277347 1.19310406 -4.0247748
## hu_UPPSprem                              -0.42271792 0.26498093 -0.9477916
## hu_is_post1:is_active_training1          -0.80861416 1.08157624 -2.9469170
## hu_is_post1:UPPSprem                      0.02416432 0.24052619 -0.4533544
## hu_is_active_training1:UPPSprem           0.56563944 0.38676812 -0.1799563
## hu_is_post1:is_active_training1:UPPSprem  0.19303415 0.34982773 -0.4979749
##                                               Q97.5
## Intercept                                3.99503513
## hu_Intercept                             3.52084981
## is_post1                                 1.21252179
## is_active_training1                      0.72907740
## UPPSprem                                 0.23250263
## is_post1:is_active_training1             1.18336096
## is_post1:UPPSprem                        0.12132307
## is_active_training1:UPPSprem             0.34408593
## is_post1:is_active_training1:UPPSprem    0.35273652
## hu_is_post1                              1.37253474
## hu_is_active_training1                   0.66766788
## hu_UPPSprem                              0.09126228
## hu_is_post1:is_active_training1          1.32954439
## hu_is_post1:UPPSprem                     0.50074781
## hu_is_active_training1:UPPSprem          1.33873170
## hu_is_post1:is_active_training1:UPPSprem 0.88066528
```

```
## Intake - Subscale: UPPS Sensation Seeking
```

```
## Intake ~ is_post * is_active_training * UPPSsen + (is_post | vpNumber) + (is_post | imgNumber) 
## hu ~ is_post * is_active_training * UPPSsen + (is_post | vpNumber) + (is_post | imgNumber)
```

```
##                                            Estimate  Est.Error        Q2.5
## Intercept                                3.59436087 0.16325508  3.27831865
## hu_Intercept                            -0.32614599 0.50745954 -1.31394311
## is_post1                                -0.52209424 0.23186663 -0.97755641
## is_active_training1                      0.15605035 0.27456042 -0.38567024
## UPPSsen                                 -0.03231257 0.06685787 -0.16328956
## is_post1:is_active_training1             0.24172359 0.39199321 -0.54603967
## is_post1:UPPSsen                         0.15425624 0.09531712 -0.02866853
## is_active_training1:UPPSsen             -0.01085588 0.10775361 -0.21958636
## is_post1:is_active_training1:UPPSsen    -0.15396165 0.15459760 -0.45990024
## hu_is_post1                              0.96151507 0.41834363  0.11993610
## hu_is_active_training1                   2.00881488 0.80341948  0.40497868
## hu_UPPSsen                               0.41366576 0.20152336  0.01439665
## hu_is_post1:is_active_training1         -1.67709292 0.69714936 -3.00962180
## hu_is_post1:UPPSsen                     -0.41949107 0.16906257 -0.74737503
## hu_is_active_training1:UPPSsen          -0.80167886 0.31528611 -1.42107702
## hu_is_post1:is_active_training1:UPPSsen  0.60857207 0.27292655  0.07631670
##                                               Q97.5
## Intercept                                3.91422692
## hu_Intercept                             0.67287211
## is_post1                                -0.06483778
## is_active_training1                      0.68963823
## UPPSsen                                  0.09892893
## is_post1:is_active_training1             1.01765949
## is_post1:UPPSsen                         0.34372669
## is_active_training1:UPPSsen              0.20097872
## is_post1:is_active_training1:UPPSsen     0.15286355
## hu_is_post1                              1.76596713
## hu_is_active_training1                   3.61591706
## hu_UPPSsen                               0.82440607
## hu_is_post1:is_active_training1         -0.30320190
## hu_is_post1:UPPSsen                     -0.08240467
## hu_is_active_training1:UPPSsen          -0.17468693
## hu_is_post1:is_active_training1:UPPSsen  1.13804160
```

```
## Intake - Subscale: UPPS Urgency
```

```
## Intake ~ is_post * is_active_training * UPPSurg + (is_post | vpNumber) + (is_post | imgNumber) 
## hu ~ is_post * is_active_training * UPPSurg + (is_post | vpNumber) + (is_post | imgNumber)
```

```
##                                             Estimate  Est.Error       Q2.5
## Intercept                                3.507084254 0.22527384  3.0632175
## hu_Intercept                             0.441238270 0.65940148 -0.8511975
## is_post1                                 0.017918625 0.32668785 -0.6485873
## is_active_training1                      0.100709334 0.30581087 -0.4900830
## UPPSurg                                  0.008573257 0.09763586 -0.1866782
## is_post1:is_active_training1            -0.130929059 0.43713553 -0.9926482
## is_post1:UPPSurg                        -0.082272092 0.14086416 -0.3549860
## is_active_training1:UPPSurg              0.006378887 0.13255009 -0.2539451
## is_post1:is_active_training1:UPPSurg     0.005753890 0.18890384 -0.3859609
## hu_is_post1                              0.408424919 0.56458236 -0.7007218
## hu_is_active_training1                   0.476913825 0.90008120 -1.2841462
## hu_UPPSurg                               0.091355145 0.28098023 -0.4673254
## hu_is_post1:is_active_training1         -0.923069601 0.78977255 -2.4474802
## hu_is_post1:UPPSurg                     -0.190568563 0.24377930 -0.6700530
## hu_is_active_training1:UPPSurg          -0.193295757 0.39363432 -0.9838192
## hu_is_post1:is_active_training1:UPPSurg  0.317797469 0.34428393 -0.3541008
##                                             Q97.5
## Intercept                               3.9492015
## hu_Intercept                            1.7635630
## is_post1                                0.6479845
## is_active_training1                     0.7174266
## UPPSurg                                 0.2007714
## is_post1:is_active_training1            0.7679250
## is_post1:UPPSurg                        0.2036238
## is_active_training1:UPPSurg             0.2643224
## is_post1:is_active_training1:UPPSurg    0.3764433
## hu_is_post1                             1.5436435
## hu_is_active_training1                  2.2754693
## hu_UPPSurg                              0.6442679
## hu_is_post1:is_active_training1         0.6220165
## hu_is_post1:UPPSurg                     0.2974827
## hu_is_active_training1:UPPSurg          0.5729209
## hu_is_post1:is_active_training1:UPPSurg 0.9809681
```

## Participant-specific moderators related to the training

Contingency awareness, Expectancy, and Intentions were modeled as
potential moderators that were assessed on a participant-level but
relate to the training.

### Methods

As an assessment of contingency awareness, participants were asked
after the Follow-up AAA session for each food image, how often they
thought they had pushed it away from themselves throughout the whole
trial period on a scale from 0 to 100 %. These values were averaged
separately for “increase” and “decrease” foods and then subtracted from
one another, such that the resulting value indicates a belief to have
pushed away decrease foods more often than increase foods.

Expectancy was assessed with the question “How much do you expect the
task to help you reach your dietary goals on a scale from 1-5 where 1
was labeled “not at all” and 5 was labeled “very much”. The two values
(at the start and the end of the intervention period) were averaged.

For the assessment of intentions, we asked participants during the
ecological momentary assessment at midday and in the evening with the
question “How much do you want to stick to your dietary goal for the
rest of the day/for the next day?” using a 0-100 slider. For each
participant, we averaged all intention values reported to arrive at a
between-person variable.

### Contingency awareness

**Decrease foods:**

```
## Approach Bias
```

```
## bias_stim ~ is_post * is_active_training * contingency_de + (is_post | vpNumber) + (is_post | imgNumber)
```

```
##                                               Estimate Est.Error        Q2.5
## Intercept                                   58.8247957 56.542276  -51.544051
## is_post1                                    -7.4740779 64.609499 -135.454594
## is_active_training1                         21.6587561 69.605739 -111.423964
## contingency_de                              -0.1744325  1.064827   -2.178047
## is_post1:is_active_training1                -6.0663270 79.952110 -160.810266
## is_post1:contingency_de                      0.2432397  1.233398   -2.172070
## is_active_training1:contingency_de          -0.1897471  1.332717   -2.881399
## is_post1:is_active_training1:contingency_de -0.8978692  1.547899   -3.897327
##                                                  Q97.5
## Intercept                                   165.940875
## is_post1                                    119.224885
## is_active_training1                         158.188123
## contingency_de                                1.935305
## is_post1:is_active_training1                146.098318
## is_post1:contingency_de                       2.712284
## is_active_training1:contingency_de            2.321292
## is_post1:is_active_training1:contingency_de   2.239062
```

```
## Craving
```

```
## Craving ~ is_post * is_active_training * contingency_de + (is_post | vpNumber) + (is_post | imgNumber) 
## hu ~ is_post * is_active_training * contingency_de + (is_post | vpNumber) + (is_post | imgNumber)
```

```
##                                                    Estimate   Est.Error
## Intercept                                       3.053953686 1.213402854
## hu_Intercept                                    2.434612384 5.644198544
## is_post1                                       -0.326174435 0.261965202
## is_active_training1                             0.618383272 0.728901526
## contingency_de                                  0.005620588 0.009087798
## is_post1:is_active_training1                    0.105835371 0.740241151
## is_post1:contingency_de                        -0.003202252 0.011538698
## is_active_training1:contingency_de             -0.011933595 0.013020491
## is_post1:is_active_training1:contingency_de    -0.005212272 0.013401857
## hu_is_post1                                     1.139432953 0.634611013
## hu_is_active_training1                         -0.323337429 0.796433688
## hu_contingency_de                              -0.030501834 0.036877619
## hu_is_post1:is_active_training1                -1.018265720 0.680822731
## hu_is_post1:contingency_de                     -0.254874958 0.426973390
## hu_is_active_training1:contingency_de           0.357622609 0.602997021
## hu_is_post1:is_active_training1:contingency_de -0.323435879 0.583734834
##                                                        Q2.5        Q97.5
## Intercept                                       0.348624388  4.060842911
## hu_Intercept                                   -2.077255972 12.152080671
## is_post1                                       -0.673977684  0.156129621
## is_active_training1                            -0.192336812  1.833607682
## contingency_de                                 -0.005294038  0.024946043
## is_post1:is_active_training1                   -0.754554994  1.336364296
## is_post1:contingency_de                        -0.032763108  0.008657581
## is_active_training1:contingency_de             -0.034793910  0.002983489
## is_post1:is_active_training1:contingency_de    -0.031768527  0.010314178
## hu_is_post1                                    -0.187225780  2.182734442
## hu_is_active_training1                         -1.921462687  1.396927227
## hu_contingency_de                              -0.091200412  0.012896405
## hu_is_post1:is_active_training1                -2.523973980  0.364099466
## hu_is_post1:contingency_de                     -0.994157487  0.013051177
## hu_is_active_training1:contingency_de          -0.020916110  1.401671599
## hu_is_post1:is_active_training1:contingency_de -1.334152806  0.042065536
```

```
## Intake
```

```
## Intake ~ is_post * is_active_training * contingency_de + (is_post | vpNumber) + (is_post | imgNumber) 
## hu ~ is_post * is_active_training * contingency_de + (is_post | vpNumber) + (is_post | imgNumber)
```

```
##                                                    Estimate   Est.Error
## Intercept                                       2.831578876 1.089750045
## hu_Intercept                                    3.532401095 4.994189956
## is_post1                                       -0.189259348 0.355091797
## is_active_training1                             0.603693906 0.749926438
## contingency_de                                  0.009021286 0.010731642
## is_post1:is_active_training1                    0.282291242 0.664009925
## is_post1:contingency_de                        -0.008958397 0.012624403
## is_active_training1:contingency_de             -0.013706273 0.016140785
## is_post1:is_active_training1:contingency_de    -0.004374466 0.009566432
## hu_is_post1                                     0.653269245 0.733979501
## hu_is_active_training1                         -0.481443032 0.499861206
## hu_contingency_de                              -0.029953973 0.036155694
## hu_is_post1:is_active_training1                -0.470179475 0.624396238
## hu_is_post1:contingency_de                     -0.251618866 0.428829388
## hu_is_active_training1:contingency_de           0.358644120 0.602288525
## hu_is_post1:is_active_training1:contingency_de -0.330459254 0.579637412
##                                                        Q2.5        Q97.5
## Intercept                                       0.465503908  3.847749625
## hu_Intercept                                   -0.201843675 12.152192438
## is_post1                                       -0.674061001  0.433859123
## is_active_training1                            -0.308715837  1.833247046
## contingency_de                                 -0.004209113  0.030067658
## is_post1:is_active_training1                   -0.625919866  1.336208660
## is_post1:contingency_de                        -0.038058462  0.006091895
## is_active_training1:contingency_de             -0.040986468  0.004467050
## is_post1:is_active_training1:contingency_de    -0.019935858  0.010714270
## hu_is_post1                                    -0.638978078  1.646837463
## hu_is_active_training1                         -1.536907961  0.564397292
## hu_contingency_de                              -0.091213366  0.005846046
## hu_is_post1:is_active_training1                -1.504042949  0.842641140
## hu_is_post1:contingency_de                     -0.994154721  0.013033523
## hu_is_active_training1:contingency_de          -0.007857495  1.401591446
## hu_is_post1:is_active_training1:contingency_de -1.334145373  0.026454205
```

**Increase foods:**

```
## Approach Bias
```

```
## bias_stim ~ is_post * is_active_training * contingency_in + (is_post | vpNumber) + (is_post | imgNumber)
```

```
##                                                 Estimate  Est.Error        Q2.5
## Intercept                                    79.47325965 37.4086346    5.645122
## is_post1                                      0.45701821 48.7730478  -97.046933
## is_active_training1                          15.17877589 48.4205917  -76.008008
## contingency_in                               -0.64058747  0.8471743   -2.304109
## is_post1:is_active_training1                -36.77389892 60.6877763 -152.533721
## is_post1:contingency_in                       0.09038489  1.0877972   -2.003851
## is_active_training1:contingency_in           -0.27997105  1.1769148   -2.678309
## is_post1:is_active_training1:contingency_in   0.67692165  1.4936219   -2.227345
##                                                  Q97.5
## Intercept                                   153.359502
## is_post1                                     90.528926
## is_active_training1                         112.514499
## contingency_in                                1.083899
## is_post1:is_active_training1                 82.462611
## is_post1:contingency_in                       2.203062
## is_active_training1:contingency_in            1.888881
## is_post1:is_active_training1:contingency_in   3.688389
```

```
## Craving
```

```
## Craving ~ is_post * is_active_training * contingency_in + (is_post | vpNumber) + (is_post | imgNumber) 
## hu ~ is_post * is_active_training * contingency_in + (is_post | vpNumber) + (is_post | imgNumber)
```

```
##                                                    Estimate   Est.Error
## Intercept                                       3.672369405 0.126965160
## hu_Intercept                                   -0.402442941 0.506934607
## is_post1                                       -0.034580257 0.162282817
## is_active_training1                            -0.164351364 0.163670271
## contingency_in                                 -0.001714722 0.002787812
## is_post1:is_active_training1                   -0.123320891 0.210161064
## is_post1:contingency_in                        -0.002488122 0.003566164
## is_active_training1:contingency_in              0.005448201 0.003994746
## is_post1:is_active_training1:contingency_in     0.001169645 0.005098178
## hu_is_post1                                     0.583489337 0.455679097
## hu_is_active_training1                         -0.272465724 0.633748413
## hu_contingency_in                              -0.009209957 0.011411672
## hu_is_post1:is_active_training1                -0.610414717 0.571533441
## hu_is_post1:contingency_in                     -0.010184479 0.010375515
## hu_is_active_training1:contingency_in           0.015242904 0.015526111
## hu_is_post1:is_active_training1:contingency_in  0.005202653 0.014032626
##                                                        Q2.5       Q97.5
## Intercept                                       3.425721492 3.922297575
## hu_Intercept                                   -1.392329043 0.555413175
## is_post1                                       -0.359755594 0.287396706
## is_active_training1                            -0.493942265 0.145501552
## contingency_in                                 -0.007350535 0.003892806
## is_post1:is_active_training1                   -0.535218978 0.281924290
## is_post1:contingency_in                        -0.009392745 0.004802626
## is_active_training1:contingency_in             -0.002158893 0.013409170
## is_post1:is_active_training1:contingency_in    -0.008729447 0.011192014
## hu_is_post1                                    -0.341518388 1.473266218
## hu_is_active_training1                         -1.514764664 0.963276340
## hu_contingency_in                              -0.031678192 0.013131816
## hu_is_post1:is_active_training1                -1.729163074 0.523244860
## hu_is_post1:contingency_in                     -0.030946895 0.010512108
## hu_is_active_training1:contingency_in          -0.015514997 0.044939439
## hu_is_post1:is_active_training1:contingency_in -0.022074156 0.032581220
```

```
## Intake
```

```
## Intake ~ is_post * is_active_training * contingency_in + (is_post | vpNumber) + (is_post | imgNumber) 
## hu ~ is_post * is_active_training * contingency_in + (is_post | vpNumber) + (is_post | imgNumber)
```

```
##                                                     Estimate   Est.Error
## Intercept                                       3.7037226552 0.133824827
## hu_Intercept                                    1.4466154835 0.427210435
## is_post1                                        0.0555290429 0.199300060
## is_active_training1                            -0.2576463683 0.163767197
## contingency_in                                 -0.0042442988 0.002819918
## is_post1:is_active_training1                   -0.4098169607 0.246073844
## is_post1:contingency_in                        -0.0059389841 0.004215048
## is_active_training1:contingency_in              0.0103220384 0.003895790
## is_post1:is_active_training1:contingency_in     0.0072017125 0.005889253
## hu_is_post1                                    -0.0821528881 0.352235351
## hu_is_active_training1                         -0.8443904490 0.525180477
## hu_contingency_in                              -0.0194062763 0.009133681
## hu_is_post1:is_active_training1                 0.0318866224 0.440180008
## hu_is_post1:contingency_in                      0.0006421083 0.007847426
## hu_is_active_training1:contingency_in           0.0239396200 0.012594890
## hu_is_post1:is_active_training1:contingency_in -0.0076262842 0.010641785
##                                                         Q2.5        Q97.5
## Intercept                                       3.4357402899  3.961133096
## hu_Intercept                                    0.6095267898  2.270046280
## is_post1                                       -0.3453815140  0.453480983
## is_active_training1                            -0.5813436663  0.069539483
## contingency_in                                 -0.0098598369  0.001355609
## is_post1:is_active_training1                   -0.8968042527  0.080798575
## is_post1:contingency_in                        -0.0142922966  0.002509552
## is_active_training1:contingency_in              0.0026204023  0.017947958
## is_post1:is_active_training1:contingency_in    -0.0041103482  0.018814781
## hu_is_post1                                    -0.7793797718  0.606757070
## hu_is_active_training1                         -1.8579737569  0.210713951
## hu_contingency_in                              -0.0378795649 -0.001545551
## hu_is_post1:is_active_training1                -0.8216307613  0.885695688
## hu_is_post1:contingency_in                     -0.0147040073  0.016176591
## hu_is_active_training1:contingency_in          -0.0006600855  0.048743809
## hu_is_post1:is_active_training1:contingency_in -0.0285146365  0.013395338
```

### Expectancy

**Decrease foods:**

```
## Approach Bias
```

```
## bias_stim ~ is_post * is_active_training * expectancy + (is_post | vpNumber) + (is_post | imgNumber)
```

```
##                                           Estimate Est.Error       Q2.5
## Intercept                                42.901970  38.42833  -31.64869
## is_post1                                 52.898924  42.89430  -30.81153
## is_active_training1                      45.551672  56.92172  -66.53004
## expectancy                                3.406105  15.26304  -26.08224
## is_post1:is_active_training1            -79.678505  61.41722 -198.69006
## is_post1:expectancy                     -21.501313  16.80159  -54.84927
## is_active_training1:expectancy          -15.329825  21.71677  -56.13426
## is_post1:is_active_training1:expectancy  20.906030  23.68028  -24.91413
##                                             Q97.5
## Intercept                               118.65393
## is_post1                                138.75140
## is_active_training1                     160.04909
## expectancy                               32.07782
## is_post1:is_active_training1             37.99096
## is_post1:expectancy                      11.74886
## is_active_training1:expectancy           28.25580
## is_post1:is_active_training1:expectancy  66.58675
```

```
## Craving
```

```
## Craving ~ is_post * is_active_training * expectancy + (is_post | vpNumber) + (is_post | imgNumber) 
## hu ~ is_post * is_active_training * expectancy + (is_post | vpNumber) + (is_post | imgNumber)
```

```
##                                               Estimate  Est.Error        Q2.5
## Intercept                                   3.74052060 0.11520981  3.51498214
## hu_Intercept                               -0.74036792 0.46735540 -1.66689028
## is_post1                                   -0.03438614 0.12413834 -0.26636383
## is_active_training1                         0.03403456 0.17209728 -0.30602389
## expectancy                                  0.02458819 0.04237348 -0.05768897
## is_post1:is_active_training1               -0.31839483 0.19044696 -0.69750047
## is_post1:expectancy                        -0.03193675 0.04664662 -0.12615613
## is_active_training1:expectancy             -0.03116247 0.06438107 -0.15520793
## is_post1:is_active_training1:expectancy     0.04878051 0.06986585 -0.08697332
## hu_is_post1                                 1.24178008 0.42298154  0.41165744
## hu_is_active_training1                      0.70799824 0.67444985 -0.56144414
## hu_expectancy                              -0.20235772 0.17775856 -0.56426706
## hu_is_post1:is_active_training1            -1.15928382 0.63383275 -2.39566785
## hu_is_post1:expectancy                     -0.37902842 0.16786281 -0.71363102
## hu_is_active_training1:expectancy          -0.14402741 0.25806588 -0.66223167
## hu_is_post1:is_active_training1:expectancy  0.43044017 0.24652111 -0.03589295
##                                                  Q97.5
## Intercept                                   3.97101012
## hu_Intercept                                0.17632387
## is_post1                                    0.21524445
## is_active_training1                         0.36107300
## expectancy                                  0.10828342
## is_post1:is_active_training1                0.05176793
## is_post1:expectancy                         0.05745663
## is_active_training1:expectancy              0.09329667
## is_post1:is_active_training1:expectancy     0.18767644
## hu_is_post1                                 2.09940185
## hu_is_active_training1                      2.08411397
## hu_expectancy                               0.14667428
## hu_is_post1:is_active_training1             0.07354637
## hu_is_post1:expectancy                     -0.05555106
## hu_is_active_training1:expectancy           0.34916822
## hu_is_post1:is_active_training1:expectancy  0.91188328
```

```
## Intake
```

```
## Intake ~ is_post * is_active_training * expectancy + (is_post | vpNumber) + (is_post | imgNumber) 
## hu ~ is_post * is_active_training * expectancy + (is_post | vpNumber) + (is_post | imgNumber)
```

```
##                                               Estimate  Est.Error         Q2.5
## Intercept                                   3.68165499 0.13967706  3.408524074
## hu_Intercept                                0.23765956 0.32537139 -0.410749011
## is_post1                                   -0.13486145 0.15895884 -0.438854596
## is_active_training1                         0.04293254 0.21094357 -0.367264369
## expectancy                                 -0.02788244 0.05318734 -0.129655318
## is_post1:is_active_training1               -0.10707284 0.24309054 -0.580364162
## is_post1:expectancy                        -0.00441948 0.05945072 -0.122585248
## is_active_training1:expectancy             -0.02446531 0.07907386 -0.183386997
## is_post1:is_active_training1:expectancy     0.02684520 0.09028408 -0.152308591
## hu_is_post1                                 0.60193862 0.32199764 -0.005454216
## hu_is_active_training1                      0.38903798 0.46202584 -0.505513122
## hu_expectancy                              -0.02242331 0.11938991 -0.249108488
## hu_is_post1:is_active_training1            -0.69090582 0.47224953 -1.632766037
## hu_is_post1:expectancy                     -0.22506121 0.12394639 -0.469056687
## hu_is_active_training1:expectancy          -0.13177733 0.17124965 -0.478646720
## hu_is_post1:is_active_training1:expectancy  0.30333827 0.17892433 -0.040147781
##                                                 Q97.5
## Intercept                                  3.95346490
## hu_Intercept                               0.85808180
## is_post1                                   0.17991512
## is_active_training1                        0.45804701
## expectancy                                 0.07631779
## is_post1:is_active_training1               0.36774590
## is_post1:expectancy                        0.11391668
## is_active_training1:expectancy             0.12652705
## is_post1:is_active_training1:expectancy    0.20395377
## hu_is_post1                                1.23939504
## hu_is_active_training1                     1.31807984
## hu_expectancy                              0.21465064
## hu_is_post1:is_active_training1            0.22172930
## hu_is_post1:expectancy                     0.00713763
## hu_is_active_training1:expectancy          0.19332612
## hu_is_post1:is_active_training1:expectancy 0.65084577
```

**Increase foods:**

```
## Approach Bias
```

```
## bias_stim ~ is_post * is_active_training * expectancy + (is_post | vpNumber) + (is_post | imgNumber)
```

```
##                                            Estimate Est.Error        Q2.5
## Intercept                                  7.048858  41.72560  -77.507389
## is_post1                                  61.109852  52.85484  -42.987214
## is_active_training1                       95.698508  63.05002  -26.093716
## expectancy                                21.499159  15.76793   -9.347235
## is_post1:is_active_training1            -163.099982  79.00531 -322.556386
## is_post1:expectancy                      -27.963274  20.00796  -67.320033
## is_active_training1:expectancy           -37.278731  24.02758  -86.095761
## is_post1:is_active_training1:expectancy   63.162035  30.05507    7.438932
##                                              Q97.5
## Intercept                                89.466862
## is_post1                                163.526331
## is_active_training1                     221.002727
## expectancy                               52.951495
## is_post1:is_active_training1            -14.254162
## is_post1:expectancy                      10.909024
## is_active_training1:expectancy            8.473973
## is_post1:is_active_training1:expectancy 124.321750
```

```
## Craving
```

```
## Craving ~ is_post * is_active_training * expectancy + (is_post | vpNumber) + (is_post | imgNumber) 
## hu ~ is_post * is_active_training * expectancy + (is_post | vpNumber) + (is_post | imgNumber)
```

```
##                                                Estimate  Est.Error        Q2.5
## Intercept                                   3.508863419 0.13540463  3.23975275
## hu_Intercept                               -0.091974213 0.49006049 -1.06563867
## is_post1                                   -0.133746998 0.15873241 -0.44329467
## is_active_training1                         0.163235021 0.20475965 -0.24283951
## expectancy                                  0.050170790 0.05118422 -0.05110580
## is_post1:is_active_training1               -0.452885728 0.24227758 -0.94081914
## is_post1:expectancy                        -0.001520963 0.05873711 -0.10886506
## is_active_training1:expectancy             -0.076434197 0.07703123 -0.22312805
## is_post1:is_active_training1:expectancy     0.159254833 0.08867348 -0.01547469
## hu_is_post1                                 0.964653808 0.43981259  0.06090065
## hu_is_active_training1                      0.296100308 0.72548823 -1.11090903
## hu_expectancy                              -0.276018173 0.19094974 -0.65633960
## hu_is_post1:is_active_training1             0.281239861 0.63124822 -0.92409660
## hu_is_post1:expectancy                     -0.342524432 0.16987491 -0.66692818
## hu_is_active_training1:expectancy           0.043520648 0.27850569 -0.48331193
## hu_is_post1:is_active_training1:expectancy -0.220191968 0.24516883 -0.70257172
##                                                    Q97.5
## Intercept                                   3.777943e+00
## hu_Intercept                                8.785505e-01
## is_post1                                    1.625335e-01
## is_active_training1                         5.556899e-01
## expectancy                                  1.525351e-01
## is_post1:is_active_training1                3.289284e-02
## is_post1:expectancy                         1.159015e-01
## is_active_training1:expectancy              7.409493e-02
## is_post1:is_active_training1:expectancy     3.380126e-01
## hu_is_post1                                 1.812900e+00
## hu_is_active_training1                      1.695473e+00
## hu_expectancy                               9.643055e-02
## hu_is_post1:is_active_training1             1.514869e+00
## hu_is_post1:expectancy                     -8.727175e-05
## hu_is_active_training1:expectancy           5.769326e-01
## hu_is_post1:is_active_training1:expectancy  2.550764e-01
```

```
## Intake
```

```
## Intake ~ is_post * is_active_training * expectancy + (is_post | vpNumber) + (is_post | imgNumber) 
## hu ~ is_post * is_active_training * expectancy + (is_post | vpNumber) + (is_post | imgNumber)
```

```
##                                                Estimate  Est.Error        Q2.5
## Intercept                                   3.662541424 0.13112224  3.40556316
## hu_Intercept                                0.768927111 0.40101979 -0.03558961
## is_post1                                   -0.297439112 0.19796115 -0.70350260
## is_active_training1                        -0.105469071 0.19664384 -0.48390355
## expectancy                                 -0.046177663 0.04747802 -0.13998872
## is_post1:is_active_training1               -0.086714322 0.30274149 -0.66640769
## is_post1:expectancy                         0.041434659 0.07293204 -0.10045694
## is_active_training1:expectancy              0.076419263 0.07212721 -0.06830864
## is_post1:is_active_training1:expectancy    -0.006089527 0.11101060 -0.22602628
## hu_is_post1                                 0.535994816 0.32559391 -0.09775216
## hu_is_active_training1                      0.161682211 0.58205098 -0.95472370
## hu_expectancy                              -0.062643021 0.15331942 -0.36106104
## hu_is_post1:is_active_training1            -0.099604996 0.49775942 -1.06084658
## hu_is_post1:expectancy                     -0.253670624 0.12518382 -0.49842288
## hu_is_active_training1:expectancy          -0.020276777 0.22018124 -0.46370099
## hu_is_post1:is_active_training1:expectancy -0.001129036 0.18776262 -0.37514613
##                                                   Q97.5
## Intercept                                   3.914725035
## hu_Intercept                                1.561064785
## is_post1                                    0.072565877
## is_active_training1                         0.294736165
## expectancy                                  0.045418066
## is_post1:is_active_training1                0.515925720
## is_post1:expectancy                         0.185353643
## is_active_training1:expectancy              0.216840807
## is_post1:is_active_training1:expectancy     0.209097902
## hu_is_post1                                 1.172968592
## hu_is_active_training1                      1.346784522
## hu_expectancy                               0.251096240
## hu_is_post1:is_active_training1             0.880636675
## hu_is_post1:expectancy                     -0.003886409
## hu_is_active_training1:expectancy           0.405163303
## hu_is_post1:is_active_training1:expectancy  0.363285913
```

### Intentions

**Decrease foods:**

```
## Approach Bias
```

```
## bias_stim ~ is_post * is_active_training * intentionBS + (is_post | vpNumber) + (is_post | imgNumber)
```

```
##                                              Estimate  Est.Error        Q2.5
## Intercept                                 49.47868523 58.4146769  -64.578415
## is_post1                                   0.55289217 65.8760541 -126.434441
## is_active_training1                       63.37304713 85.7625281 -109.650986
## intentionBS                                0.04871031  0.7998388   -1.502511
## is_post1:is_active_training1             -60.56814458 95.7509389 -240.453627
## is_post1:intentionBS                      -0.01158485  0.8977162   -1.669276
## is_active_training1:intentionBS           -0.78833696  1.2210153   -3.173934
## is_post1:is_active_training1:intentionBS   0.34030392  1.3655303   -2.409602
##                                               Q97.5
## Intercept                                159.868350
## is_post1                                 127.698336
## is_active_training1                      234.959982
## intentionBS                                1.656227
## is_post1:is_active_training1             135.769911
## is_post1:intentionBS                       1.758258
## is_active_training1:intentionBS            1.681828
## is_post1:is_active_training1:intentionBS   2.875580
```

**Increase foods:**

```
## Approach Bias
```

```
## bias_stim ~ is_post * is_active_training * intentionBS + (is_post | vpNumber) + (is_post | imgNumber)
```

```
##                                              Estimate   Est.Error        Q2.5
## Intercept                                  37.9270767  63.3926994  -84.240471
## is_post1                                   15.0708708  80.6169995 -142.696483
## is_active_training1                        89.2263612  89.3021458  -82.748954
## intentionBS                                 0.2802363   0.8692647   -1.392019
## is_post1:is_active_training1             -119.9175110 114.0480173 -339.499684
## is_post1:intentionBS                       -0.2926646   1.1074424   -2.477599
## is_active_training1:intentionBS            -1.1466324   1.2403214   -3.624954
## is_post1:is_active_training1:intentionBS    1.5339355   1.5915845   -1.716884
##                                               Q97.5
## Intercept                                160.942700
## is_post1                                 169.081045
## is_active_training1                      273.564754
## intentionBS                                1.933581
## is_post1:is_active_training1             112.471689
## is_post1:intentionBS                       1.903564
## is_active_training1:intentionBS            1.270037
## is_post1:is_active_training1:intentionBS   4.535353
```

## Stimulus-specific moderators

We also examined stimulus-specific moderators, namely training status
(i.e., whether the stimulus was included in the AAT or AAI during the
intervention period) and baseline stimulus-specific craving.

### Training status

**Decrease foods:**

```
## Approach Bias
```

```
## bias_stim ~ is_post * is_active_training * is_untrained + (is_post | vpNumber) + (is_post | imgNumber)
```

```
##                                              Estimate Est.Error       Q2.5
## Intercept                                   49.796320 15.895949  20.473241
## is_post1                                     9.467923 18.582636 -26.765363
## is_active_training1                          8.504671 22.031940 -34.554721
## is_untrained1                                4.483461  7.328294  -9.906455
## is_post1:is_active_training1               -51.054867 24.822886 -98.635888
## is_post1:is_untrained1                     -23.291499 10.752485 -43.985615
## is_active_training1:is_untrained1            4.216877 10.571715 -16.458214
## is_post1:is_active_training1:is_untrained1  42.241467 15.310151  12.433181
##                                                Q97.5
## Intercept                                  81.823796
## is_post1                                   45.937958
## is_active_training1                        49.885331
## is_untrained1                              19.298037
## is_post1:is_active_training1               -2.311962
## is_post1:is_untrained1                     -1.945567
## is_active_training1:is_untrained1          25.287730
## is_post1:is_active_training1:is_untrained1 71.415548
```

```
## Craving
```

```
## Craving ~ is_post * is_active_training * is_untrained + (is_post | vpNumber) + (is_post | imgNumber) 
## hu ~ is_post * is_active_training * is_untrained + (is_post | vpNumber) + (is_post | imgNumber)
```

```
##                                                   Estimate  Est.Error
## Intercept                                      3.786065834 0.04779722
## hu_Intercept                                  -1.226496418 0.19257539
## is_post1                                      -0.099972480 0.05136813
## is_active_training1                           -0.075978368 0.06535680
## is_untrained1                                 -0.019324660 0.04286589
## is_post1:is_active_training1                  -0.168470936 0.07384994
## is_post1:is_untrained1                        -0.005606273 0.06477186
## is_active_training1:is_untrained1              0.098240790 0.06136279
## is_post1:is_active_training1:is_untrained1    -0.061239703 0.09218639
## hu_is_post1                                    0.346989053 0.17980583
## hu_is_active_training1                         0.323533499 0.24248092
## hu_is_untrained1                               0.036446480 0.13589858
## hu_is_post1:is_active_training1               -0.239192188 0.25370983
## hu_is_post1:is_untrained1                      0.308126348 0.18733319
## hu_is_active_training1:is_untrained1          -0.266411625 0.19454061
## hu_is_post1:is_active_training1:is_untrained1 -0.054958378 0.27193142
##                                                      Q2.5        Q97.5
## Intercept                                      3.69176545  3.880635852
## hu_Intercept                                  -1.60577861 -0.859289550
## is_post1                                      -0.20033315  0.002025355
## is_active_training1                           -0.20173619  0.051756943
## is_untrained1                                 -0.10278086  0.064439861
## is_post1:is_active_training1                  -0.31731211 -0.027191766
## is_post1:is_untrained1                        -0.12929727  0.127343917
## is_active_training1:is_untrained1             -0.02576389  0.221956447
## is_post1:is_active_training1:is_untrained1    -0.24277778  0.116932073
## hu_is_post1                                   -0.01181642  0.701335212
## hu_is_active_training1                        -0.14183343  0.797448325
## hu_is_untrained1                              -0.22881469  0.299511383
## hu_is_post1:is_active_training1               -0.73927984  0.246442880
## hu_is_post1:is_untrained1                     -0.06818637  0.679971480
## hu_is_active_training1:is_untrained1          -0.63945579  0.113691999
## hu_is_post1:is_active_training1:is_untrained1 -0.57107850  0.493579783
```

```
## Intake
```

```
## Intake ~ is_post * is_active_training * is_untrained + (is_post | vpNumber) + (is_post | imgNumber) 
## hu ~ is_post * is_active_training * is_untrained + (is_post | vpNumber) + (is_post | imgNumber)
```

```
##                                                   Estimate  Est.Error
## Intercept                                      3.624718990 0.05991365
## hu_Intercept                                   0.191538009 0.15020185
## is_post1                                      -0.138287113 0.06890315
## is_active_training1                           -0.076759318 0.08103354
## is_untrained1                                 -0.042512754 0.06588951
## is_post1:is_active_training1                  -0.005860359 0.09636530
## is_post1:is_untrained1                        -0.010458672 0.09741333
## is_active_training1:is_untrained1              0.138672451 0.09418582
## is_post1:is_active_training1:is_untrained1    -0.093782743 0.13918260
## hu_is_post1                                   -0.074850600 0.13781913
## hu_is_active_training1                         0.081462669 0.16855742
## hu_is_untrained1                              -0.015657513 0.11375980
## hu_is_post1:is_active_training1                0.063862021 0.19125971
## hu_is_post1:is_untrained1                      0.612014971 0.16340197
## hu_is_active_training1:is_untrained1          -0.030871242 0.16280833
## hu_is_post1:is_active_training1:is_untrained1 -0.483606828 0.22932951
##                                                      Q2.5        Q97.5
## Intercept                                      3.50945291  3.741106707
## hu_Intercept                                  -0.08993076  0.496640966
## is_post1                                      -0.27348560 -0.003489211
## is_active_training1                           -0.23303582  0.079325144
## is_untrained1                                 -0.17377234  0.082854767
## is_post1:is_active_training1                  -0.19652732  0.184337219
## is_post1:is_untrained1                        -0.20144235  0.179453538
## is_active_training1:is_untrained1             -0.04522140  0.325259477
## is_post1:is_active_training1:is_untrained1    -0.37128895  0.175171287
## hu_is_post1                                   -0.33875730  0.191614330
## hu_is_active_training1                        -0.26322939  0.404151128
## hu_is_untrained1                              -0.23591346  0.206562211
## hu_is_post1:is_active_training1               -0.31160774  0.435337442
## hu_is_post1:is_untrained1                      0.29429883  0.935632539
## hu_is_active_training1:is_untrained1          -0.35244185  0.279565080
## hu_is_post1:is_active_training1:is_untrained1 -0.92758697 -0.028183474
```

**Increase foods:**

```
## Approach Bias
```

```
## bias_stim ~ is_post * is_active_training * is_untrained + (is_post | vpNumber) + (is_post | imgNumber)
```

```
##                                              Estimate Est.Error      Q2.5
## Intercept                                   58.130487  15.90256  28.25652
## is_post1                                    -8.894672  21.05337 -49.67014
## is_active_training1                         12.734357  21.79897 -30.08751
## is_untrained1                               -2.990433   7.11710 -16.64062
## is_post1:is_active_training1                -7.427558  28.32107 -65.35866
## is_post1:is_untrained1                       9.245147  10.36028 -11.18981
## is_active_training1:is_untrained1           -6.316527  10.16477 -26.44211
## is_post1:is_active_training1:is_untrained1 -23.709341  14.79050 -52.59971
##                                                Q97.5
## Intercept                                  89.635432
## is_post1                                   31.809408
## is_active_training1                        55.825678
## is_untrained1                              10.664796
## is_post1:is_active_training1               46.928091
## is_post1:is_untrained1                     29.230289
## is_active_training1:is_untrained1          13.227751
## is_post1:is_active_training1:is_untrained1  5.286906
```

```
## Craving
```

```
## Craving ~ is_post * is_active_training * is_untrained + (is_post | vpNumber) + (is_post | imgNumber) 
## hu ~ is_post * is_active_training * is_untrained + (is_post | vpNumber) + (is_post | imgNumber)
```

```
##                                                   Estimate  Est.Error
## Intercept                                      3.601131618 0.05369646
## hu_Intercept                                  -0.627478699 0.20119495
## is_post1                                      -0.121286507 0.06390690
## is_active_training1                            0.021088967 0.07207476
## is_untrained1                                  0.076726194 0.04927947
## is_post1:is_active_training1                  -0.039962083 0.09169402
## is_post1:is_untrained1                        -0.050876628 0.07197653
## is_active_training1:is_untrained1             -0.069260377 0.07218138
## is_post1:is_active_training1:is_untrained1    -0.020277716 0.10391491
## hu_is_post1                                    0.089524925 0.18386708
## hu_is_active_training1                         0.278839471 0.26873701
## hu_is_untrained1                              -0.156810097 0.12817731
## hu_is_post1:is_active_training1               -0.277041601 0.24797619
## hu_is_post1:is_untrained1                      0.220117925 0.18216453
## hu_is_active_training1:is_untrained1           0.001909623 0.17987804
## hu_is_post1:is_active_training1:is_untrained1 -0.143731319 0.26372284
##                                                      Q2.5        Q97.5
## Intercept                                      3.49419754  3.704183451
## hu_Intercept                                  -1.01768729 -0.236100475
## is_post1                                      -0.24783929  0.004547384
## is_active_training1                           -0.11769085  0.160068656
## is_untrained1                                 -0.01623913  0.173559873
## is_post1:is_active_training1                  -0.22366965  0.139063257
## is_post1:is_untrained1                        -0.19243815  0.089297109
## is_active_training1:is_untrained1             -0.21039813  0.072905952
## is_post1:is_active_training1:is_untrained1    -0.21694640  0.188654328
## hu_is_post1                                   -0.27477205  0.458693544
## hu_is_active_training1                        -0.23524876  0.812500281
## hu_is_untrained1                              -0.41285265  0.095752997
## hu_is_post1:is_active_training1               -0.77297697  0.211534737
## hu_is_post1:is_untrained1                     -0.13616001  0.571086380
## hu_is_active_training1:is_untrained1          -0.34934560  0.350141100
## hu_is_post1:is_active_training1:is_untrained1 -0.65168444  0.369468914
```

```
## Intake
```

```
## Intake ~ is_post * is_active_training * is_untrained + (is_post | vpNumber) + (is_post | imgNumber) 
## hu ~ is_post * is_active_training * is_untrained + (is_post | vpNumber) + (is_post | imgNumber)
```

```
##                                                  Estimate  Est.Error
## Intercept                                      3.47820863 0.06408204
## hu_Intercept                                   0.65827895 0.18214379
## is_post1                                      -0.13482249 0.08609772
## is_active_training1                            0.18799470 0.07936883
## is_untrained1                                  0.12931770 0.07876043
## is_post1:is_active_training1                  -0.13047954 0.12203786
## is_post1:is_untrained1                        -0.08505253 0.11422507
## is_active_training1:is_untrained1             -0.21058017 0.11474853
## is_post1:is_active_training1:is_untrained1     0.04582177 0.16488371
## hu_is_post1                                   -0.09174916 0.14338909
## hu_is_active_training1                         0.06312969 0.21419871
## hu_is_untrained1                              -0.07833470 0.12290721
## hu_is_post1:is_active_training1               -0.10539390 0.20346172
## hu_is_post1:is_untrained1                      0.22833914 0.17833175
## hu_is_active_training1:is_untrained1           0.01621018 0.17852231
## hu_is_post1:is_active_training1:is_untrained1 -0.36897794 0.24849648
##                                                      Q2.5      Q97.5
## Intercept                                      3.34793645 3.59936116
## hu_Intercept                                   0.29373888 1.00533358
## is_post1                                      -0.30373679 0.03115301
## is_active_training1                            0.03161771 0.34411141
## is_untrained1                                 -0.02238616 0.29027166
## is_post1:is_active_training1                  -0.37126506 0.10619456
## is_post1:is_untrained1                        -0.31384938 0.13573399
## is_active_training1:is_untrained1             -0.43905455 0.01306080
## is_post1:is_active_training1:is_untrained1    -0.27889644 0.37127186
## hu_is_post1                                   -0.36958331 0.18613235
## hu_is_active_training1                        -0.36620981 0.48402370
## hu_is_untrained1                              -0.32444509 0.16691665
## hu_is_post1:is_active_training1               -0.51853829 0.28653674
## hu_is_post1:is_untrained1                     -0.11961881 0.56464428
## hu_is_active_training1:is_untrained1          -0.34423569 0.37198649
## hu_is_post1:is_active_training1:is_untrained1 -0.85512849 0.11917211
```

### Stimulus-specific baseline craving

**Decrease foods:**

```
## Approach Bias
```

```
## bias_stim ~ is_post * is_active_training * PreCraving + (is_post | vpNumber) + (is_post | imgNumber)
```

```
##                                             Estimate  Est.Error         Q2.5
## Intercept                                48.97472421 15.8036558  17.43493258
## is_post1                                  2.85108644 19.1629422 -35.01269974
## is_active_training1                      -5.24887935 22.2047799 -47.97250582
## PreCraving                                0.10452351  0.1782019  -0.25193938
## is_post1:is_active_training1            -34.04389768 26.0971765 -85.06587408
## is_post1:PreCraving                      -0.07300803  0.2473109  -0.55143775
## is_active_training1:PreCraving            0.49839807  0.2584274  -0.01085538
## is_post1:is_active_training1:PreCraving  -0.11230692  0.3587513  -0.79507326
##                                              Q97.5
## Intercept                               80.2517963
## is_post1                                39.8285913
## is_active_training1                     39.0067256
## PreCraving                               0.4511740
## is_post1:is_active_training1            17.1100821
## is_post1:PreCraving                      0.4090754
## is_active_training1:PreCraving           1.0027094
## is_post1:is_active_training1:PreCraving  0.6091587
```

```
## Intake
```

```
## Intake ~ is_post * is_active_training * PreCraving + (1 | vpNumber) + (1 | imgNumber) 
## hu ~ is_post * is_active_training * PreCraving + (1 | vpNumber) + (1 | imgNumber)
```

```
##                                                 Estimate   Est.Error
## Intercept                                   3.0271985335 0.078960929
## hu_Intercept                                1.1981858370 0.158606304
## is_post1                                    0.1660023988 0.086819728
## is_active_training1                         0.1049092304 0.110953565
## PreCraving                                  0.0137646192 0.001429784
## is_post1:is_active_training1               -0.0446876877 0.125625454
## is_post1:PreCraving                        -0.0060467352 0.001720425
## is_active_training1:PreCraving             -0.0019014313 0.002076902
## is_post1:is_active_training1:PreCraving    -0.0002283701 0.002605489
## hu_is_post1                                -0.1346836522 0.136673282
## hu_is_active_training1                     -0.0383597253 0.205481296
## hu_PreCraving                              -0.0319307377 0.002725808
## hu_is_post1:is_active_training1            -0.3701693542 0.187068985
## hu_is_post1:PreCraving                      0.0085669025 0.003185304
## hu_is_active_training1:PreCraving           0.0004067157 0.003832597
## hu_is_post1:is_active_training1:PreCraving  0.0092276445 0.004456599
##                                                     Q2.5        Q97.5
## Intercept                                   2.8743353485  3.181935835
## hu_Intercept                                0.8928865660  1.515153907
## is_post1                                   -0.0009475608  0.335101228
## is_active_training1                        -0.1060154036  0.327298412
## PreCraving                                  0.0110034901  0.016549962
## is_post1:is_active_training1               -0.2938479472  0.202178490
## is_post1:PreCraving                        -0.0095158566 -0.002706165
## is_active_training1:PreCraving             -0.0060058973  0.002120682
## is_post1:is_active_training1:PreCraving    -0.0053029177  0.004991410
## hu_is_post1                                -0.4038743623  0.130694069
## hu_is_active_training1                     -0.4421329588  0.358261804
## hu_PreCraving                              -0.0374128087 -0.026616655
## hu_is_post1:is_active_training1            -0.7400955579 -0.008459270
## hu_is_post1:PreCraving                      0.0024371000  0.014824192
## hu_is_active_training1:PreCraving          -0.0071158952  0.007965397
## hu_is_post1:is_active_training1:PreCraving  0.0004977236  0.018043827
```

**Increase foods:**

```
## Approach Bias
```

```
## bias_stim ~ is_post * is_active_training * PreCraving + (is_post | vpNumber) + (is_post | imgNumber)
```

```
##                                            Estimate  Est.Error        Q2.5
## Intercept                                61.4640986 16.3912202  28.1613580
## is_post1                                -14.0268435 22.1110947 -54.8473468
## is_active_training1                       6.1831181 22.7990840 -38.1977311
## PreCraving                               -0.1402043  0.1798973  -0.4876209
## is_post1:is_active_training1             -3.1550528 29.2233026 -59.8309379
## is_post1:PreCraving                       0.3068811  0.2681437  -0.2139344
## is_active_training1:PreCraving            0.1861246  0.2704807  -0.3411268
## is_post1:is_active_training1:PreCraving  -0.4555522  0.4009371  -1.2351279
##                                              Q97.5
## Intercept                               92.3809405
## is_post1                                30.4577787
## is_active_training1                     51.3503089
## PreCraving                               0.2153145
## is_post1:is_active_training1            54.8281986
## is_post1:PreCraving                      0.8219470
## is_active_training1:PreCraving           0.7147111
## is_post1:is_active_training1:PreCraving  0.3229831
```

```
## Intake
```

```
## Intake ~ is_post * is_active_training * PreCraving + (1 | vpNumber) + (1 | imgNumber) 
## hu ~ is_post * is_active_training * PreCraving + (1 | vpNumber) + (1 | imgNumber)
```

```
##                                                 Estimate   Est.Error
## Intercept                                   2.9305921373 0.090258817
## hu_Intercept                                1.5417995334 0.163060654
## is_post1                                    0.2335584695 0.100228678
## is_active_training1                         0.1062671441 0.126602637
## PreCraving                                  0.0160583467 0.001858627
## is_post1:is_active_training1               -0.0435461844 0.145133793
## is_post1:PreCraving                        -0.0079697225 0.002332951
## is_active_training1:PreCraving              0.0004773886 0.002763538
## is_post1:is_active_training1:PreCraving    -0.0012923833 0.003440207
## hu_is_post1                                -0.4899636699 0.127662676
## hu_is_active_training1                      0.2277475380 0.219889449
## hu_PreCraving                              -0.0360937664 0.003066948
## hu_is_post1:is_active_training1            -0.1683101921 0.186406639
## hu_is_post1:PreCraving                      0.0179795749 0.003621323
## hu_is_active_training1:PreCraving          -0.0103021935 0.004718606
## hu_is_post1:is_active_training1:PreCraving -0.0008884969 0.005567372
##                                                    Q2.5        Q97.5
## Intercept                                   2.751594482  3.109960683
## hu_Intercept                                1.216425346  1.858223739
## is_post1                                    0.033112911  0.431595799
## is_active_training1                        -0.139275930  0.359772747
## PreCraving                                  0.012397125  0.019652396
## is_post1:is_active_training1               -0.328972997  0.244348866
## is_post1:PreCraving                        -0.012479810 -0.003491385
## is_active_training1:PreCraving             -0.004759660  0.005927012
## is_post1:is_active_training1:PreCraving    -0.007994847  0.005390709
## hu_is_post1                                -0.732991960 -0.239559714
## hu_is_active_training1                     -0.200013503  0.648847603
## hu_PreCraving                              -0.042048307 -0.030139679
## hu_is_post1:is_active_training1            -0.535463350  0.195897941
## hu_is_post1:PreCraving                      0.010947037  0.025103556
## hu_is_active_training1:PreCraving          -0.019898160 -0.001170336
## hu_is_post1:is_active_training1:PreCraving -0.011400999  0.009916242
```

# Short-term analyses

## Short-term effects and moderation by group (training vs control group)

We further examined short-term effects of the training. Our
hypothesis was (H2 in the pre-registration): Relative to non-training
days, days with either an AAI or an AAA are characterized by less
cravings and intake of the decrease-foods and more cravings and intake
of the increase-foods. We also expected a larger difference between
training and non-training days in the training group than in the control
group. Some model convergence criteria indicate convergence issues.

**Decrease foods:**

```
## Craving
```

```
## Craving ~ has_aat_data * is_active_training + (has_aat_data | vpNumber) + (has_aat_data * is_active_training | imgNumber) + (has_aat_data * is_active_training | vpNumber:imgNumber) 
## hu ~ has_aat_data * is_active_training + (has_aat_data | vpNumber) + (has_aat_data * is_active_training | imgNumber) + (has_aat_data * is_active_training | vpNumber:imgNumber)
```

```
##                                         Estimate  Est.Error        Q2.5
## Intercept                             3.55770284 0.05652908  3.44783111
## hu_Intercept                         -0.84849764 0.24026137 -1.31651820
## has_aat_data1                         0.01295958 0.02658629 -0.03839482
## is_active_training1                  -0.14010297 0.07619653 -0.28784545
## has_aat_data1:is_active_training1     0.03460295 0.04020435 -0.04296168
## hu_has_aat_data1                     -0.14901028 0.08936465 -0.32960730
## hu_is_active_training1                0.15163531 0.31272667 -0.45835725
## hu_has_aat_data1:is_active_training1  0.06125191 0.12267403 -0.17742901
##                                            Q97.5
## Intercept                             3.66869397
## hu_Intercept                         -0.36649647
## has_aat_data1                         0.06493023
## is_active_training1                   0.01075173
## has_aat_data1:is_active_training1     0.11376788
## hu_has_aat_data1                      0.02228399
## hu_is_active_training1                0.77605776
## hu_has_aat_data1:is_active_training1  0.29933863
```

```
## Intake
```

```
## Intake ~ has_aat_data * is_active_training + (has_aat_data | vpNumber) + (has_aat_data * is_active_training | imgNumber) + (has_aat_data * is_active_training | vpNumber:imgNumber) 
## hu ~ has_aat_data * is_active_training + (has_aat_data | vpNumber) + (has_aat_data * is_active_training | imgNumber) + (has_aat_data * is_active_training | vpNumber:imgNumber)
```

```
##                                         Estimate  Est.Error        Q2.5
## Intercept                             3.38226820 0.06642481  3.25186881
## hu_Intercept                          0.40901544 0.15602871  0.10482284
## has_aat_data1                         0.04919158 0.03700676 -0.02241706
## is_active_training1                  -0.01205351 0.08434880 -0.17414872
## has_aat_data1:is_active_training1    -0.03480850 0.05662481 -0.14523129
## hu_has_aat_data1                     -0.02354074 0.08514129 -0.18920682
## hu_is_active_training1                0.01801792 0.18807627 -0.35085344
## hu_has_aat_data1:is_active_training1  0.07222807 0.12069603 -0.16205109
##                                           Q97.5
## Intercept                            3.51216019
## hu_Intercept                         0.71667222
## has_aat_data1                        0.12237920
## is_active_training1                  0.15515565
## has_aat_data1:is_active_training1    0.07716466
## hu_has_aat_data1                     0.14480542
## hu_is_active_training1               0.39047858
## hu_has_aat_data1:is_active_training1 0.31381939
```

**Increase foods:**

```
## Craving
```

```
## Craving ~ has_aat_data * is_active_training + (has_aat_data | vpNumber) + (has_aat_data * is_active_training | imgNumber) + (has_aat_data * is_active_training | vpNumber:imgNumber) 
## hu ~ has_aat_data * is_active_training + (has_aat_data | vpNumber) + (has_aat_data * is_active_training | imgNumber) + (has_aat_data * is_active_training | vpNumber:imgNumber)
```

```
##                                         Estimate  Est.Error       Q2.5
## Intercept                             3.43082508 0.06478541  3.3049998
## hu_Intercept                         -0.51586522 0.26432736 -1.0333436
## has_aat_data1                         0.02182668 0.02800832 -0.0331567
## is_active_training1                  -0.01119738 0.09188293 -0.1919334
## has_aat_data1:is_active_training1    -0.03358170 0.03942985 -0.1108388
## hu_has_aat_data1                     -0.16728568 0.09865844 -0.3615789
## hu_is_active_training1               -0.14558725 0.37313130 -0.8932923
## hu_has_aat_data1:is_active_training1  0.08185121 0.14215724 -0.1972484
##                                             Q97.5
## Intercept                             3.558823951
## hu_Intercept                         -0.004319062
## has_aat_data1                         0.076381382
## is_active_training1                   0.168174775
## has_aat_data1:is_active_training1     0.043185235
## hu_has_aat_data1                      0.027668584
## hu_is_active_training1                0.573223677
## hu_has_aat_data1:is_active_training1  0.364768765
```

```
## Intake
```

```
## Intake ~ has_aat_data * is_active_training + (has_aat_data | vpNumber) + (has_aat_data * is_active_training | imgNumber) + (has_aat_data * is_active_training | vpNumber:imgNumber) 
## hu ~ has_aat_data * is_active_training + (has_aat_data | vpNumber) + (has_aat_data * is_active_training | imgNumber) + (has_aat_data * is_active_training | vpNumber:imgNumber)
```

```
##                                         Estimate  Est.Error        Q2.5
## Intercept                             3.35913424 0.07967886  3.20230670
## hu_Intercept                          0.66869183 0.17369011  0.32606719
## has_aat_data1                        -0.03150994 0.04773802 -0.12623283
## is_active_training1                  -0.03175403 0.10548251 -0.24012703
## has_aat_data1:is_active_training1     0.04952167 0.06645025 -0.08261812
## hu_has_aat_data1                     -0.02530700 0.07532644 -0.17443404
## hu_is_active_training1               -0.18375497 0.23375296 -0.64356004
## hu_has_aat_data1:is_active_training1 -0.10146532 0.11012079 -0.31637201
##                                           Q97.5
## Intercept                            3.51513631
## hu_Intercept                         1.01239438
## has_aat_data1                        0.06152145
## is_active_training1                  0.17479365
## has_aat_data1:is_active_training1    0.17873839
## hu_has_aat_data1                     0.12323859
## hu_is_active_training1               0.27787375
## hu_has_aat_data1:is_active_training1 0.11617696
```

## Moderation by training status of the stimulus

We expected a larger difference between training and non-training
days for trained than for untrained stimuli. Some model convergence
criteria indicate convergence issues.

**Decrease foods:**

```
## Craving
```

```
## Craving ~ has_aat_data * is_active_training * is_untrained + (has_aat_data * is_untrained | vpNumber) + (has_aat_data * is_active_training * is_untrained | imgNumber) + (has_aat_data * is_active_training * is_untrained | vpNumber:imgNumber) 
## hu ~ has_aat_data * is_active_training * is_untrained + (has_aat_data * is_untrained | vpNumber) + (has_aat_data * is_active_training * is_untrained | imgNumber) + (has_aat_data * is_active_training * is_untrained | vpNumber:imgNumber)
```

```
##                                                        Estimate  Est.Error
## Intercept                                           3.573745843 0.05861512
## hu_Intercept                                       -0.936427033 0.24540457
## has_aat_data1                                      -0.005109862 0.03144255
## is_active_training1                                -0.195075781 0.07983772
## is_untrained1                                      -0.073139281 0.05742326
## has_aat_data1:is_active_training1                   0.045009545 0.04837001
## has_aat_data1:is_untrained1                         0.054207792 0.05555110
## is_active_training1:is_untrained1                   0.171439880 0.08442295
## has_aat_data1:is_active_training1:is_untrained1    -0.027346962 0.08442058
## hu_has_aat_data1                                   -0.159973393 0.10277397
## hu_is_active_training1                              0.246147861 0.33060208
## hu_is_untrained1                                    0.240246704 0.18529139
## hu_has_aat_data1:is_active_training1                0.130160366 0.14385258
## hu_has_aat_data1:is_untrained1                      0.059844907 0.17741309
## hu_is_active_training1:is_untrained1               -0.310713948 0.26830122
## hu_has_aat_data1:is_active_training1:is_untrained1 -0.249156800 0.25555494
##                                                            Q2.5       Q97.5
## Intercept                                           3.459337355  3.68742792
## hu_Intercept                                       -1.421583095 -0.45135483
## has_aat_data1                                      -0.068211068  0.05517150
## is_active_training1                                -0.351903311 -0.03785763
## is_untrained1                                      -0.188024958  0.03642173
## has_aat_data1:is_active_training1                  -0.046781739  0.14031173
## has_aat_data1:is_untrained1                        -0.052235441  0.16521612
## is_active_training1:is_untrained1                   0.006718549  0.33699368
## has_aat_data1:is_active_training1:is_untrained1    -0.191523733  0.13926174
## hu_has_aat_data1                                   -0.367001647  0.04391989
## hu_is_active_training1                             -0.414576574  0.90080536
## hu_is_untrained1                                   -0.123461695  0.59857179
## hu_has_aat_data1:is_active_training1               -0.149240704  0.41105656
## hu_has_aat_data1:is_untrained1                     -0.280260964  0.40860209
## hu_is_active_training1:is_untrained1               -0.836786158  0.20423811
## hu_has_aat_data1:is_active_training1:is_untrained1 -0.754756137  0.24403431
```

```
## Intake
```

```
## Intake ~ has_aat_data * is_active_training * is_untrained + (has_aat_data * is_untrained | vpNumber) + (has_aat_data * is_active_training * is_untrained | imgNumber) + (has_aat_data * is_active_training * is_untrained | vpNumber:imgNumber) 
## hu ~ has_aat_data * is_active_training * is_untrained + (has_aat_data * is_untrained | vpNumber) + (has_aat_data * is_active_training * is_untrained | imgNumber) + (has_aat_data * is_active_training * is_untrained | vpNumber:imgNumber)
```

```
##                                                        Estimate  Est.Error
## Intercept                                           3.389941204 0.06865314
## hu_Intercept                                        0.316291047 0.16179576
## has_aat_data1                                       0.032181348 0.04550152
## is_active_training1                                -0.058036011 0.09228867
## is_untrained1                                      -0.051111415 0.07240391
## has_aat_data1:is_active_training1                  -0.044652364 0.06635034
## has_aat_data1:is_untrained1                         0.058366051 0.08252447
## is_active_training1:is_untrained1                   0.152453755 0.10328812
## has_aat_data1:is_active_training1:is_untrained1     0.027647355 0.11932542
## hu_has_aat_data1                                   -0.019599731 0.09559706
## hu_is_active_training1                              0.104140405 0.20136311
## hu_is_untrained1                                    0.311880603 0.16150357
## hu_has_aat_data1:is_active_training1                0.071922369 0.13370957
## hu_has_aat_data1:is_untrained1                     -0.023573531 0.15854461
## hu_is_active_training1:is_untrained1               -0.281237041 0.21500148
## hu_has_aat_data1:is_active_training1:is_untrained1 -0.006856027 0.23333695
##                                                            Q2.5      Q97.5
## Intercept                                           3.255952078 3.52265360
## hu_Intercept                                       -0.008875342 0.62522262
## has_aat_data1                                      -0.054420740 0.12403453
## is_active_training1                                -0.232364641 0.12230236
## is_untrained1                                      -0.192430242 0.09071452
## has_aat_data1:is_active_training1                  -0.177018660 0.08406765
## has_aat_data1:is_untrained1                        -0.105374924 0.22059593
## is_active_training1:is_untrained1                  -0.047849194 0.35785786
## has_aat_data1:is_active_training1:is_untrained1    -0.206111989 0.26099054
## hu_has_aat_data1                                   -0.204406432 0.16909634
## hu_is_active_training1                             -0.293311148 0.48408287
## hu_is_untrained1                                   -0.008051626 0.63455533
## hu_has_aat_data1:is_active_training1               -0.182092749 0.33661919
## hu_has_aat_data1:is_untrained1                     -0.335249524 0.28692848
## hu_is_active_training1:is_untrained1               -0.719395873 0.13943854
## hu_has_aat_data1:is_active_training1:is_untrained1 -0.458539275 0.43797747
```

**Increase foods:**

```
## Craving
```

```
## Craving ~ has_aat_data * is_active_training * is_untrained + (has_aat_data * is_untrained | vpNumber) + (has_aat_data * is_active_training * is_untrained | imgNumber) + (has_aat_data * is_active_training * is_untrained | vpNumber:imgNumber) 
## hu ~ has_aat_data * is_active_training * is_untrained + (has_aat_data * is_untrained | vpNumber) + (has_aat_data * is_active_training * is_untrained | imgNumber) + (has_aat_data * is_active_training * is_untrained | vpNumber:imgNumber)
```

```
##                                                         Estimate  Est.Error
## Intercept                                           3.4122200046 0.06803614
## hu_Intercept                                       -0.5360400903 0.26827683
## has_aat_data1                                       0.0413241222 0.03358158
## is_active_training1                                -0.0002806958 0.09788102
## is_untrained1                                       0.0558440161 0.05995369
## has_aat_data1:is_active_training1                  -0.0281147899 0.04730233
## has_aat_data1:is_untrained1                        -0.0645297659 0.05983680
## is_active_training1:is_untrained1                  -0.0359620760 0.08336097
## has_aat_data1:is_active_training1:is_untrained1    -0.0196853024 0.08415945
## hu_has_aat_data1                                   -0.1288460849 0.10844569
## hu_is_active_training1                             -0.2199201440 0.37601565
## hu_is_untrained1                                    0.0459701657 0.17417781
## hu_has_aat_data1:is_active_training1                0.0771300379 0.15726179
## hu_has_aat_data1:is_untrained1                     -0.1219345003 0.17031708
## hu_is_active_training1:is_untrained1                0.2334703563 0.25973962
## hu_has_aat_data1:is_active_training1:is_untrained1  0.0173201145 0.25343925
##                                                           Q2.5       Q97.5
## Intercept                                           3.27794027  3.54261983
## hu_Intercept                                       -1.08179966 -0.02035296
## has_aat_data1                                      -0.02495733  0.10700451
## is_active_training1                                -0.19249917  0.19366839
## is_untrained1                                      -0.06278688  0.17108252
## has_aat_data1:is_active_training1                  -0.12273418  0.06372322
## has_aat_data1:is_untrained1                        -0.18002595  0.05415031
## is_active_training1:is_untrained1                  -0.20331704  0.12500051
## has_aat_data1:is_active_training1:is_untrained1    -0.18496082  0.14631011
## hu_has_aat_data1                                   -0.34386413  0.08451198
## hu_is_active_training1                             -0.95077357  0.51509388
## hu_is_untrained1                                   -0.29352140  0.38659966
## hu_has_aat_data1:is_active_training1               -0.22863559  0.38775799
## hu_has_aat_data1:is_untrained1                     -0.45792710  0.21017752
## hu_is_active_training1:is_untrained1               -0.26813031  0.74349991
## hu_has_aat_data1:is_active_training1:is_untrained1 -0.46818602  0.50819848
```

```
## Intake
```

```
## Intake ~ has_aat_data * is_active_training * is_untrained + (has_aat_data * is_untrained | vpNumber) + (has_aat_data * is_active_training * is_untrained | imgNumber) + (has_aat_data * is_active_training * is_untrained | vpNumber:imgNumber) 
## hu ~ has_aat_data * is_active_training * is_untrained + (has_aat_data * is_untrained | vpNumber) + (has_aat_data * is_active_training * is_untrained | imgNumber) + (has_aat_data * is_active_training * is_untrained | vpNumber:imgNumber)
```

```
##                                                        Estimate  Est.Error
## Intercept                                           3.372163928 0.08393365
## hu_Intercept                                        0.689835732 0.17535871
## has_aat_data1                                      -0.037035463 0.05807719
## is_active_training1                                -0.080328808 0.11064515
## is_untrained1                                      -0.021000570 0.08347708
## has_aat_data1:is_active_training1                   0.095774980 0.08011004
## has_aat_data1:is_untrained1                         0.002914976 0.09399141
## is_active_training1:is_untrained1                   0.099272700 0.11529540
## has_aat_data1:is_active_training1:is_untrained1    -0.138888809 0.13727867
## hu_has_aat_data1                                   -0.020985706 0.08851281
## hu_is_active_training1                             -0.214139020 0.23532611
## hu_is_untrained1                                   -0.060389777 0.14291343
## hu_has_aat_data1:is_active_training1               -0.134476938 0.12948601
## hu_has_aat_data1:is_untrained1                     -0.003383225 0.14753011
## hu_is_active_training1:is_untrained1                0.128610473 0.20903711
## hu_has_aat_data1:is_active_training1:is_untrained1  0.086595848 0.21622742
##                                                           Q2.5      Q97.5
## Intercept                                           3.20744195 3.53278310
## hu_Intercept                                        0.35653941 1.03400183
## has_aat_data1                                      -0.14993350 0.07773354
## is_active_training1                                -0.30484167 0.13821064
## is_untrained1                                      -0.18731712 0.14219712
## has_aat_data1:is_active_training1                  -0.06431952 0.24976367
## has_aat_data1:is_untrained1                        -0.18169905 0.18511000
## is_active_training1:is_untrained1                  -0.12688073 0.32796699
## has_aat_data1:is_active_training1:is_untrained1    -0.40866216 0.12712709
## hu_has_aat_data1                                   -0.19564536 0.14999817
## hu_is_active_training1                             -0.67047116 0.25495667
## hu_is_untrained1                                   -0.33729640 0.21889060
## hu_has_aat_data1:is_active_training1               -0.38995021 0.12323990
## hu_has_aat_data1:is_untrained1                     -0.29697157 0.27964315
## hu_is_active_training1:is_untrained1               -0.28265512 0.53912705
## hu_has_aat_data1:is_active_training1:is_untrained1 -0.33836782 0.50491473
```

## Moderation by impulsivity

We expected that the difference between training and non-training
days is moderated by baseline impulsivity scores (measured with the
UPPS)

**Decrease foods:**

```
## Craving - Subscale: UPPS Perseverance
```

```
## Craving ~ has_aat_data * is_active_training * UPPSpers + (has_aat_data | vpNumber) + (has_aat_data * is_active_training | imgNumber) + (has_aat_data | vpNumber:imgNumber) 
## hu ~ has_aat_data * is_active_training * UPPSpers + (has_aat_data | vpNumber) + (has_aat_data * is_active_training | imgNumber) + (has_aat_data | vpNumber:imgNumber)
```

```
##                                                   Estimate  Est.Error
## Intercept                                      3.922128688 0.23974676
## hu_Intercept                                  -1.843385564 0.99922665
## has_aat_data1                                 -0.013921161 0.11996595
## is_active_training1                           -0.707967705 0.37868141
## UPPSpers                                      -0.126719672 0.08024388
## has_aat_data1:is_active_training1             -0.184375333 0.19701594
## has_aat_data1:UPPSpers                         0.009539376 0.04075615
## is_active_training1:UPPSpers                   0.194755043 0.12364063
## has_aat_data1:is_active_training1:UPPSpers     0.071146609 0.06473848
## hu_has_aat_data1                              -0.195849483 0.37960128
## hu_is_active_training1                         1.068512812 1.58192374
## hu_UPPSpers                                    0.346139214 0.33452531
## hu_has_aat_data1:is_active_training1          -0.820314550 0.58697591
## hu_has_aat_data1:UPPSpers                      0.015776529 0.12517700
## hu_is_active_training1:UPPSpers               -0.323418836 0.51784612
## hu_has_aat_data1:is_active_training1:UPPSpers  0.288906296 0.19300389
##                                                      Q2.5      Q97.5
## Intercept                                      3.46189718 4.40057899
## hu_Intercept                                  -3.80444493 0.13794089
## has_aat_data1                                 -0.24374899 0.22469204
## is_active_training1                           -1.44669890 0.04179765
## UPPSpers                                      -0.29063325 0.02673258
## has_aat_data1:is_active_training1             -0.56735150 0.20317316
## has_aat_data1:UPPSpers                        -0.06936016 0.08839762
## is_active_training1:UPPSpers                  -0.04667954 0.43043937
## has_aat_data1:is_active_training1:UPPSpers    -0.05685091 0.19984808
## hu_has_aat_data1                              -0.94521373 0.54722388
## hu_is_active_training1                        -2.05238804 4.05676670
## hu_UPPSpers                                   -0.33316140 0.98929285
## hu_has_aat_data1:is_active_training1          -1.97032806 0.36741133
## hu_has_aat_data1:UPPSpers                     -0.22812040 0.25703820
## hu_is_active_training1:UPPSpers               -1.31139514 0.68741523
## hu_has_aat_data1:is_active_training1:UPPSpers -0.10034701 0.65802017
```

```
## Craving - Subscale: UPPS Premeditation
```

```
## Craving ~ has_aat_data * is_active_training * UPPSprem + (has_aat_data | vpNumber) + (has_aat_data * is_active_training | imgNumber) + (has_aat_data | vpNumber:imgNumber) 
## hu ~ has_aat_data * is_active_training * UPPSprem + (has_aat_data | vpNumber) + (has_aat_data * is_active_training | imgNumber) + (has_aat_data | vpNumber:imgNumber)
```

```
##                                                  Estimate  Est.Error
## Intercept                                      3.86723576 0.28255596
## hu_Intercept                                  -0.14485017 1.15855328
## has_aat_data1                                  0.19988622 0.15150563
## is_active_training1                           -1.11887413 0.41811677
## UPPSprem                                      -0.10314665 0.09332850
## has_aat_data1:is_active_training1             -0.23596620 0.22788091
## has_aat_data1:UPPSprem                        -0.06225744 0.04945747
## is_active_training1:UPPSprem                   0.32379689 0.13617647
## has_aat_data1:is_active_training1:UPPSprem     0.08967517 0.07340500
## hu_has_aat_data1                              -0.11351010 0.43027617
## hu_is_active_training1                         0.72909956 1.71117663
## hu_UPPSprem                                   -0.23274466 0.38164302
## hu_has_aat_data1:is_active_training1          -0.38481208 0.65630321
## hu_has_aat_data1:UPPSprem                     -0.01224868 0.14010006
## hu_is_active_training1:UPPSprem               -0.18938646 0.55968103
## hu_has_aat_data1:is_active_training1:UPPSprem  0.14492532 0.21231477
##                                                      Q2.5       Q97.5
## Intercept                                      3.30526183  4.42204524
## hu_Intercept                                  -2.37928724  2.09543680
## has_aat_data1                                 -0.08838812  0.50166310
## is_active_training1                           -1.93658134 -0.29614735
## UPPSprem                                      -0.28955235  0.07998673
## has_aat_data1:is_active_training1             -0.67212786  0.20021311
## has_aat_data1:UPPSprem                        -0.15921443  0.03196067
## is_active_training1:UPPSprem                   0.04697845  0.58850918
## has_aat_data1:is_active_training1:UPPSprem    -0.05277827  0.23322180
## hu_has_aat_data1                              -0.97474435  0.72156653
## hu_is_active_training1                        -2.66808598  4.07242112
## hu_UPPSprem                                   -0.98604841  0.50305319
## hu_has_aat_data1:is_active_training1          -1.68309305  0.89946146
## hu_has_aat_data1:UPPSprem                     -0.28542108  0.26170020
## hu_is_active_training1:UPPSprem               -1.29852150  0.92337413
## hu_has_aat_data1:is_active_training1:UPPSprem -0.26812247  0.55811018
```

```
## Craving - Subscale: UPPS Sensation Seeking
```

```
## Craving ~ has_aat_data * is_active_training * UPPSsen + (has_aat_data | vpNumber) + (has_aat_data * is_active_training | imgNumber) + (has_aat_data | vpNumber:imgNumber) 
## hu ~ has_aat_data * is_active_training * UPPSsen + (has_aat_data | vpNumber) + (has_aat_data * is_active_training | imgNumber) + (has_aat_data | vpNumber:imgNumber)
```

```
##                                                 Estimate  Est.Error        Q2.5
## Intercept                                     3.20980966 0.17367304  2.88146245
## hu_Intercept                                 -0.27086621 0.76719387 -1.82046676
## has_aat_data1                                 0.06803421 0.08361741 -0.09490918
## is_active_training1                           0.30933960 0.28213157 -0.27453446
## UPPSsen                                       0.14784941 0.06971358  0.01067047
## has_aat_data1:is_active_training1             0.11164716 0.14144669 -0.16530665
## has_aat_data1:UPPSsen                        -0.02328250 0.03417526 -0.08979313
## is_active_training1:UPPSsen                  -0.18620705 0.10973151 -0.39629102
## has_aat_data1:is_active_training1:UPPSsen    -0.02847963 0.05542727 -0.13481091
## hu_has_aat_data1                             -0.20558739 0.28152460 -0.76705374
## hu_is_active_training1                        0.27133525 1.20337527 -2.19533106
## hu_UPPSsen                                   -0.24714412 0.30947345 -0.86098794
## hu_has_aat_data1:is_active_training1          0.30608171 0.42998492 -0.53450640
## hu_has_aat_data1:UPPSsen                      0.02424217 0.11518128 -0.19873062
## hu_is_active_training1:UPPSsen               -0.02873055 0.47423995 -0.94870510
## hu_has_aat_data1:is_active_training1:UPPSsen -0.10142161 0.17106691 -0.43717433
##                                                   Q97.5
## Intercept                                    3.56297891
## hu_Intercept                                 1.28529485
## has_aat_data1                                0.23551742
## is_active_training1                          0.83343194
## UPPSsen                                      0.28386211
## has_aat_data1:is_active_training1            0.39010086
## has_aat_data1:UPPSsen                        0.04211784
## is_active_training1:UPPSsen                  0.03457746
## has_aat_data1:is_active_training1:UPPSsen    0.08159798
## hu_has_aat_data1                             0.33278585
## hu_is_active_training1                       2.58814814
## hu_UPPSsen                                   0.36961621
## hu_has_aat_data1:is_active_training1         1.14429450
## hu_has_aat_data1:UPPSsen                     0.24820942
## hu_is_active_training1:UPPSsen               0.92710170
## hu_has_aat_data1:is_active_training1:UPPSsen 0.23090604
```

```
## Craving - Subscale: UPPS Urgency
```

```
## Craving ~ has_aat_data * is_active_training * UPPSurg + (has_aat_data | vpNumber) + (has_aat_data * is_active_training | imgNumber) + (has_aat_data | vpNumber:imgNumber) 
## hu ~ has_aat_data * is_active_training * UPPSurg + (has_aat_data | vpNumber) + (has_aat_data * is_active_training | imgNumber) + (has_aat_data | vpNumber:imgNumber)
```

```
##                                                  Estimate  Est.Error
## Intercept                                     3.256014958 0.22831979
## hu_Intercept                                 -0.005707648 0.98123280
## has_aat_data1                                 0.197671085 0.11259332
## is_active_training1                           0.228414468 0.31529449
## UPPSurg                                       0.131233075 0.09689644
## has_aat_data1:is_active_training1            -0.119189373 0.15742892
## has_aat_data1:UPPSurg                        -0.081554737 0.04802319
## is_active_training1:UPPSurg                  -0.160734844 0.13678893
## has_aat_data1:is_active_training1:UPPSurg     0.067394577 0.06833873
## hu_has_aat_data1                             -0.029903778 0.33769491
## hu_is_active_training1                       -0.702769492 1.34071908
## hu_UPPSurg                                   -0.366709671 0.41298305
## hu_has_aat_data1:is_active_training1         -0.115823736 0.48901321
## hu_has_aat_data1:UPPSurg                     -0.053616346 0.14689280
## hu_is_active_training1:UPPSurg                0.375272307 0.57732236
## hu_has_aat_data1:is_active_training1:UPPSurg  0.076180627 0.21159696
##                                                     Q2.5      Q97.5
## Intercept                                     2.79886936 3.70428541
## hu_Intercept                                 -1.93625672 1.89294752
## has_aat_data1                                -0.02122130 0.41891678
## is_active_training1                          -0.40658706 0.85789476
## UPPSurg                                      -0.06081562 0.32528675
## has_aat_data1:is_active_training1            -0.42251243 0.19012078
## has_aat_data1:UPPSurg                        -0.17537573 0.01345152
## is_active_training1:UPPSurg                  -0.43741634 0.10985496
## has_aat_data1:is_active_training1:UPPSurg    -0.06699485 0.20115175
## hu_has_aat_data1                             -0.67767896 0.63556371
## hu_is_active_training1                       -3.36645438 1.99092398
## hu_UPPSurg                                   -1.17611750 0.42908523
## hu_has_aat_data1:is_active_training1         -1.10377273 0.84388377
## hu_has_aat_data1:UPPSurg                     -0.34375467 0.23674243
## hu_is_active_training1:UPPSurg               -0.77438178 1.50563893
## hu_has_aat_data1:is_active_training1:UPPSurg -0.32924132 0.49183585
```

```
## Intake - Subscale: UPPS Perseverance
```

```
## Intake ~ has_aat_data * is_active_training * UPPSpers + (has_aat_data | vpNumber) + (has_aat_data * is_active_training | imgNumber) + (has_aat_data | vpNumber:imgNumber) 
## hu ~ has_aat_data * is_active_training * UPPSpers + (has_aat_data | vpNumber) + (has_aat_data * is_active_training | imgNumber) + (has_aat_data | vpNumber:imgNumber)
```

```
##                                                  Estimate  Est.Error
## Intercept                                      3.96455402 0.25810604
## hu_Intercept                                   0.25169476 0.58415447
## has_aat_data1                                 -0.23195706 0.15432371
## is_active_training1                           -0.60615295 0.41139808
## UPPSpers                                      -0.20188641 0.08608302
## has_aat_data1:is_active_training1              0.14230891 0.27098664
## has_aat_data1:UPPSpers                         0.09777351 0.05256470
## is_active_training1:UPPSpers                   0.20474254 0.13523481
## has_aat_data1:is_active_training1:UPPSpers    -0.06242334 0.08892183
## hu_has_aat_data1                              -0.71611028 0.36811556
## hu_is_active_training1                        -0.10744817 0.92281689
## hu_UPPSpers                                    0.05224368 0.19545868
## hu_has_aat_data1:is_active_training1           0.34701113 0.58118738
## hu_has_aat_data1:UPPSpers                      0.23836127 0.12290224
## hu_is_active_training1:UPPSpers                0.04071908 0.30298643
## hu_has_aat_data1:is_active_training1:UPPSpers -0.10114143 0.19012687
##                                                       Q2.5        Q97.5
## Intercept                                      3.441113864  4.445087903
## hu_Intercept                                  -0.870560476  1.439343471
## has_aat_data1                                 -0.529096617  0.076380963
## is_active_training1                           -1.400401523  0.205678047
## UPPSpers                                      -0.369277341 -0.030733702
## has_aat_data1:is_active_training1             -0.406945997  0.666980296
## has_aat_data1:UPPSpers                        -0.007917030  0.198941969
## is_active_training1:UPPSpers                  -0.067154038  0.468887147
## has_aat_data1:is_active_training1:UPPSpers    -0.233415559  0.118694445
## hu_has_aat_data1                              -1.453984717 -0.009038802
## hu_is_active_training1                        -1.927250950  1.648174307
## hu_UPPSpers                                   -0.334760851  0.427439514
## hu_has_aat_data1:is_active_training1          -0.778303651  1.490865214
## hu_has_aat_data1:UPPSpers                      0.006499278  0.484689615
## hu_is_active_training1:UPPSpers               -0.533751930  0.652050775
## hu_has_aat_data1:is_active_training1:UPPSpers -0.481099308  0.267601557
```

```
## Intake - Subscale: UPPS Premeditation
```

```
## Intake ~ has_aat_data * is_active_training * UPPSprem + (has_aat_data | vpNumber) + (has_aat_data * is_active_training | imgNumber) + (has_aat_data | vpNumber:imgNumber) 
## hu ~ has_aat_data * is_active_training * UPPSprem + (has_aat_data | vpNumber) + (has_aat_data * is_active_training | imgNumber) + (has_aat_data | vpNumber:imgNumber)
```

```
##                                                  Estimate  Est.Error
## Intercept                                      4.15453918 0.31529803
## hu_Intercept                                   0.27625521 0.73028767
## has_aat_data1                                 -0.04867685 0.20473502
## is_active_training1                           -1.30418629 0.47147079
## UPPSprem                                      -0.25740857 0.10317676
## has_aat_data1:is_active_training1              0.04797340 0.30672144
## has_aat_data1:UPPSprem                         0.03280471 0.06731312
## is_active_training1:UPPSprem                   0.42723633 0.15400662
## has_aat_data1:is_active_training1:UPPSprem    -0.02653041 0.10004740
## hu_has_aat_data1                               0.07930566 0.46347029
## hu_is_active_training1                        -0.33440131 1.08663352
## hu_UPPSprem                                    0.04652099 0.23945307
## hu_has_aat_data1:is_active_training1          -0.29023213 0.67155984
## hu_has_aat_data1:UPPSprem                     -0.03443064 0.15205567
## hu_is_active_training1:UPPSprem                0.11379785 0.35309914
## hu_has_aat_data1:is_active_training1:UPPSprem  0.11936835 0.21799153
##                                                      Q2.5       Q97.5
## Intercept                                      3.53385667  4.79284825
## hu_Intercept                                  -1.07870835  1.76641258
## has_aat_data1                                 -0.45106076  0.35105630
## is_active_training1                           -2.23299392 -0.39400164
## UPPSprem                                      -0.46270788 -0.05391984
## has_aat_data1:is_active_training1             -0.54479059  0.66113500
## has_aat_data1:UPPSprem                        -0.09929505  0.16574927
## is_active_training1:UPPSprem                   0.12412074  0.72521309
## has_aat_data1:is_active_training1:UPPSprem    -0.22701204  0.16467669
## hu_has_aat_data1                              -0.82552157  0.98991590
## hu_is_active_training1                        -2.50061682  1.72017600
## hu_UPPSprem                                   -0.44242503  0.48846116
## hu_has_aat_data1:is_active_training1          -1.63602796  1.01258298
## hu_has_aat_data1:UPPSprem                     -0.32763488  0.26415318
## hu_is_active_training1:UPPSprem               -0.55985174  0.81870120
## hu_has_aat_data1:is_active_training1:UPPSprem -0.29851444  0.55482123
```

```
## Intake - Subscale: UPPS Sensation Seeking
```

```
## Intake ~ has_aat_data * is_active_training * UPPSsen + (has_aat_data | vpNumber) + (has_aat_data * is_active_training | imgNumber) + (has_aat_data | vpNumber:imgNumber) 
## hu ~ has_aat_data * is_active_training * UPPSsen + (has_aat_data | vpNumber) + (has_aat_data * is_active_training | imgNumber) + (has_aat_data | vpNumber:imgNumber)
```

```
##                                                  Estimate  Est.Error
## Intercept                                     3.129110186 0.19117009
## hu_Intercept                                  0.259673411 0.44979079
## has_aat_data1                                -0.009781282 0.11787807
## is_active_training1                           0.497700762 0.30028929
## UPPSsen                                       0.108481766 0.07691593
## has_aat_data1:is_active_training1             0.118944107 0.20351106
## has_aat_data1:UPPSsen                         0.024892084 0.04808633
## is_active_training1:UPPSsen                  -0.211262438 0.11898427
## has_aat_data1:is_active_training1:UPPSsen    -0.062189091 0.08025309
## hu_has_aat_data1                             -0.145750683 0.27074408
## hu_is_active_training1                       -0.254083128 0.68925806
## hu_UPPSsen                                    0.063476214 0.17766638
## hu_has_aat_data1:is_active_training1          0.005827560 0.43666115
## hu_has_aat_data1:UPPSsen                      0.052321419 0.11044556
## hu_is_active_training1:UPPSsen                0.101503148 0.27002251
## hu_has_aat_data1:is_active_training1:UPPSsen  0.023432213 0.17178412
##                                                     Q2.5      Q97.5
## Intercept                                     2.76833116 3.51173298
## hu_Intercept                                 -0.60668234 1.13183496
## has_aat_data1                                -0.24250493 0.21868271
## is_active_training1                          -0.08756716 1.08267003
## UPPSsen                                      -0.04659020 0.25072719
## has_aat_data1:is_active_training1            -0.28320153 0.50803266
## has_aat_data1:UPPSsen                        -0.06809487 0.12127978
## is_active_training1:UPPSsen                  -0.44191655 0.02479635
## has_aat_data1:is_active_training1:UPPSsen    -0.21446208 0.09498931
## hu_has_aat_data1                             -0.67899453 0.37751842
## hu_is_active_training1                       -1.57610002 1.10457066
## hu_UPPSsen                                   -0.27895072 0.40127042
## hu_has_aat_data1:is_active_training1         -0.82274955 0.86282641
## hu_has_aat_data1:UPPSsen                     -0.16403007 0.27072962
## hu_is_active_training1:UPPSsen               -0.42973923 0.61310153
## hu_has_aat_data1:is_active_training1:UPPSsen -0.32231496 0.34615354
```

```
## Intake - Subscale: UPPS Urgency
```

```
## Intake ~ has_aat_data * is_active_training * UPPSurg + (has_aat_data | vpNumber) + (has_aat_data * is_active_training | imgNumber) + (has_aat_data | vpNumber:imgNumber) 
## hu ~ has_aat_data * is_active_training * UPPSurg + (has_aat_data | vpNumber) + (has_aat_data * is_active_training | imgNumber) + (has_aat_data | vpNumber:imgNumber)
```

```
##                                                  Estimate  Est.Error
## Intercept                                     3.148020521 0.25343056
## hu_Intercept                                  1.121295907 0.58604000
## has_aat_data1                                 0.066262808 0.15039324
## is_active_training1                           0.308948407 0.34930757
## UPPSurg                                       0.104026083 0.10706841
## has_aat_data1:is_active_training1            -0.084151736 0.22117329
## has_aat_data1:UPPSurg                        -0.007360345 0.06377875
## is_active_training1:UPPSurg                  -0.145614648 0.15231597
## has_aat_data1:is_active_training1:UPPSurg     0.023188646 0.09587708
## hu_has_aat_data1                              0.038192668 0.34597646
## hu_is_active_training1                       -0.754584712 0.78460215
## hu_UPPSurg                                   -0.316140003 0.24838154
## hu_has_aat_data1:is_active_training1         -0.110285988 0.48304683
## hu_has_aat_data1:UPPSurg                     -0.027385767 0.14906648
## hu_is_active_training1:UPPSurg                0.346927016 0.34062548
## hu_has_aat_data1:is_active_training1:UPPSurg  0.082818419 0.20922301
##                                                     Q2.5     Q97.5
## Intercept                                     2.64281344 3.6483112
## hu_Intercept                                 -0.03152668 2.2452444
## has_aat_data1                                -0.22162630 0.3590576
## is_active_training1                          -0.38230517 1.0002739
## UPPSurg                                      -0.10639090 0.3144027
## has_aat_data1:is_active_training1            -0.51953572 0.3514929
## has_aat_data1:UPPSurg                        -0.13232885 0.1160277
## is_active_training1:UPPSurg                  -0.44495411 0.1531095
## has_aat_data1:is_active_training1:UPPSurg    -0.16509854 0.2107306
## hu_has_aat_data1                             -0.63596697 0.7161609
## hu_is_active_training1                       -2.23675828 0.8097324
## hu_UPPSurg                                   -0.79022661 0.1581700
## hu_has_aat_data1:is_active_training1         -1.04048953 0.8462716
## hu_has_aat_data1:UPPSurg                     -0.31822443 0.2634827
## hu_is_active_training1:UPPSurg               -0.33403556 0.9946560
## hu_has_aat_data1:is_active_training1:UPPSurg -0.33836454 0.4909269
```

**Increase foods:**

```
## Craving - Subscale: UPPS Perseverance
```

```
## Craving ~ has_aat_data * is_active_training * UPPSpers + (has_aat_data | vpNumber) + (has_aat_data * is_active_training | imgNumber) + (has_aat_data | vpNumber:imgNumber) 
## hu ~ has_aat_data * is_active_training * UPPSpers + (has_aat_data | vpNumber) + (has_aat_data * is_active_training | imgNumber) + (has_aat_data | vpNumber:imgNumber)
```

```
##                                                  Estimate  Est.Error
## Intercept                                      3.80954318 0.29306915
## hu_Intercept                                  -0.89093445 1.20623467
## has_aat_data1                                 -0.38089105 0.13824168
## is_active_training1                           -0.55802735 0.46776026
## UPPSpers                                      -0.12766703 0.09759248
## has_aat_data1:is_active_training1              0.39556468 0.21023941
## has_aat_data1:UPPSpers                         0.14015357 0.04717129
## is_active_training1:UPPSpers                   0.18106244 0.15267102
## has_aat_data1:is_active_training1:UPPSpers    -0.14828303 0.06955624
## hu_has_aat_data1                              -0.62930499 0.41764012
## hu_is_active_training1                        -0.38443496 1.93401627
## hu_UPPSpers                                    0.12482206 0.40050945
## hu_has_aat_data1:is_active_training1           1.33540039 0.67819335
## hu_has_aat_data1:UPPSpers                      0.15703391 0.13738891
## hu_is_active_training1:UPPSpers                0.08145756 0.63369305
## hu_has_aat_data1:is_active_training1:UPPSpers -0.41719690 0.22190297
##                                                      Q2.5        Q97.5
## Intercept                                      3.23923086  4.372334197
## hu_Intercept                                  -3.31792443  1.464763441
## has_aat_data1                                 -0.65347564 -0.110821907
## is_active_training1                           -1.46304582  0.364344376
## UPPSpers                                      -0.31744427  0.061812763
## has_aat_data1:is_active_training1             -0.01818812  0.812840224
## has_aat_data1:UPPSpers                         0.04698010  0.232945497
## is_active_training1:UPPSpers                  -0.12041520  0.474932383
## has_aat_data1:is_active_training1:UPPSpers    -0.28481848 -0.012149213
## hu_has_aat_data1                              -1.46260019  0.182161912
## hu_is_active_training1                        -4.16813626  3.398051144
## hu_UPPSpers                                   -0.65577776  0.915727678
## hu_has_aat_data1:is_active_training1           0.01913738  2.717560394
## hu_has_aat_data1:UPPSpers                     -0.11135415  0.429936961
## hu_is_active_training1:UPPSpers               -1.15285697  1.319682285
## hu_has_aat_data1:is_active_training1:UPPSpers -0.86279140  0.002605493
```

```
## Craving - Subscale: UPPS Premeditation
```

```
## Craving ~ has_aat_data * is_active_training * UPPSprem + (has_aat_data | vpNumber) + (has_aat_data * is_active_training | imgNumber) + (has_aat_data | vpNumber:imgNumber) 
## hu ~ has_aat_data * is_active_training * UPPSprem + (has_aat_data | vpNumber) + (has_aat_data * is_active_training | imgNumber) + (has_aat_data | vpNumber:imgNumber)
```

```
##                                                  Estimate  Est.Error
## Intercept                                      3.55446078 0.35599547
## hu_Intercept                                   0.63981470 1.40880375
## has_aat_data1                                  0.21744421 0.16637684
## is_active_training1                           -0.63410820 0.52801044
## UPPSprem                                      -0.03910207 0.11695091
## has_aat_data1:is_active_training1             -0.23093126 0.23978858
## has_aat_data1:UPPSprem                        -0.06415110 0.05391223
## is_active_training1:UPPSprem                   0.20088244 0.17215085
## has_aat_data1:is_active_training1:UPPSprem     0.06499782 0.07755126
## hu_has_aat_data1                               0.58766030 0.51487583
## hu_is_active_training1                        -1.01437929 2.07643926
## hu_UPPSprem                                   -0.38829520 0.46434770
## hu_has_aat_data1:is_active_training1          -0.05351924 0.77089688
## hu_has_aat_data1:UPPSprem                     -0.25334690 0.17033726
## hu_is_active_training1:UPPSprem                0.29832698 0.68041283
## hu_has_aat_data1:is_active_training1:UPPSprem  0.05324091 0.24879817
##                                                      Q2.5      Q97.5
## Intercept                                      2.86179973 4.26762271
## hu_Intercept                                  -2.13534949 3.42992110
## has_aat_data1                                 -0.11304860 0.53512556
## is_active_training1                           -1.65550102 0.44983068
## UPPSprem                                      -0.27076109 0.19135898
## has_aat_data1:is_active_training1             -0.70532118 0.24265913
## has_aat_data1:UPPSprem                        -0.16878610 0.04218291
## is_active_training1:UPPSprem                  -0.14858997 0.53182059
## has_aat_data1:is_active_training1:UPPSprem    -0.08868105 0.21832322
## hu_has_aat_data1                              -0.41811855 1.60279349
## hu_is_active_training1                        -5.06687651 3.07150453
## hu_UPPSprem                                   -1.29462507 0.52961924
## hu_has_aat_data1:is_active_training1          -1.58783120 1.43434072
## hu_has_aat_data1:UPPSprem                     -0.59169032 0.08540589
## hu_is_active_training1:UPPSprem               -1.04349173 1.62871769
## hu_has_aat_data1:is_active_training1:UPPSprem -0.41946457 0.55406410
```

```
## Craving - Subscale: UPPS Sensation Seeking
```

```
## Craving ~ has_aat_data * is_active_training * UPPSsen + (has_aat_data | vpNumber) + (has_aat_data * is_active_training | imgNumber) + (has_aat_data | vpNumber:imgNumber) 
## hu ~ has_aat_data * is_active_training * UPPSsen + (has_aat_data | vpNumber) + (has_aat_data * is_active_training | imgNumber) + (has_aat_data | vpNumber:imgNumber)
```

```
##                                                 Estimate  Est.Error        Q2.5
## Intercept                                     3.35939498 0.21465643  2.95052149
## hu_Intercept                                 -1.05825524 0.84477398 -2.64851498
## has_aat_data1                                 0.09382373 0.08741498 -0.07870794
## is_active_training1                           0.10368103 0.34739087 -0.55402735
## UPPSsen                                       0.03397232 0.08800318 -0.14185022
## has_aat_data1:is_active_training1             0.03389761 0.14927668 -0.24767222
## has_aat_data1:UPPSsen                        -0.03115403 0.03588308 -0.10226910
## is_active_training1:UPPSsen                  -0.05242102 0.13587545 -0.32116151
## has_aat_data1:is_active_training1:UPPSsen    -0.02239746 0.05813586 -0.13895470
## hu_has_aat_data1                              0.03525909 0.33363750 -0.62421175
## hu_is_active_training1                        2.24507070 1.38009244 -0.48645033
## hu_UPPSsen                                    0.22242836 0.34809776 -0.45084168
## hu_has_aat_data1:is_active_training1         -0.10074116 0.52701541 -1.17561028
## hu_has_aat_data1:UPPSsen                     -0.08706826 0.13458379 -0.34640715
## hu_is_active_training1:UPPSsen               -0.95106261 0.54153292 -1.99539907
## hu_has_aat_data1:is_active_training1:UPPSsen  0.08030710 0.20641743 -0.31945026
##                                                   Q97.5
## Intercept                                    3.78507029
## hu_Intercept                                 0.55714385
## has_aat_data1                                0.26652732
## is_active_training1                          0.78395534
## UPPSsen                                      0.20319566
## has_aat_data1:is_active_training1            0.33895933
## has_aat_data1:UPPSsen                        0.03919334
## is_active_training1:UPPSsen                  0.20994536
## has_aat_data1:is_active_training1:UPPSsen    0.09214980
## hu_has_aat_data1                             0.67695227
## hu_is_active_training1                       4.85516509
## hu_UPPSsen                                   0.89467600
## hu_has_aat_data1:is_active_training1         0.91353822
## hu_has_aat_data1:UPPSsen                     0.17345248
## hu_is_active_training1:UPPSsen               0.12351345
## hu_has_aat_data1:is_active_training1:UPPSsen 0.49457541
```

```
## Craving - Subscale: UPPS Urgency
```

```
## Craving ~ has_aat_data * is_active_training * UPPSurg + (has_aat_data | vpNumber) + (has_aat_data * is_active_training | imgNumber) + (has_aat_data | vpNumber:imgNumber) 
## hu ~ has_aat_data * is_active_training * UPPSurg + (has_aat_data | vpNumber) + (has_aat_data * is_active_training | imgNumber) + (has_aat_data | vpNumber:imgNumber)
```

```
##                                                 Estimate  Est.Error        Q2.5
## Intercept                                     3.13005793 0.27733210  2.58885587
## hu_Intercept                                  0.17178539 1.10611703 -1.97371158
## has_aat_data1                                 0.19016782 0.12053724 -0.04184072
## is_active_training1                           0.38971038 0.38361509 -0.36581051
## UPPSurg                                       0.13731689 0.11793212 -0.09473543
## has_aat_data1:is_active_training1            -0.33583917 0.16065050 -0.65143935
## has_aat_data1:UPPSurg                        -0.07395711 0.05156142 -0.17560482
## is_active_training1:UPPSurg                  -0.18324412 0.16660858 -0.50761352
## has_aat_data1:is_active_training1:UPPSurg     0.13498896 0.06931454  0.00138490
## hu_has_aat_data1                              0.20261917 0.39002235 -0.56399489
## hu_is_active_training1                       -0.41863860 1.56543663 -3.50942119
## hu_UPPSurg                                   -0.30600873 0.47409101 -1.23609990
## hu_has_aat_data1:is_active_training1         -0.53530826 0.57632691 -1.67790498
## hu_has_aat_data1:UPPSurg                     -0.16541171 0.16853606 -0.49964576
## hu_is_active_training1:UPPSurg                0.12253431 0.68000068 -1.21043104
## hu_has_aat_data1:is_active_training1:UPPSurg  0.27891615 0.25062388 -0.21176193
##                                                    Q97.5
## Intercept                                     3.67637651
## hu_Intercept                                  2.34050852
## has_aat_data1                                 0.42469180
## is_active_training1                           1.14144179
## UPPSurg                                       0.36604403
## has_aat_data1:is_active_training1            -0.02370424
## has_aat_data1:UPPSurg                         0.02569038
## is_active_training1:UPPSurg                   0.15070228
## has_aat_data1:is_active_training1:UPPSurg     0.27229093
## hu_has_aat_data1                              0.96989371
## hu_is_active_training1                        2.63295145
## hu_UPPSurg                                    0.63690407
## hu_has_aat_data1:is_active_training1          0.59073886
## hu_has_aat_data1:UPPSurg                      0.16456567
## hu_is_active_training1:UPPSurg                1.46027834
## hu_has_aat_data1:is_active_training1:UPPSurg  0.77543595
```

```
## Intake - Subscale: UPPS Perseverance
```

```
## Intake ~ has_aat_data * is_active_training * UPPSpers + (has_aat_data | vpNumber) + (has_aat_data * is_active_training | imgNumber) + (has_aat_data | vpNumber:imgNumber) 
## hu ~ has_aat_data * is_active_training * UPPSpers + (has_aat_data | vpNumber) + (has_aat_data * is_active_training | imgNumber) + (has_aat_data | vpNumber:imgNumber)
```

```
##                                                   Estimate  Est.Error
## Intercept                                      3.545225398 0.32279917
## hu_Intercept                                   0.881550233 0.75446348
## has_aat_data1                                 -0.028572737 0.21201389
## is_active_training1                            0.214022793 0.51383535
## UPPSpers                                      -0.058210006 0.10721554
## has_aat_data1:is_active_training1             -0.199658826 0.33323546
## has_aat_data1:UPPSpers                        -0.001585771 0.07086283
## is_active_training1:UPPSpers                  -0.084287297 0.16787988
## has_aat_data1:is_active_training1:UPPSpers     0.084026594 0.10927738
## hu_has_aat_data1                              -0.060702466 0.34300271
## hu_is_active_training1                        -0.994074781 1.17078779
## hu_UPPSpers                                   -0.073904019 0.25289851
## hu_has_aat_data1:is_active_training1           0.441557489 0.54729320
## hu_has_aat_data1:UPPSpers                      0.011184011 0.11485574
## hu_is_active_training1:UPPSpers                0.272138154 0.38375950
## hu_has_aat_data1:is_active_training1:UPPSpers -0.176865452 0.17877512
##                                                     Q2.5     Q97.5
## Intercept                                      2.9049280 4.1777866
## hu_Intercept                                  -0.5867032 2.3571697
## has_aat_data1                                 -0.4471589 0.3790801
## is_active_training1                           -0.7997385 1.2077081
## UPPSpers                                      -0.2693953 0.1528390
## has_aat_data1:is_active_training1             -0.8420339 0.4429243
## has_aat_data1:UPPSpers                        -0.1389888 0.1388435
## is_active_training1:UPPSpers                  -0.4089569 0.2462238
## has_aat_data1:is_active_training1:UPPSpers    -0.1279267 0.2965529
## hu_has_aat_data1                              -0.7371407 0.6037640
## hu_is_active_training1                        -3.2896831 1.3556048
## hu_UPPSpers                                   -0.5678954 0.4211531
## hu_has_aat_data1:is_active_training1          -0.6335890 1.5262777
## hu_has_aat_data1:UPPSpers                     -0.2111429 0.2387995
## hu_is_active_training1:UPPSpers               -0.4957780 1.0213363
## hu_has_aat_data1:is_active_training1:UPPSpers -0.5315983 0.1733333
```

```
## Intake - Subscale: UPPS Premeditation
```

```
## Intake ~ has_aat_data * is_active_training * UPPSprem + (has_aat_data | vpNumber) + (has_aat_data * is_active_training | imgNumber) + (has_aat_data | vpNumber:imgNumber) 
## hu ~ has_aat_data * is_active_training * UPPSprem + (has_aat_data | vpNumber) + (has_aat_data * is_active_training | imgNumber) + (has_aat_data | vpNumber:imgNumber)
```

```
##                                                  Estimate  Est.Error       Q2.5
## Intercept                                      3.60571987 0.38834817  2.8425299
## hu_Intercept                                   0.50598619 0.88165183 -1.2068919
## has_aat_data1                                  0.09398872 0.27448507 -0.4414536
## is_active_training1                           -0.47006664 0.56171260 -1.5914869
## UPPSprem                                      -0.07760435 0.12654145 -0.3263316
## has_aat_data1:is_active_training1             -0.06698818 0.37699726 -0.8120073
## has_aat_data1:UPPSprem                        -0.04214839 0.08996861 -0.2180267
## is_active_training1:UPPSprem                   0.13863579 0.18319130 -0.2215454
## has_aat_data1:is_active_training1:UPPSprem     0.04041347 0.12288976 -0.1990628
## hu_has_aat_data1                               0.08610478 0.42189471 -0.7412893
## hu_is_active_training1                        -0.69429796 1.32244600 -3.2066342
## hu_UPPSprem                                    0.05397196 0.28898870 -0.5281377
## hu_has_aat_data1:is_active_training1           0.35415302 0.62329839 -0.8466115
## hu_has_aat_data1:UPPSprem                     -0.03764343 0.13806547 -0.3008260
## hu_is_active_training1:UPPSprem                0.16706204 0.43154968 -0.6602046
## hu_has_aat_data1:is_active_training1:UPPSprem -0.14733570 0.20063062 -0.5515651
##                                                   Q97.5
## Intercept                                     4.3616984
## hu_Intercept                                  2.2362740
## has_aat_data1                                 0.6280369
## is_active_training1                           0.6344911
## UPPSprem                                      0.1704462
## has_aat_data1:is_active_training1             0.6773435
## has_aat_data1:UPPSprem                        0.1345464
## is_active_training1:UPPSprem                  0.4996442
## has_aat_data1:is_active_training1:UPPSprem    0.2862542
## hu_has_aat_data1                              0.8871880
## hu_is_active_training1                        1.8488319
## hu_UPPSprem                                   0.6212677
## hu_has_aat_data1:is_active_training1          1.6067509
## hu_has_aat_data1:UPPSprem                     0.2314160
## hu_is_active_training1:UPPSprem               1.0022935
## hu_has_aat_data1:is_active_training1:UPPSprem 0.2392614
```

```
## Intake - Subscale: UPPS Sensation Seeking
```

```
## Intake ~ has_aat_data * is_active_training * UPPSsen + (has_aat_data | vpNumber) + (has_aat_data * is_active_training | imgNumber) + (has_aat_data | vpNumber:imgNumber) 
## hu ~ has_aat_data * is_active_training * UPPSsen + (has_aat_data | vpNumber) + (has_aat_data * is_active_training | imgNumber) + (has_aat_data | vpNumber:imgNumber)
```

```
##                                                  Estimate  Est.Error
## Intercept                                     3.261487389 0.23380882
## hu_Intercept                                 -0.269024999 0.52672874
## has_aat_data1                                 0.029500713 0.14750329
## is_active_training1                           0.109994245 0.37899158
## UPPSsen                                       0.047899233 0.09472948
## has_aat_data1:is_active_training1             0.059084050 0.25010093
## has_aat_data1:UPPSsen                        -0.028043058 0.06098397
## is_active_training1:UPPSsen                  -0.067542454 0.14821438
## has_aat_data1:is_active_training1:UPPSsen     0.002109058 0.09866507
## hu_has_aat_data1                              0.399696384 0.24281540
## hu_is_active_training1                        1.626307544 0.85500546
## hu_UPPSsen                                    0.402162333 0.21245408
## hu_has_aat_data1:is_active_training1         -0.880488390 0.40068048
## hu_has_aat_data1:UPPSsen                     -0.185223815 0.09968469
## hu_is_active_training1:UPPSsen               -0.742756667 0.33475456
## hu_has_aat_data1:is_active_training1:UPPSsen  0.324190146 0.15700254
##                                                     Q2.5       Q97.5
## Intercept                                     2.80721812  3.71926685
## hu_Intercept                                 -1.30866538  0.74897209
## has_aat_data1                                -0.25588766  0.31847528
## is_active_training1                          -0.63937460  0.84894680
## UPPSsen                                      -0.13872259  0.23211721
## has_aat_data1:is_active_training1            -0.42409333  0.56200646
## has_aat_data1:UPPSsen                        -0.14838144  0.09058649
## is_active_training1:UPPSsen                  -0.35595384  0.22759286
## has_aat_data1:is_active_training1:UPPSsen    -0.19497002  0.19347479
## hu_has_aat_data1                             -0.07679975  0.87709397
## hu_is_active_training1                       -0.04610110  3.29326465
## hu_UPPSsen                                   -0.01236838  0.82120717
## hu_has_aat_data1:is_active_training1         -1.67751665 -0.10037185
## hu_has_aat_data1:UPPSsen                     -0.38414980  0.01105189
## hu_is_active_training1:UPPSsen               -1.39240413 -0.09089081
## hu_has_aat_data1:is_active_training1:UPPSsen  0.01785839  0.63239563
```

```
## Intake - Subscale: UPPS Urgency
```

```
## Intake ~ has_aat_data * is_active_training * UPPSurg + (has_aat_data | vpNumber) + (has_aat_data * is_active_training | imgNumber) + (has_aat_data | vpNumber:imgNumber) 
## hu ~ has_aat_data * is_active_training * UPPSurg + (has_aat_data | vpNumber) + (has_aat_data * is_active_training | imgNumber) + (has_aat_data | vpNumber:imgNumber)
```

```
##                                                  Estimate  Est.Error       Q2.5
## Intercept                                     3.285815715 0.30574185  2.7033561
## hu_Intercept                                  1.302308284 0.71836839 -0.1084546
## has_aat_data1                                -0.176573969 0.20040329 -0.5670441
## is_active_training1                          -0.036464286 0.42869378 -0.8906559
## UPPSurg                                       0.039754414 0.12991945 -0.2190395
## has_aat_data1:is_active_training1             0.290882020 0.26772894 -0.2311407
## has_aat_data1:UPPSurg                         0.062211524 0.08435092 -0.1037562
## is_active_training1:UPPSurg                  -0.006928973 0.18443106 -0.3760925
## has_aat_data1:is_active_training1:UPPSurg    -0.104948035 0.11485244 -0.3350839
## hu_has_aat_data1                              0.458705398 0.32873922 -0.1929264
## hu_is_active_training1                       -1.179398329 0.97039575 -3.0864308
## hu_UPPSurg                                   -0.277993272 0.30742798 -0.8853299
## hu_has_aat_data1:is_active_training1         -0.497466826 0.44804144 -1.3809422
## hu_has_aat_data1:UPPSurg                     -0.216508173 0.14081472 -0.4884018
## hu_is_active_training1:UPPSurg                0.444778790 0.42292547 -0.3799251
## hu_has_aat_data1:is_active_training1:UPPSurg  0.176461455 0.19518216 -0.1998447
##                                                   Q97.5
## Intercept                                    3.89087511
## hu_Intercept                                 2.74141971
## has_aat_data1                                0.21399114
## is_active_training1                          0.81030538
## UPPSurg                                      0.28616170
## has_aat_data1:is_active_training1            0.81593025
## has_aat_data1:UPPSurg                        0.22616202
## is_active_training1:UPPSurg                  0.36249795
## has_aat_data1:is_active_training1:UPPSurg    0.11857864
## hu_has_aat_data1                             1.09279627
## hu_is_active_training1                       0.68003396
## hu_UPPSurg                                   0.34796519
## hu_has_aat_data1:is_active_training1         0.36272162
## hu_has_aat_data1:UPPSurg                     0.05850439
## hu_is_active_training1:UPPSurg               1.27112592
## hu_has_aat_data1:is_active_training1:UPPSurg 0.55842008
```

## Analyses of craving and intake over the course of the study

We also analysed how craving and intake changed over the course of
the study. This analyses are based on hypothesis H4 of the
pre-registration where we predicted that the difference between the AAI
and AAA on food intake/craving increases over the period of the
intervention. However, we deviated from the pre-registered analysis by
using generalised additive models (GAMs).

**Decrease foods:**

```
## Craving
```

```
## Craving ~ s(study_day_n, by = is_active_training_f, k = -1) + is_active_training_f 
## hu ~ s(study_day_n, by = is_active_training_f, k = -1) + is_active_training_f
```

```
##                                            Estimate  Est.Error        Q2.5
## Intercept                                3.78404196 0.01050192  3.76359178
## hu_Intercept                            -0.69519453 0.02345161 -0.74213164
## is_active_training_f1                   -0.08445046 0.01510081 -0.11391774
## hu_is_active_training_f1                 0.13291603 0.03221197  0.07154636
## sstudy_day_n:is_active_training_f0_1    -0.66806046 0.45508640 -1.67223454
## sstudy_day_n:is_active_training_f1_1    -0.50417427 0.53765448 -1.47286994
## hu_sstudy_day_n:is_active_training_f0_1  1.43193084 0.93556555 -0.21652024
## hu_sstudy_day_n:is_active_training_f1_1  1.19665196 1.06126106 -0.52281105
##                                               Q97.5
## Intercept                                3.80447569
## hu_Intercept                            -0.65095744
## is_active_training_f1                   -0.05489805
## hu_is_active_training_f1                 0.19741677
## sstudy_day_n:is_active_training_f0_1     0.11236335
## sstudy_day_n:is_active_training_f1_1     0.75462423
## hu_sstudy_day_n:is_active_training_f0_1  3.65219573
## hu_sstudy_day_n:is_active_training_f1_1  3.68716854
```

```
## Intake
```

```
## Intake ~ s(study_day_n, by = is_active_training_f, k = -1) + is_active_training_f 
## hu ~ s(study_day_n, by = is_active_training_f, k = -1) + is_active_training_f
```

```
##                                              Estimate  Est.Error        Q2.5
## Intercept                                3.5824240448 0.01517124  3.55320245
## hu_Intercept                             0.1081679979 0.02132810  0.06728052
## is_active_training_f1                   -0.0006957872 0.02199844 -0.04475529
## hu_is_active_training_f1                 0.0237394354 0.03089526 -0.03901610
## sstudy_day_n:is_active_training_f0_1    -0.4765921200 0.55192563 -1.78796915
## sstudy_day_n:is_active_training_f1_1    -0.1453240475 0.44069042 -1.04377946
## hu_sstudy_day_n:is_active_training_f0_1  0.6055877342 0.81858458 -0.80105666
## hu_sstudy_day_n:is_active_training_f1_1 -1.7655216352 1.68693003 -5.58313031
##                                              Q97.5
## Intercept                               3.61272042
## hu_Intercept                            0.14993867
## is_active_training_f1                   0.04262945
## hu_is_active_training_f1                0.08351756
## sstudy_day_n:is_active_training_f0_1    0.49119798
## sstudy_day_n:is_active_training_f1_1    0.83402745
## hu_sstudy_day_n:is_active_training_f0_1 2.53218486
## hu_sstudy_day_n:is_active_training_f1_1 1.00355135
```

**Increase foods:**

```
## Craving
```

```
## Craving ~ s(study_day_n, by = is_active_training_f, k = -1) + is_active_training_f 
## hu ~ s(study_day_n, by = is_active_training_f, k = -1) + is_active_training_f
```

```
##                                            Estimate  Est.Error        Q2.5
## Intercept                                3.69660789 0.01162349  3.67406658
## hu_Intercept                            -0.29860643 0.02218592 -0.34204103
## is_active_training_f1                   -0.03902218 0.01698585 -0.07362724
## hu_is_active_training_f1                -0.07852493 0.03215197 -0.14095427
## sstudy_day_n:is_active_training_f0_1    -0.08912637 0.27769045 -0.63218244
## sstudy_day_n:is_active_training_f1_1    -0.01739205 0.37491172 -0.54574734
## hu_sstudy_day_n:is_active_training_f0_1  3.58335181 1.59901974  0.88703030
## hu_sstudy_day_n:is_active_training_f1_1  5.15559257 2.32902423  0.62331566
##                                               Q97.5
## Intercept                                3.71962084
## hu_Intercept                            -0.25527566
## is_active_training_f1                   -0.00554247
## hu_is_active_training_f1                -0.01603175
## sstudy_day_n:is_active_training_f0_1     0.59492630
## sstudy_day_n:is_active_training_f1_1     0.98334305
## hu_sstudy_day_n:is_active_training_f0_1  7.05267425
## hu_sstudy_day_n:is_active_training_f1_1  9.97228114
```

```
## Intake
```

```
## Intake ~ s(study_day_n, by = is_active_training_f, k = -1) + is_active_training_f 
## hu ~ s(study_day_n, by = is_active_training_f, k = -1) + is_active_training_f
```

```
##                                              Estimate  Est.Error        Q2.5
## Intercept                                3.5591629520 0.01757034  3.52369992
## hu_Intercept                             0.4526885999 0.02166821  0.41000709
## is_active_training_f1                    0.0937668696 0.02408811  0.04732393
## hu_is_active_training_f1                -0.2083098465 0.03099233 -0.26752789
## sstudy_day_n:is_active_training_f0_1    -0.2161252207 0.45131132 -1.27059485
## sstudy_day_n:is_active_training_f1_1    -0.4279198812 0.50818369 -1.67787859
## hu_sstudy_day_n:is_active_training_f0_1 -0.0004496422 0.49632896 -1.15449038
## hu_sstudy_day_n:is_active_training_f1_1 -0.2773214438 0.74375559 -1.70604667
##                                              Q97.5
## Intercept                                3.5928868
## hu_Intercept                             0.4950191
## is_active_training_f1                    0.1413825
## hu_is_active_training_f1                -0.1482490
## sstudy_day_n:is_active_training_f0_1     0.6350378
## sstudy_day_n:is_active_training_f1_1     0.4457251
## hu_sstudy_day_n:is_active_training_f0_1  0.9986275
## hu_sstudy_day_n:is_active_training_f1_1  1.4245600
```
